# CD20^+^ T follicular helper–like cells drive antigen-specific autoimmunity in bullous pemphigoid

**DOI:** 10.1172/JCI197665

**Published:** 2026-07-07

**Authors:** Hui Fang, Shengxian Shen, Kang Li, Tianyu Cao, Bing Wang, Haijun Miao, Ke Xue, Yaxing Bai, Liang Li, Xia Li, Pei Qiao, Jieyu Zhang, Huanhuan Qu, Chen Zhang, Chunying Xiao, Bingyu Pang, Meng Fu, Hongjiang Qiao, Shuai Shao, Erle Dang, Gang Wang

**Affiliations:** 1 Department of Dermatology, Xijing Hospital, Fourth Military Medical University, Xi’an, Shaanxi, China.; 2 Department of Dermatology, Tangdu Hospital, Fourth Military Medical University, Xi’an, Shaanxi, China.

**Keywords:** Autoimmunity, Dermatology, Autoimmune diseases, T cells

## Abstract

CD20^+^ T cells are increasingly recognized as drivers of autoimmune and inflammatory diseases. However, their origin, development, and specific role in autoimmune skin diseases remain poorly understood. In this study, we observed an expansion of CD20^+^ T cells in the peripheral blood and skin lesions of patients with bullous pemphigoid (BP), which correlated with the levels of pathogenic autoantibodies and disease severity. Compared with CD20^–^ T cells, CD20^+^ T cells exhibited enhanced metabolic and pro-inflammatory activities. In particular, antigen-specific BP180-NC16A–reactive T cells were enriched within the CD4^+^CD20^+^ subset. In both patients with BP and BP180-immunized mice, CD4^+^CD20^+^ T cells exhibited an antigen-specific T follicular helper–like phenotype, facilitating antibody production and B cell differentiation, whereas CD8^+^CD20^+^ T cells displayed cytotoxic and pro-inflammatory features. Mechanistically, we found that expression of the CD20-encoding gene *MS4A1* in T cells was regulated by transcription factor PAX5 in a DNA methylation–dependent manner. Therefore, our study elucidates the regulatory mechanisms governing CD20^+^ T cells and highlights their important role in the pathogenesis of BP.

## Abstract 2

<span>CD20⁺ T cells with Tfh-like properties promote autoantibody production, and </span> <span>the transcription factor </span> <span>PAX5 regulates their differentiation through DNA methylation.</span>

## Graphical abstract

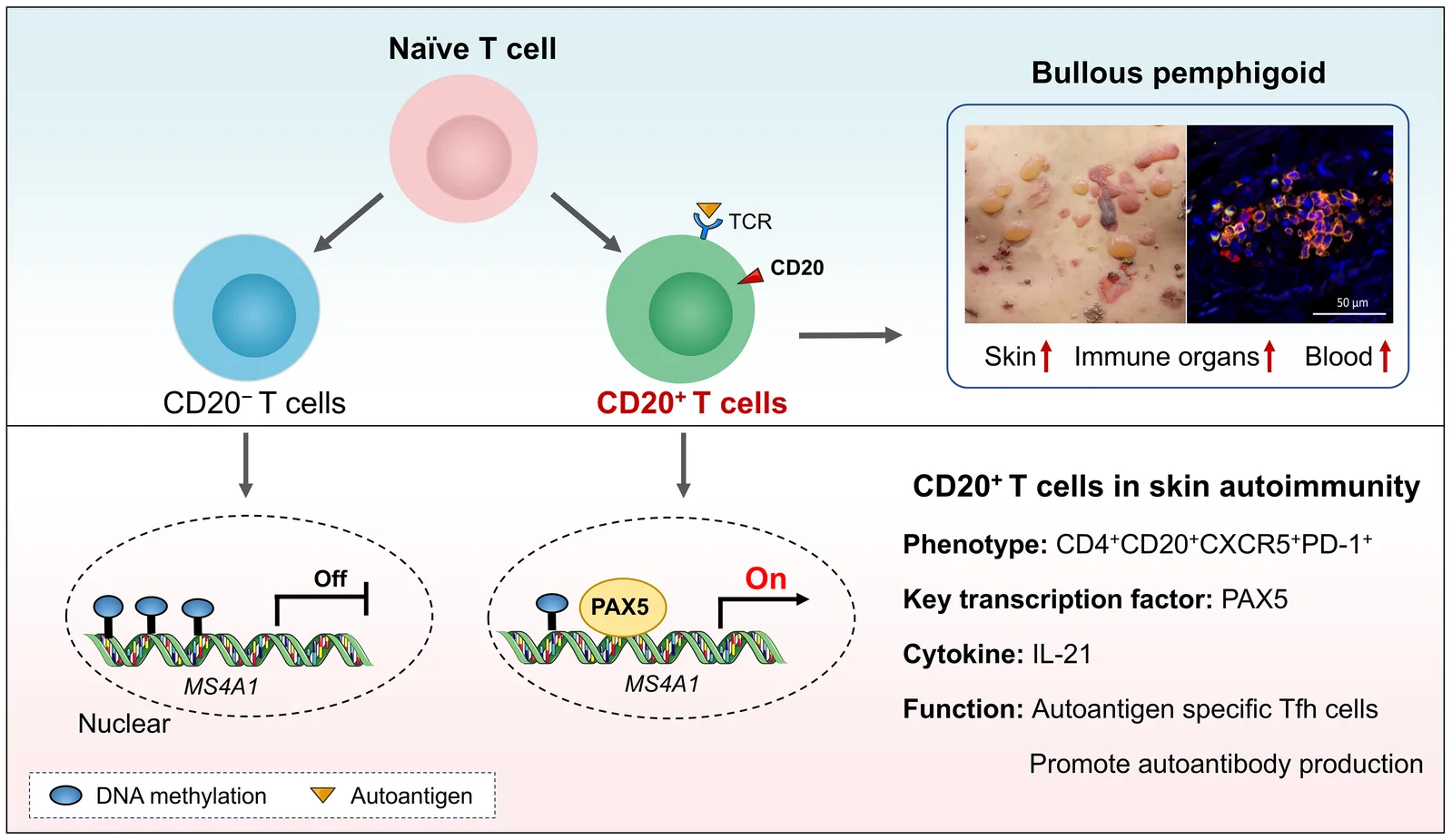

## Introduction

CD20 is a surface molecule classically expressed on B lymphocytes from early developmental stages through maturity, but it is absent on terminally differentiated plasmablasts and plasma cells (1). Over 2 decades ago, a subset of human CD3^+^ T cells in the peripheral blood was shown to express CD20, albeit at low levels compared with those of B cells (2). Recent research has increasingly highlighted the role of these CD20^+^ T cells in various autoimmune diseases, such as multiple sclerosis (3–5), rheumatoid arthritis (6, 7), primary Sjögren’s syndrome (8), and psoriasis (9), as well as obesity (10) and cancer (11). Notably, therapies targeting CD20, such as rituximab (RTX), deplete both CD20^+^ B cells and CD20^+^ T cells, effectively reducing inflammation in patients with diseases such as multiple sclerosis (12). Despite these findings, the origins and functions of CD20^+^ T cells in autoimmune skin diseases remain incompletely understood.

Autoimmune diseases are characterized by aberrant interactions between T cells and B cells, leading to the production of autoantibodies. T cells play pivotal roles in autoimmune pathogenesis by promoting B cell survival, differentiation, and antibody production, as well as by secreting pro-inflammatory cytokines (13, 14). Bullous pemphigoid (BP), a common autoimmune blistering skin disorder, is characterized by autoantibodies targeting proteins at the dermal-epidermal junction (BP180 and BP230), which clinically manifests as tense blisters and erosions on the skin surface (15). Our previous study identified 2 major T cell epitope peptides, P2 (492–506 aa: VRKLKARVDELERIR) and P5 (501–515 aa: ELERIRRSILPYGDS), along with highlighting their substantial roles in stimulating CD4^+^ T cell proliferation, IL-4 production, and activation of autoantibody-secreting B cells (16). Additionally, multiple T cell abnormalities have been documented in BP, including expansion of activated T helper 2 (Th2) cells (17), Th17 cells (18), and T follicular helper (Tfh) cells (19), as well as dysfunction of regulatory T cells (20, 21). However, the specific contributions of CD20^+^ T cells to the pathogenesis of BP remain unclear.

In the present study, we investigated CD20^+^ T cells in peripheral blood and skin lesions from patients with BP, as well as in BP180-immunized mice. We found that CD20^+^ T cells were markedly expanded in both the circulation and lesional skin of patients with BP and that this expansion correlated with disease severity. Compared with CD20^–^ T cells, CD20^+^ T cells displayed enhanced proliferative capacity, autoreactivity to BP autoantigens, and increased metabolic activity. In both patients with BP and BP180-immunized mice, CD4^+^CD20^+^ T cells exhibited an antigen-specific Tfh-like phenotype, characterized by heightened responsiveness to BP autoantigens, increased IL-21 production, and a greater capacity to promote B cell differentiation and autoantibody generation, whereas CD8^+^CD20^+^ T cells displayed predominantly cytotoxic and pro-inflammatory features. Mechanistically, we further demonstrated that *MS4A1*, the gene encoding CD20, is endogenously expressed in human T cells and is regulated by DNA methylation and the transcription factor PAX5. Together, these findings identify functionally distinct CD20^+^ T cell subsets in BP and provide insight into the regulatory mechanisms underlying CD20 expression and pathogenic T cell function.

## Results

### CD20^+^ T cells expand in the peripheral blood and skin lesions of patients with BP.

**Figure 1:**
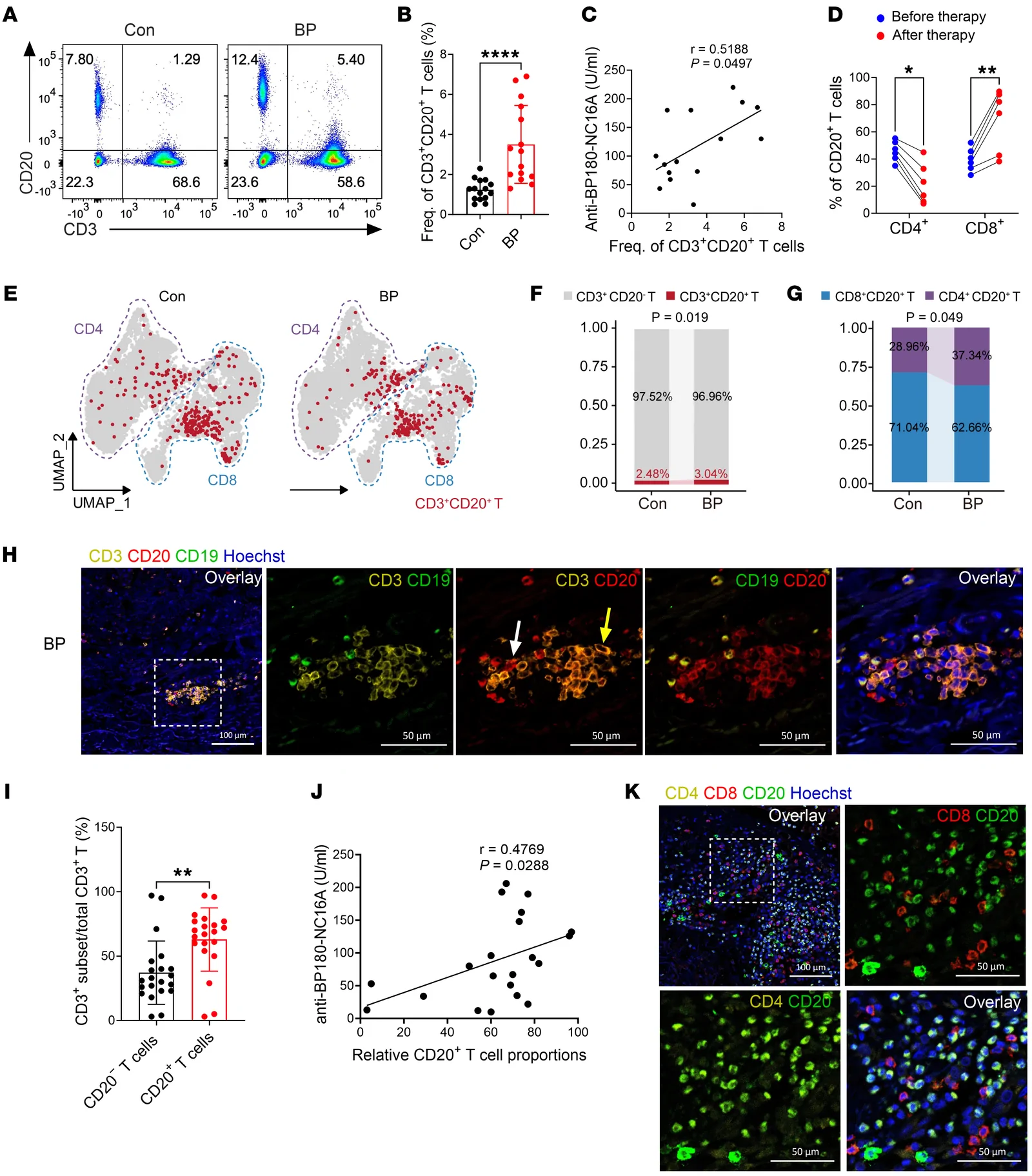
Increased levels of CD20^+^ T cells in patients with BP. (**A**) Representative flow cytometry dot plot showing CD3^+^CD20^+^ T cell identification in peripheral blood. (**B**) Frequencies of CD3^+^CD20^+^ T cells in PBMCs from healthy controls (Con; *n* = 15) and patients with BP (*n* = 15). (**C**) Correlation between the proportions of circulating CD3^+^CD20^+^ T cells and serum anti–BP180-NC16A antibody titers in patients with BP (*n* = 15). (**D**) Frequencies of CD4^+^CD20^+^ and CD8^+^CD20^+^ T cells before and after glucocorticoid treatment in patients with BP (*n* = 6). (**E**) Uniform manifold approximation and projection (UMAP) visualization of CD3^+^CD20^+^ T cells and subsets (CD4^+^ and CD8^+^) in Con and Patients with BP. (**F**) Bar charts showing the proportions of CD3^+^CD20^+^ T cells in Con and patients with BP. (**G**) Bar charts showing the proportions of CD4^+^CD20^+^ and CD8^+^CD20^+^ T cells in Con and patients with BP. (**H**) Representative images of immunofluorescence staining for CD3 (yellow), CD19 (green), CD20 (red), and DNA (Hoechst, blue) in the skin lesions of patients with BP. Scale bar = 100 μm; scale bar inset = 50 μm. (**I**) Relative proportions of CD3^+^CD20^+^ and CD3^+^CD20^–^ T cells in the skin lesions of individual patients with BP (*n* = 21). (**J**) Correlation between the relative proportions of CD3^+^CD20^+^ T cells in skin lesions and anti–BP180-NC16A antibody titers in patients with BP (*n* = 21). (**K**) Representative images of immunofluorescence staining for CD4 (yellow), CD8 (red), CD20 (green), and DNA (Hoechst, blue) in BP skin lesions (*n* = 8). Scale bar = 100 μm; scale bar inset = 50 μm. All data are presented as the mean ± SD. An unpaired 2-tailed Student’s *t* test was used for **B** and **I**; Spearman’s correlation was used for **C** and **J**; χ^2^ test was used for **F** and **G**; a paired Student’s *t* test was used for **D**. *P < 0.05, **P < 0.01, ****P < 0.0001.

To explore the potential involvement of CD3^+^CD20^+^ T cells in BP pathogenesis, we compared their levels in patients with BP and sex- and age-matched healthy controls via flow cytometry. Our analyses revealed significantly higher percentages of CD3^+^CD20^+^ T cells in the peripheral blood of patients with BP than in that of healthy controls (Figure 1, A and B). Additionally, the frequencies of these CD20^+^ T cells were significantly positively correlated with the serum titer of anti–BP180-NC16A IgG, a key biomarker of disease severity (Figure 1C). Previous studies have shown that CD20^+^ T cells encompass both CD4^+^ and CD8^+^ subpopulations (3). Our analysis of gated CD3^+^CD20^+^ T cells in the circulation revealed an increase in the CD4^+^CD20^+^ T cell subpopulation in patients with BP compared with healthy controls, though the difference was not statistically significant (Supplemental Figure 1, A and B; supplemental material available online with this article; https://doi.org/10.1172/JCI197665DS1). After corticosteroid treatment, which is typically the initial therapy for BP, there was no significant change in the overall frequency of CD3^+^CD20^+^ T cells (Supplemental Figure 1, C and D). However, we observed a shift in the CD4^+^ and CD8^+^ subpopulations within CD20^+^ T cells after corticosteroid treatment: the proportion of CD4^+^CD20^+^ T cells decreased, whereas that of CD8^+^CD20^+^ T cells increased (Figure 1D and Supplemental Figure 1, D and E).

To further investigate the circulating immunological changes in BP, we performed single-cell RNA sequencing (scRNA-seq) on immune cells isolated from 3 patients with active BP and 3 age- and sex-matched healthy controls (Supplemental Figure 2, A and B). Following quality control, a total of 52,794 high-quality cells (25,073 from healthy controls and 27,721 from BP patients) were used for further analysis. Using a standard single-cell analysis pipeline, we identified 15 distinct clusters (Supplemental Figure 2C). On the basis of canonical marker gene expression, these clusters were classified into 7 major immune cell types: T cells, B cells, natural killer (NK) cells, monocytes, granulocytes, megakaryocytes, and dendritic cells (Supplemental Figure 2, D and E). Comparative analysis revealed marked shifts in immune cell proportions, including reduced levels of T cells and NK cells and increased proportions of monocytes and granulocytes, in patients with BP compared with healthy controls (Supplemental Figure 2F). Consistent with the flow cytometry data, our scRNA-seq analysis revealed a T cell subset expressing the classical B cell marker gene *MS4A1* in both patients with BP and healthy controls (Figure 1E). Cluster proportion analysis revealed that an increased proportion of CD3^+^CD20^+^ T cells was accompanied by a corresponding decrease in CD3^+^CD20^–^ T cells in BP (Figure 1F). Quantification revealed that CD8^+^CD20^+^ T cells constituted a substantial proportion of T cells in both groups. Strikingly, the proportion of CD4^+^CD20^+^ T cells was significantly greater in the BP group than in the healthy control group (Figure 1G).

Next, we investigated whether CD20^+^ T cells were also present in the inflamed skin tissues of BP and other skin diseases. Confocal imaging revealed the colocalization of CD20 with the classic T cell marker CD3 and the B cell marker CD19, confirming the presence of both CD3^+^CD20^+^ T cells and CD19^+^CD20^+^ B cells in BP skin lesions (Figure 1H). Interestingly, we also detected the presence of CD3^+^CD20^+^ T cells in blister fluid (BF), a body fluid that is specifically enriched with innate and adaptive immune cells in BP (Supplemental Figure 3, A and B). Notably, CD3^+^CD20^+^ T cells were absent in lesions from patients with other autoimmune and inflammatory skin conditions, such as pemphigus vulgaris, psoriasis, and lichen planus (Supplemental Figure 3B). Among the CD3^+^ T cells infiltrating BP lesions, CD3^+^CD20^+^ T cells were more prevalent than CD3^+^CD20^–^ T cells (Figure 1I and Supplemental Figure 4, A and B). Furthermore, the abundance of CD3^+^CD20^+^ T cells in skin lesions was positively correlated with the titers of anti-BP180 NC16A antibodies (Figure 1J). Immunofluorescence analysis further demonstrated that CD4^+^CD20^+^ T cells, not CD8^+^CD20^+^ T cells, constituted the major infiltrating CD20^+^ T cell subpopulation in BP skin lesions (Figure 1K). Collectively, these results demonstrate that the expansion of CD20^+^ T cells in the peripheral blood and skin lesions of patients with BP is correlated with disease severity.

### CD20^+^ T cells are the predominant BP180-NC16A antigen-specific T cells with enhanced metabolic and pro-inflammatory activities in BP.

To gain deeper insights into the functions and transcriptional profile of CD20^+^ T cells, we sorted and performed RNA-sequencing (RNA-seq) analysis on CD3^+^CD20^+^ T cells, CD3^+^CD20^–^ T cells, and CD19^+^ B cells from patients with BP and sex- and age-matched healthy controls (*n* = 3 per group; sorting strategy shown in Supplemental Figure 5A). Our analysis revealed a total of 2,062 and 2,485 differentially expressed genes (DEGs) (fold-change [FC] ≥ 1.5, *P* < 0.05) between CD19^+^ B cells and CD3^+^CD20^+^ T cells from patients with BP and healthy controls, respectively (Supplemental Figure 5B). Notably, CD3^+^CD20^+^ T cells presented upregulated expression of surface markers such as *CD3*, *CD4*, *CD5*, *CD8*, and *CD28* but downregulated expression of *CD40*, *CD74*, *CD79A*, and *CD79B*. Importantly, compared with CD19^+^ B cells, CD20^+^ T cells did not express *CD19*, *CD86*, or *CD22*, suggesting their lineage-specific differences from B cells (Supplemental Figure 5C).

**Figure 2:**
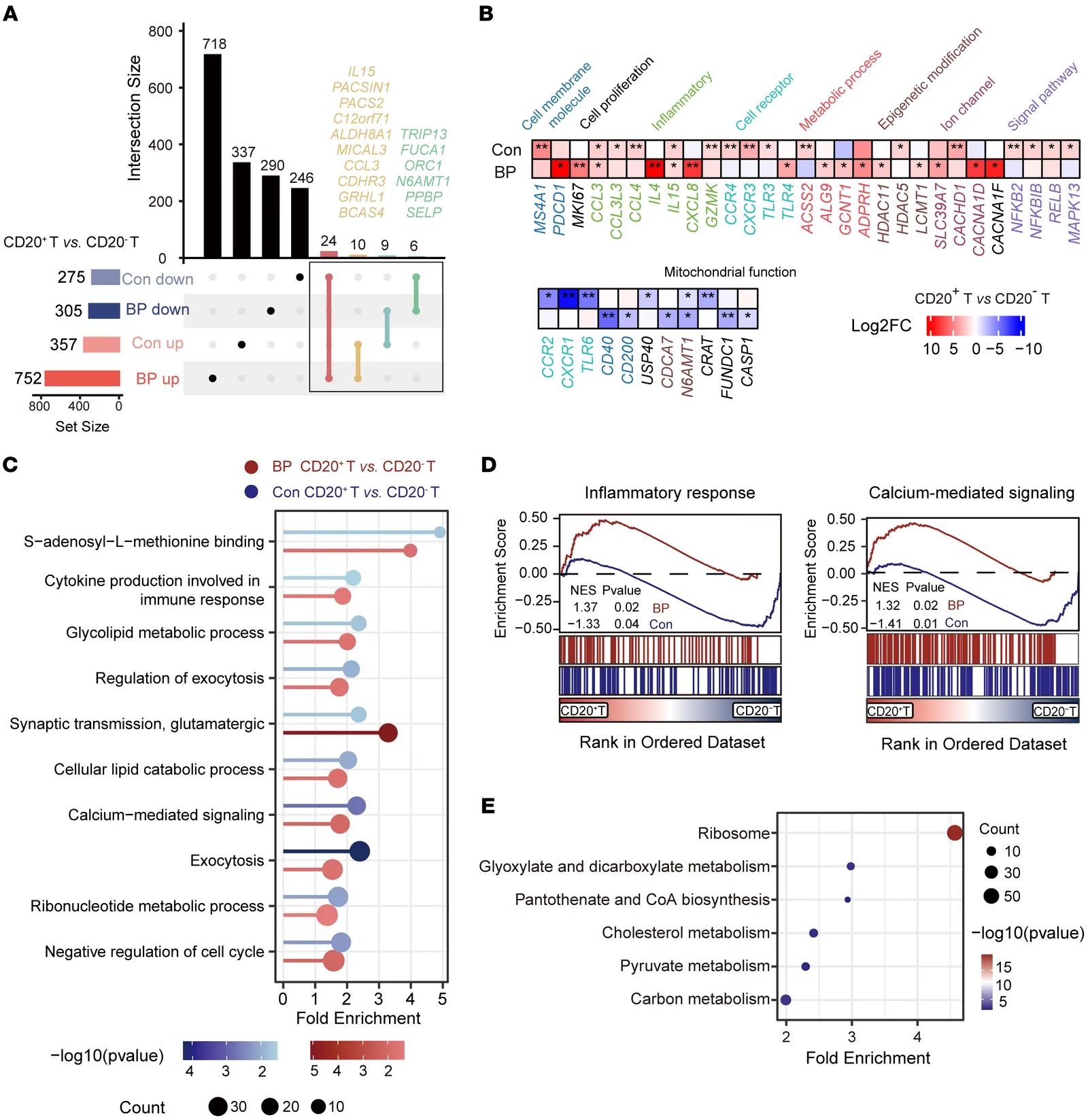
RNA-sequencing analysis of CD20^+^ T cells in the peripheral blood of humans. CD3^+^CD20^+^ T cells, and CD3^+^CD20^–^ T cells were sorted from 3 patients with BP and 3 sex- and age-matched Con. (**A**) UpSet plot showing the overlap of differentially expressed genes (DEGs; upregulated and downregulated) between CD20^+^ T and CD20^–^ T cells in Con and patients with BP. (**B**) Heatmap of DEGs between CD20^+^ T cells and CD20^–^ T cells from Con and patients with BP. (**C**) Gene Ontology (GO) enrichment analysis of DEGs between CD20^+^ and CD20^–^ T cells in patients with BP and Con. (**D**) Gene set enrichment analysis (GSEA) showing enrichment of gene sets associated with inflammatory response and calcium-mediated signaling in CD20^+^ T cells. (**E**) Kyoto Encyclopedia of Genes and Genomes (KEGG) pathway enrichment of DEGs between CD20^+^ T cells from patients with BP and those from Con. *P < 0.05, **P < 0.01. DESeq2 with Wald test was used for differential expression analysis in **B**.

By comparing CD3^+^CD20^+^ T cells and CD3^+^CD20^–^ T cells between patients with BP and healthy controls, we identified 752 upregulated and 305 downregulated genes between CD3^+^CD20^+^ T cells and CD3^+^CD20^–^ T cells from patients with BP. In healthy controls, compared with CD3^+^CD20^–^ T cells, CD3^+^CD20^+^ T cells presented 357 upregulated genes and 275 downregulated genes (Figure 2A). In patients, CD3^+^CD20^+^ T cells presented elevated expression levels of genes associated with various biological functions, including cell proliferation (e.g., *MKI67*), cytokine and chemokine production (e.g., *IL15*, *CXCL8*, *CCL3*), granular protease activity (e.g., *GZMK*), metabolic processes (e.g., *ALG9*, *GCNT1*, *ADPRH*), epigenetic modifications (e.g., *LCMT1*), transporters (e.g., *SLC39A7*), and voltage-gated calcium channels (e.g., *CACNA1D*), compared with CD3^+^CD20^–^ T cells (Figure 2B). Gene ontology (GO) analysis further demonstrated enrichment of pathways related to S-adenosyl-l-methionine binding, cytokine production involved in the immune response, glycolipid metabolic processes, and cellular calcium ion homeostasis in CD20^+^ T cells from both BP and healthy controls (Figure 2C). Consistent with these findings, gene set enrichment analysis (GSEA) showed statistically significant enrichment of genes associated with the inflammatory response and calcium-mediated signaling in CD20^+^ T cells compared with those in CD20^–^ T cells (Figure 2D). Among all the upregulated genes, Kyoto Encyclopedia of Genes and Genomes (KEGG) analysis revealed enrichment of metabolic pathways, such as glyoxylate and dicarboxylate metabolism, carbon metabolism, pyruvate metabolism, and cholesterol metabolism, specifically in BP CD20^+^ T cells, underscoring their heightened metabolic activity under disease conditions (Figure 2E).

**Figure 3:**
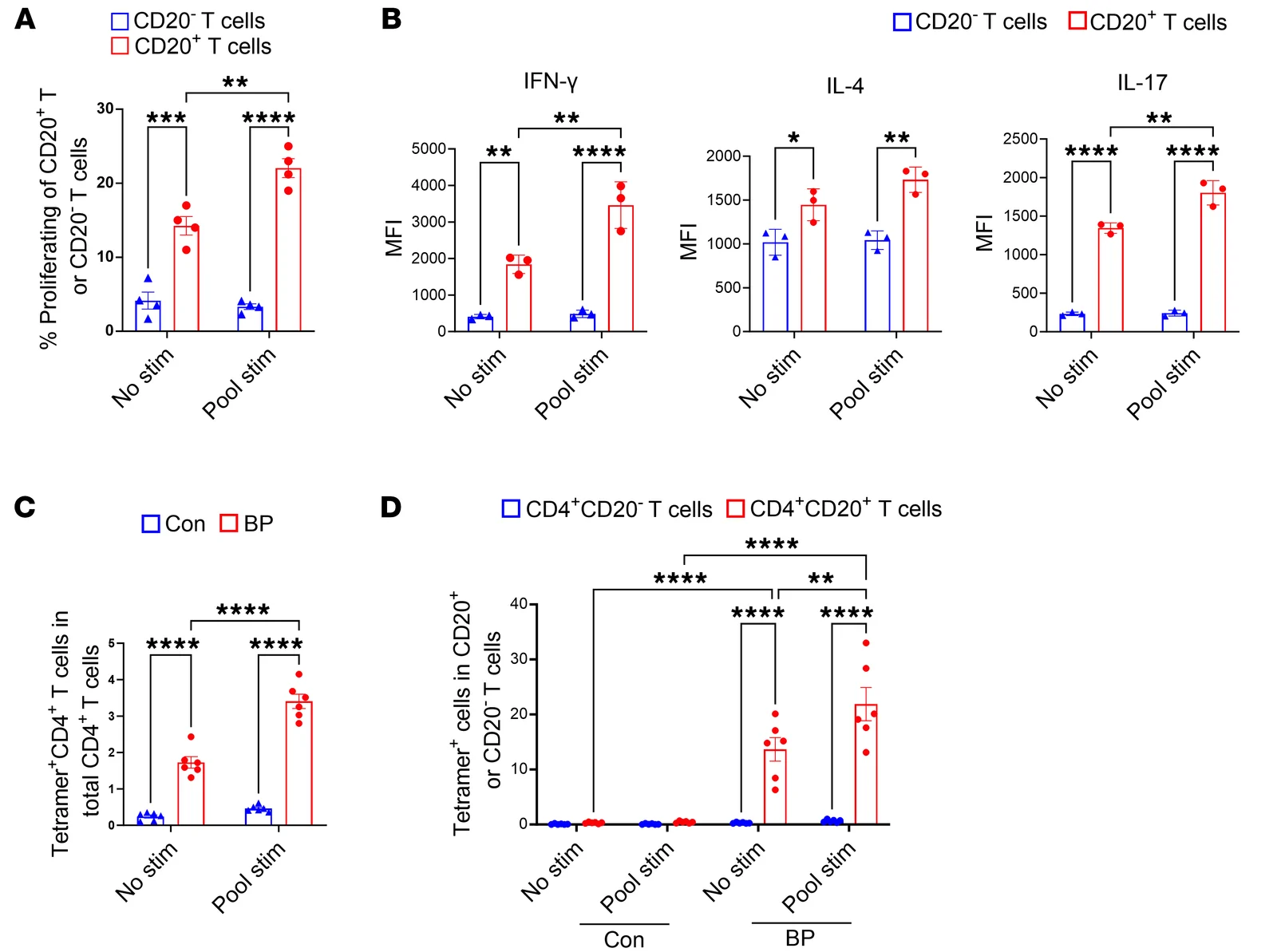
CD20^+^ T cells exhibit antigen-specific responses in patients with BP. (**A**) Percentages of proliferating CD20^+^ and CD20^–^ T cells from patients with BP with or without peptide stimulation (*n* = 4). (**B**) Mean fluorescence intensity (MFI) of IFN-γ, IL-4, and IL-17 expression in CD20^+^ and CD20^–^ T cells from patients with BP under unstimulated or peptide-stimulated conditions (*n* = 3). (**C**) Frequencies of tetramer-positive CD4^+^ T cells in PBMCs collected from untreated patients with BP (*n* = 6) and HLA allele-matched healthy controls (*n* = 6). (**D**) Frequencies of tetramer-positive T cells within gated CD4^+^CD20^+^ and CD4^+^CD20^–^ T cell subsets from PBMCs of untreated patients with BP (*n* = 6) and HLA allele-matched healthy controls (*n* = 6), with or without peptide stimulation. All data are presented as the mean ± SD. Two-way ANOVA with Tukey’s post hoc test was used for **A**–**D**. *P < 0.05, **P < 0.01, ***P < 0.001, ****P < 0.0001.

To assess the functional responsiveness of CD20^+^ T cells to BP autoantigens, we compared the proliferative and cytokine responses of CD20^+^ and CD20^–^ T cell subsets following stimulation with BP180-NC16A peptides. We found that compared with CD20^–^ T cells, CD20^+^ T cells displayed increased proliferative capacity when stimulated with peptides in vitro (Figure 3A and Supplemental Figure 6A). Previous research has highlighted the pro-inflammatory role of CD20^+^ T cells, which produce cytokines such as IFN-γ and TNF-α in diverse autoimmune contexts (4, 22). Notably, intracellular staining showed that the CD20^+^ T cells produced significant amounts of IFN-γ, IL-4, and IL-17 with or without peptide stimulation when compared with CD20^–^ T cells, suggesting the enhanced pro-inflammatory abilities of CD20^+^ T cells in BP (Figure 3B and Supplemental Figure 6B). We also found that stimulation of T cells with anti-CD3/CD28 Dynabeads for 72 hours increased CD20 expression, suggesting that TCR activation promotes CD20 upregulation in T cells (Supplemental Figure 7, A–D).

Our previous research identified key HLA-DR–restricted T cell epitopes within BP180-NC16A, such as P5 (501–515 aa: ELERIRRSILPYGDS), which play pivotal roles in BP pathogenesis (16). To further assess the antigen specificity of CD20^+^ T cells in BP, we developed a peptide-MHC class II (pMHC II) tetramer to detect these cells ex vivo in the peripheral blood of untreated patients with BP and HLA allele-matched healthy controls. Flow cytometry analysis revealed a greater proportion of tetramer-positive CD4^+^ T cells in patients with BP than in healthy controls under resting conditions (Figure 3C and Supplemental Figure 8A), suggesting prior antigen exposure in BP. Upon stimulation with pooled peptides, the frequency of tetramer-positive CD4^+^ T cells increased further compared with nonstimulated PBMCs from the same patients (Figure 3C and Supplemental Figure 8A). Notably, when comparing tetramer-positive cells within the CD4^+^CD20^+^ and CD4^+^CD20^–^ subsets, we found a markedly higher frequency of tetramer-positive CD4^+^CD20^+^ T cells in patients with BP than in healthy controls, both with and without peptide stimulation (Figure 3D and Supplemental Figure 8B), indicating a greater proportion of antigen-specific T cells among the CD20^+^ T cells. These findings demonstrate that in BP, antigen-specific T cells are enriched within CD20^+^ T cells, which exhibit heightened responsiveness to autoantigens and enhanced proliferation and elevated metabolic activity.

### CD4^+^CD20^+^ T cells exhibit characteristics of Tfh-like cells and produce high levels of IL-21 in BP.

**Figure 4:**
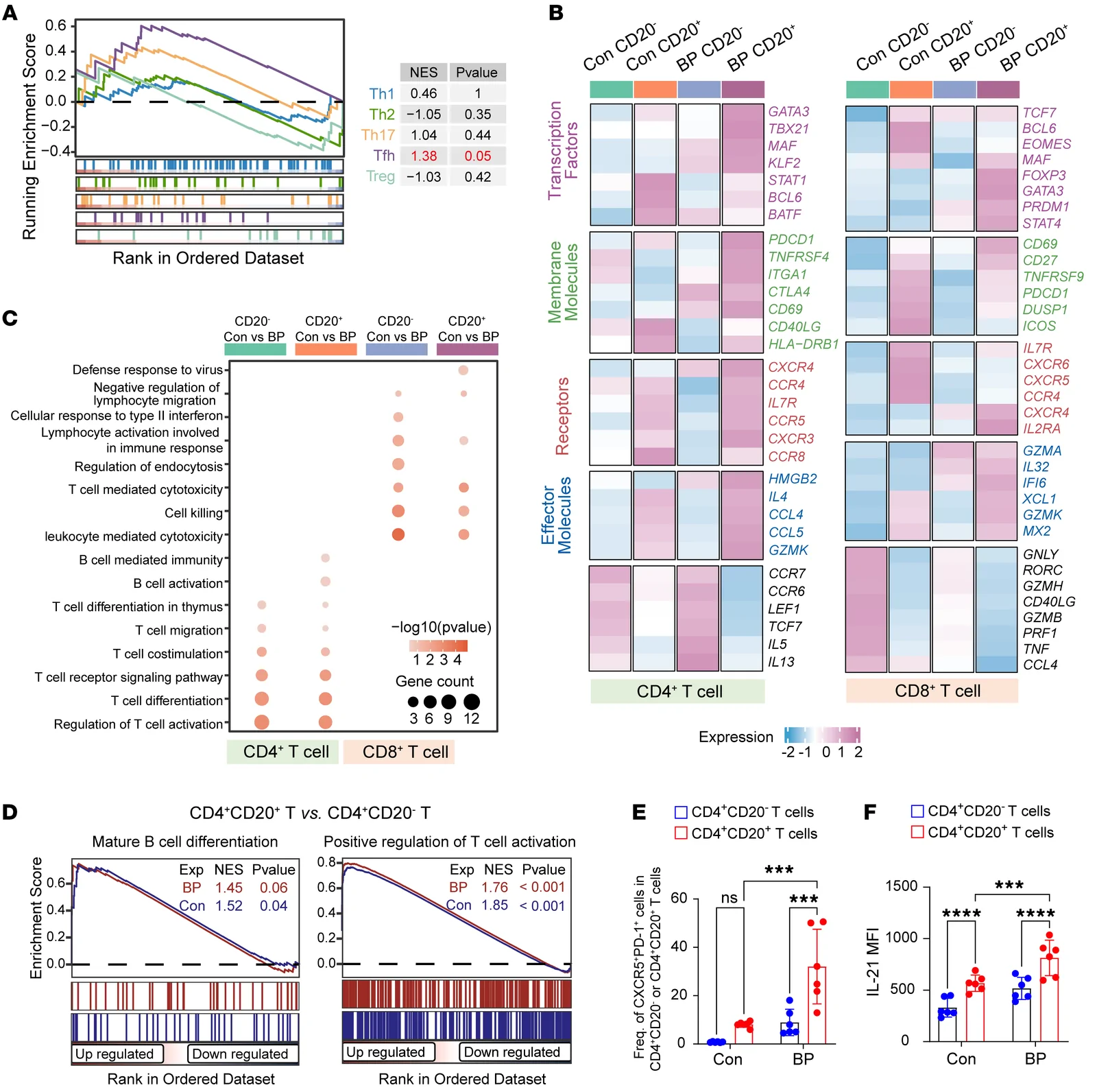
CD4^+^CD20^+^ T cells exhibit Tfh-like characteristics in BP. (**A**) GSEA enrichment plots for hallmark T cell subsets, comparing CD3^+^CD20^+^ T cells to CD3^+^CD20^–^ T cells in patients with BP. (**B**) ScRNA-seq analysis of CD4^+^CD20^+^ and CD8^+^CD20^+^ T cells from patients with BP and Con. Heatmap showing DEGs comparing CD4^+^CD20^+^ versus CD4^+^CD20^–^ T cells, and CD8^+^CD20^+^ versus CD8^+^CD20^–^ T cells, between Con and patients with BP. (**C**) GO analysis of DEGs enriched in the indicated T cell subsets between Con and patients with BP. (**D**) GSEA showing enrichment of genes related to mature B cell differentiation and positive regulation of T cell activation in CD4^+^CD20^+^ T cells. (**E**) Frequencies of CXCR5^+^PD-1^+^ T cells within CD4^+^CD20^+^ and CD4^+^CD20^–^ T cells in Con and patients with BP (*n* = 6). (**F**) MFI of IL-21 expression in CD4^+^CD20^+^ versus CD4^+^CD20^–^ T cells from Con and patients with BP (*n* = 6). All data are presented as the mean ± SD. Two-way ANOVA with Tukey’s post hoc test was used for **E** and **F**. ***P < 0.001, ****P < 0.0001.

To further investigate the functional polarization of CD3^+^CD20^+^ T cells in BP, we performed GSEA pathway analysis on this subset. Our bulk RNA-seq data revealed significant enrichment of genes associated with Tfh cell differentiation in CD3^+^CD20^+^ cells compared with CD3^+^CD20^–^ T cells in BP (Figure 4A). Given that Tfh cells are primarily a CD4^+^ T cell subset, we next performed more detailed analyses of CD4^+^CD20^+^ and CD8^+^CD20^+^ T cells. ScRNA-seq analysis showed that CD4^+^CD20^+^ T cells exhibited higher expression of membrane molecules (e.g., *PDCD1*, *CD40LG*, *CD69*, *TNFRSF4*), receptors (*CCR4*, *CXCR3*), effector molecules (*IL4*, *CCL4*, *GZMK*), and transcription factors (*MAF*, *BCL6*, and *GATA3*) than CD4^+^CD20^–^ T cells. Compared with CD8^+^CD20^–^ T cells, CD8^+^CD20^+^ T cells displayed higher expression of T cell activation and costimulatory molecules (*CD69*, *TNFRSF9*, *CD27*, *PDCD1*, *ICOS*), as well as *GZMK* (Figure 4B). Consistent with these transcriptional profiles, GO analysis revealed that CD4^+^CD20^+^ T cells were enriched in biological processes including B cell activation, T cell migration, T cell differentiation, and regulation of T cell activation, whereas CD8^+^CD20^+^ T cells were enriched in functions related to defense response to virus and T cell mediated cytotoxicity (Figure 4C). GSEA further demonstrated significant enrichment of mature B cell differentiation and positive regulation of T cell activation in CD4^+^CD20^+^ T cells, whereas T cell mediated cytotoxicity and inflammatory cytokine production were preferentially enriched in CD8^+^CD20^+^ T cells (Figure 4D and Supplemental Figure 9).

Next, we examined the secretion of cytokines and proteases by CD4^+^CD20^+^ T cells and CD8^+^CD20^+^ T cells in healthy controls and patients with BP. Flow cytometry analysis revealed that in both healthy controls and patients with BP, CD4^+^CD20^+^ T cells produced higher levels of IL-4, IL-17, and IL-21 compared with CD8^+^CD20^+^ T cells. Conversely, CD8^+^CD20^+^ T cells from both groups secreted greater amounts of IFN-γ, granzyme B, and perforin than CD4^+^CD20^+^ T cells (Supplemental Figure 10, A–L). These data suggest that in BP, CD4^+^CD20^+^ T cells are functionally skewed toward B cell help functions and immune activation, whereas CD8^+^CD20^+^ T cells display a more cytotoxic and pro-inflammatory profile.

To explore whether CD4^+^CD20^+^ T cells exhibit Tfh-like features, we further assessed the expression of CXCR5 and programmed cell death 1 (PD-1), 2 canonical markers associated with Tfh cells. Flow cytometry analysis revealed a greater proportion of CXCR5^+^PD-1^+^ cells among CD4^+^CD20^+^ T cells than among CD4^+^CD20^–^ T cells (Figure 4E and Supplemental Figure 11, A and B). Notably, IL-21, a key effector cytokine involved in Tfh-mediated B cell help (23), was expressed at higher levels in CD4^+^CD20^+^ T cells from patients with BP than in CD4^+^CD20^–^ T cells (Figure 4F and Supplemental Figure 11C). Consistent with this phenotype, BCL6 and MAF, 2 transcription factors associated with Tfh-like differentiation, showed higher expression in CD4^+^CD20^+^ T cells than in CD4^+^CD20^–^ T cells in both patients with BP and healthy controls (Supplemental Figure 11, D–G). Finally, immunofluorescence analysis demonstrated colocalization of CD4, CD20, and CXCR5 in BP skin lesions (Supplemental Figure 12), providing in situ evidence that these cells exhibit Tfh-like features within the lesional microenvironment. Together, these data indicate that CD4^+^CD20^+^ T cells in BP display a pronounced Tfh-like phenotype characterized by expression of CXCR5, PD-1, BCL6, MAF, and IL-21, supporting their potential role in promoting B cell activation and autoantibody production.

### CD4^+^CD20^+^ T cells are involved in B cell differentiation and autoantibody production.

**Figure 5:**
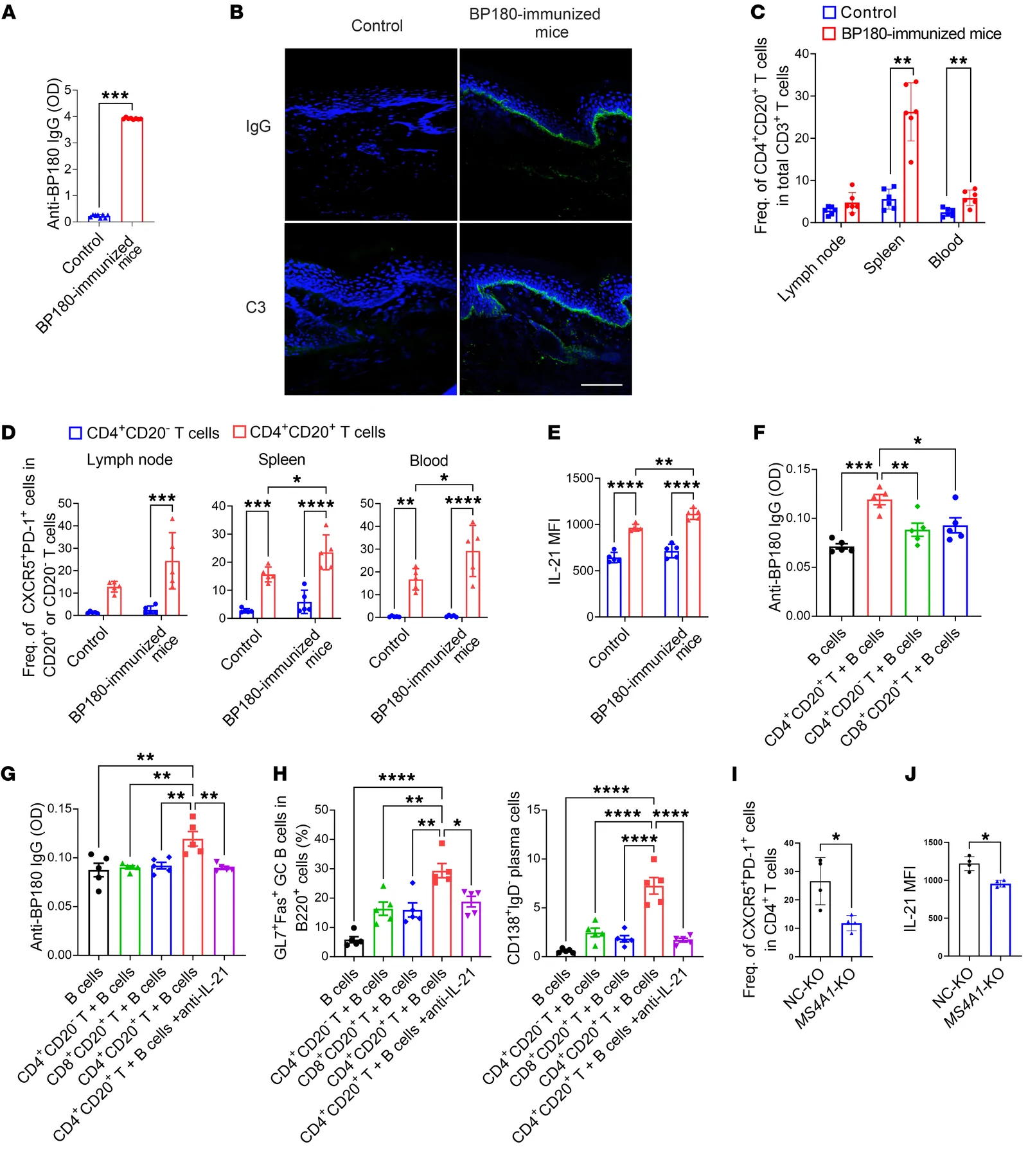
CD20^+^ T cells are increased in BP180-immunized mice and promote B cell differentiation and antibody production. (**A**) Serum anti-BP180 antibody titers in control and BP180-immunized mice at week 4, as measured by ELISA (*n* = 8). (**B**) Representative direct immunofluorescence images showing IgG and C3 deposition (green) in back skin sections. Scale bar = 100 μm. (**C**) Frequencies of CD4^+^CD20^+^ T cells in the spleen, lymph nodes, and peripheral blood of control and BP180-immunized mice (*n* = 6). (**D**) Frequencies of CXCR5^+^PD-1^+^ cells among CD4^+^CD20^+^ and CD4^+^CD20^–^ T cells in the spleen, lymph nodes, and peripheral blood of control and BP180-immunized mice (*n* = 5). (**E**) MFI of IL-21 in CD4^+^CD20^+^ or CD4^+^CD20^–^ T cells in the spleen of control and BP180-immunized mice (*n* = 5). (**F**) Anti-BP180 antibody titers in cell culture supernatants from each group were measured by ELISA (*n* = 5). (**G**) Serum anti-BP180 antibody titers in each group of Rag1^–/–^ mice that received adoptive transfer of B cells along with T cell subsets (or B cells alone as control), measured by ELISA (*n* = 5). (**H**) Frequencies of GL7^+^Fas^+^ germinal center (GC) B cells among B220^+^ B cells and IgD^–^CD138^+^ plasma cells in Rag1^–/–^ recipient mice (*n* = 5). (**I**) Frequencies of CXCR5^+^PD-1^+^ T cells within CD4^+^ T cells from negative control–knockout (NC-KO) and *MS4A1*-knockout (*MS4A1-*KO) T cells (*n* = 4). (**J**) MFI of IL-21 in CD4^+^ T cells from control and *MS4A1-*KO T cells (*n* = 4). All data are presented as the mean ± SD. An unpaired 2-tailed Student’s *t* test was used for **A**, **C**, **I**, and **J**; 1-way ANOVA with Tukey’s post hoc test was used for **F**–**H**; and 2-way ANOVA with Tukey’s post hoc test was used for **D** and **E**. *P < 0.05, **P < 0.01, ***P < 0.001, ****P < 0.0001.

To further investigate the role of CD20^+^ T cells in the development of key features associated with BP, we established an active immunization model by immunizing SJL/J mice with a recombinant fragment of murine BP180 (24). Following immunization, all SJL/J mice developed BP180-specific IgG antibodies, which peaked at 4 weeks postimmunization (Figure 5A). Immunofluorescence microscopy confirmed the binding of circulating IgG and complement component C3 to the basement membrane zone, indicating successful induction of BP-specific humoral autoimmunity in this model (Figure 5B). Flow cytometry analysis showed that the frequency of CD3^+^CD20^+^ T cells was increased in the spleen, lymph nodes, and peripheral blood of BP180-immunized mice compared with control mice (Supplemental Figure 13, A and B). Among total CD3^+^ T cells, the frequency of CD4^+^CD20^+^ T cells was increased in the spleen and peripheral blood of BP180-immunized mice, whereas the frequency of CD8^+^CD20^+^ T cells was not significantly different between the 2 groups (Figure 5C and Supplemental Figure 13, C–E). Furthermore, analysis of the proportions of CD4^+^ and CD8^+^ T cells within the CD3^+^CD20^+^ T cell population revealed that the proportion of CD4^+^CD20^+^ T cells was significantly higher than that of CD8^+^CD20^+^ T cells in both control and BP180-immunized mice (Supplemental Figure 14, A and B).

We next examined whether murine CD4^+^CD20^+^ T cells displayed Tfh-like features similar to those observed in human BP. Compared with CD4^+^CD20^–^ T cells, CD4^+^CD20^+^ T cells from BP180-immunized mice contained a higher proportion of CXCR5^+^PD-1^+^ T cells (Figure 5D and Supplemental Figure 15A). CD4^+^CD20^+^ T cells also produced more IL-21 and expressed higher levels of the transcription factors BCL6 and MAF than CD4^+^CD20^–^ T cells in both control and BP180-immunized mice (Figure 5E and Supplemental Figure 15, B–F). These findings indicate that CD4^+^CD20^+^ T cells in the BP180-immunized mouse model exhibit a Tfh-like phenotype resembling that observed in patients with BP.

To further assess the cellular interactions of CD20^+^ T cells, we analyzed cell-cell communication networks reconstructed from the scRNA-seq dataset. Interactions of CD20^+^ T cells with dendritic cells, monocytes, NK cells, and B cells were markedly enriched relative to those of CD20^–^ T cells, suggesting that CD20^+^ T cells are integrated into an immune network that may amplify pathological responses (Supplemental Figure 16). We next directly tested the B cell helper capacity of distinct T cell subsets. The indicated T cell subsets (CD4^+^CD20^+^, CD4^+^CD20^–^, and CD8^+^CD20^+^ T cells) and B220^+^ B cells were sorted from the spleens and inguinal lymph nodes of BP180-immunized mice and subjected to ex vivo coculture assays. Compared with CD4^+^CD20^–^ T cells and CD8^+^CD20^+^ T cells, CD4^+^CD20^+^ T cells more efficiently promoted B cell differentiation into IgD^–^CD138^+^ plasma cells and GL7^+^Fas^+^ germinal center (GC) B cells, as well as enhanced autoantibody production (Figure 5F and Supplemental Figure 17, A–D).

To determine whether these functional differences were also evident in vivo, we performed adoptive transfer experiments in Rag1^–/–^ mice, which lack mature lymphocytes (Supplemental Figure 18A). ELISA of serum from recipient mice revealed higher levels of anti-BP180 antibodies in those that received CD4^+^CD20^+^ T cells and B cells than in those that received other T cell subsets with B cells or B cells alone (Figure 5G). Consistent with these serological findings, analysis of splenocytes revealed increased frequencies of GL7^+^Fas^+^ GC B cells and IgD^–^CD138^+^ plasma cells in mice receiving CD4^+^CD20^+^ T cells plus B cells compared with the other recipient groups (Figure 5H and Supplemental Figure 18, B and C). Importantly, treatment with an IL-21–neutralizing antibody significantly attenuated the capacity of CD4^+^CD20^+^ T cells to promote B cell differentiation and antibody production (Figure 5, G and H, and Supplemental Figure 18, B and C), indicating that the pathogenic B cell helper activity of this subset depends, at least in part, on IL-21–mediated Tfh-like effector function. Under these transfer conditions, recipient Rag1^–/–^ mice did not develop overt BP-like blistering, indicating that this system models pathogenic B cell help rather than complete disease recapitulation.

Finally, to explore the functional role of CD20 expression, we isolated primary mouse T cells and generated *MS4A1*-KO T cells by lentiviral transduction. The proportions of CD3^+^CD20^+^ and CD4^+^CD20^+^ T cells were reduced in *MS4A1*-KO T cells (Supplemental Figure 19, A–D). In addition, compared with negative control–KO (NC-KO) T cells, *MS4A1*-KO T cells showed reduced proportions of CD4^+^CXCR5^+^PD-1^+^ Tfh cells and decreased intracellular IL-21 production (Figure 5, I and J, and Supplemental Figure 19, E and F). These findings demonstrate that CD4^+^CD20^+^ T cells are expanded in BP180-immunized mice, exhibit Tfh-like characteristics, and actively promote B cell differentiation and pathogenic antibody production.

### Endogenous CD20 expression in human CD20^+^ T cells is regulated by the transcription factor PAX5.

**Figure 6:**
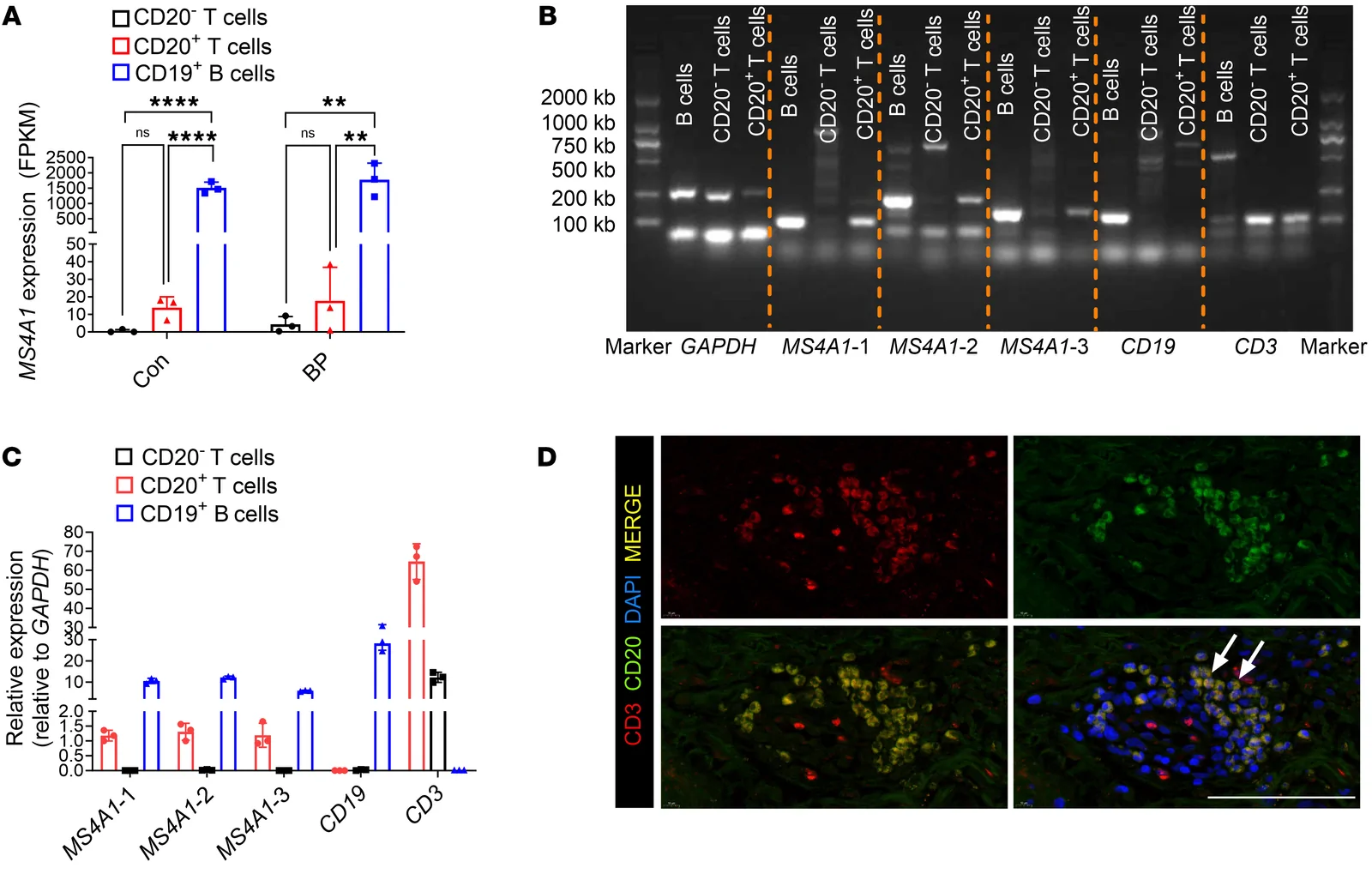
CD20^+^ T cells exhibit endogenous expression of CD20 in human T cells. (**A**) RNA-seq of CD19^+^ B cells, CD3^+^CD20^–^ T cells, and CD3^+^CD20^+^ T cells obtained via FACS to evaluate the CD20-encoding gene *MS4A1* (*n* = 3). FPKM, fragments per kilobase of transcript per million mapped reads. (**B**) Agarose gel electrophoresis of qPCR products for *MS4A1*, *CD3*, and *CD19* using cDNA from sorted CD19^+^ B cells, CD3^+^CD20^–^ T cells, and CD3^+^CD20^+^ T cells. (**C**) qPCR analysis of *MS4A1*, *CD3*, and *CD19* expression in sorted CD19^+^ B cells, CD3^+^CD20^–^ T cells, and CD3^+^CD20^+^ T cells from healthy donor peripheral blood, using 3 independent primer pairs for *MS4A1* (*MS4A1*-1, *MS4A1*-2, *MS4A1*-3) (*n* = 3). (**D**) Representative RNA in situ hybridization images showing CD3 and CD20 expression in the skin tissue sections of patients with BP (*n* = 2). Arrows indicate CD3^+^CD20^+^ T cells. Scale bar = 100 μm. All data are presented as the mean ± SD. One-way ANOVA with Tukey’s post hoc test was used for **A**. **P < 0.01, ****P < 0.0001.

The origin of CD3^+^CD20^+^ T cells remains controversial. Some studies have reported that these cells endogenously express CD20 mRNA (*MS4A1*) (25, 26), whereas others have suggested that CD20 may be acquired from B cells through trogocytosis (4). To address this question, we first examined *MS4A1* expression in our bulk RNA-seq dataset. As expected, CD19^+^ B cells showed high levels of *MS4A1* expression, whereas CD20^+^ T cells expressed low but detectable levels of *MS4A1*, and CD20^–^ T cells showed little to no expression in both healthy controls and patients with BP (Figure 6A). To further determine whether T cells independently express CD20, we isolated CD20^+^ T cells, CD20^–^ T cells, and B cells from healthy donors via FACS. Quantitative real-time PCR analysis with 3 *MS4A1* primer pairs confirmed that CD20^+^ T cells exhibited endogenous expression of *MS4A1*, albeit at lower levels than in B cells (Figure 6, B and C). Furthermore, FISH revealed CD20 signals in a portion of CD3^+^ T cells from the BP skin lesions (Figure 6D). These results indicate that CD20^+^ T cells endogenously express CD20.

**Figure 7:**
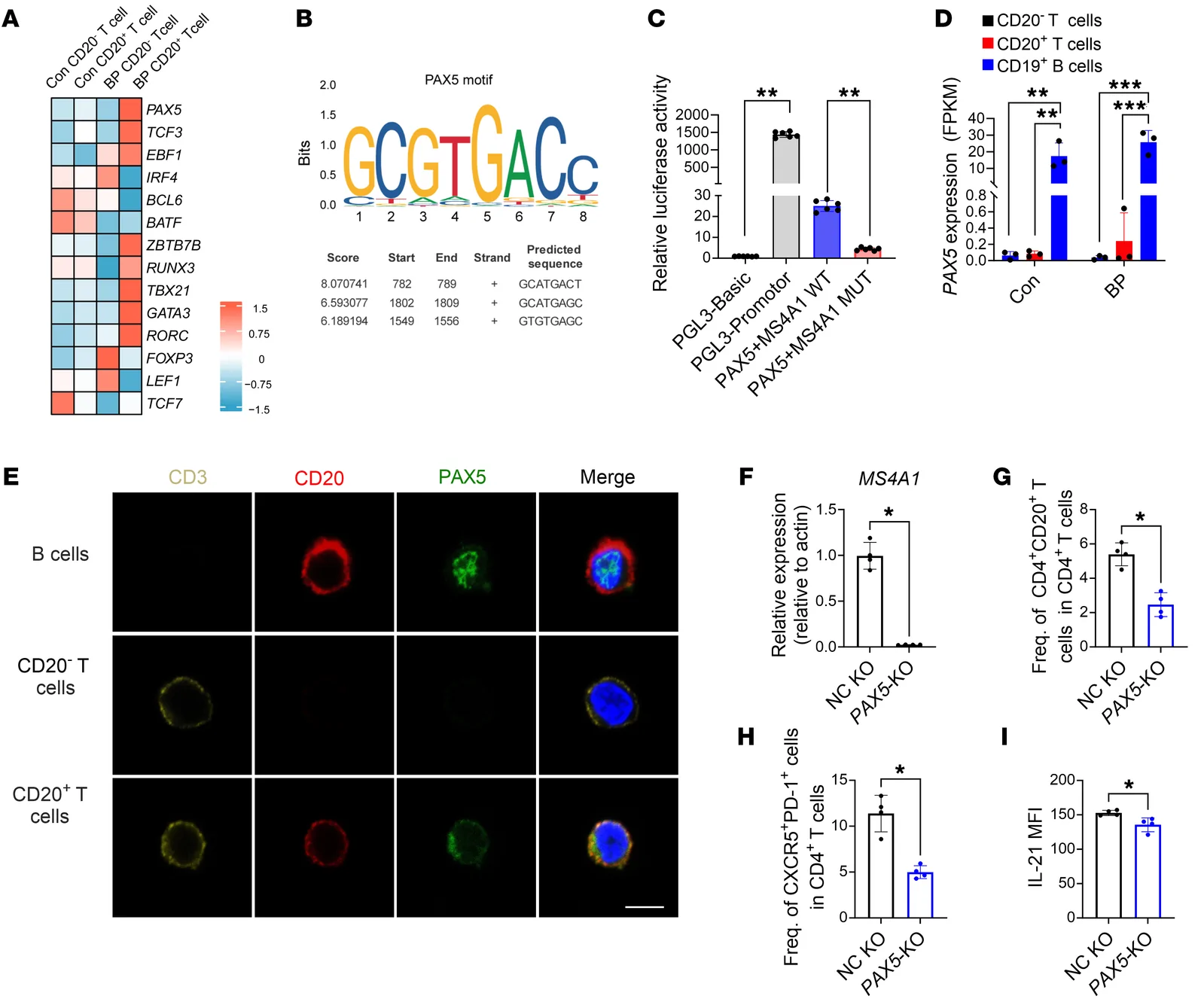
The transcription factor PAX5 regulates endogenous CD20 expression in human CD20^+^ T cells. (**A**) Heatmap showing the top differentially expressed transcription factors between CD3^+^CD20^–^ T cells and CD3^+^CD20^+^ T cells. (**B**) Motifs resembling PAX5-binding motifs within the *MS4A1* regulatory region. (**C**) Luciferase activities of wild-type *MS4A1* and *MS4A1* with a *PAX5*–binding site mutation were determined via luciferase reporter assays in HEK293T cells (*n* = 6). (**D**) Bulk RNA-seq analysis of *PAX5* expression in FACS-sorted CD19^+^ B cells, CD3^+^CD20^–^ T cells, and CD3^+^CD20^+^ T cells from Con and patients with BP (*n* = 3). (**E**) Representative immunofluorescence images showing PAX5 (green), CD3 (red), and CD20 (yellow) in sorted human CD3^+^CD20^+^ T cells, CD3^+^CD20^–^ T cells, and CD19^+^ B cells (*n* = 3). Scale bar = 5 μm. (**F**) *PAX5* mRNA expression in NC-KO or *PAX5*-knockout (*PAX5-*KO) T cells following activated with anti-CD3/CD28 Dynabeads (*n* = 4). (**G**) Frequencies of CD4^+^CD20^+^ T cells among control or *PAX5*-KO T cells (*n* = 4). (**H**) Frequencies of CXCR5^+^PD-1^+^ T cells within CD4^+^ T cells among control or *PAX5*-KO T cells (*n* = 4). (**I**) MFI of IL-21 in CD4^+^ T cells among control or *PAX5*-KO T cells (*n* = 4). All data are presented as the mean ± SD. One-way ANOVA with Tukey’s post hoc test was used for **D**. An unpaired 2-tailed Student’s *t* test was used for **C** and **F**–**I**. *P < 0.05, **P < 0.01, ***P < 0.001.

To identify regulators of CD20 expression in T cells, we next analyzed transcription factors differentially expressed between CD20^+^ T cells and CD20^–^ T cells in the bulk RNA-seq dataset. Among the transcription factors enriched in CD20^+^ T cells, we identified several lineage-associated regulators, including *PAX5*, *TCF3*, and *ZBTB7B* (Figure 7A). We subsequently screened for transcription factors with potential binding affinity for the *MS4A1* promoter region. Using de novo sequence motif searches of sequences within the *MS4A1* promoter region, we identified motifs resembling the consensus binding sequences for the transcription factor PAX5 (Figure 7B). To confirm the role of PAX5 in regulating *MS4A1* expression through its promoter, we conducted a luciferase reporter assay in HEK293T cells. Compared with the wild-type promoter, mutation of the PAX5-binding site significantly reduced luciferase activity, confirming the role of PAX5 in activating *MS4A1* expression (Figure 7C). RNA-seq analysis revealed a slight increase in *PAX5* mRNA levels in CD20^+^ T cells compared with CD20^–^ T cells from patients with BP (Figure 7D). Immunofluorescence analysis revealed that peripheral blood B cells strongly expressed both CD20 and PAX5 whereas CD20^+^ T cells presented lower expression of both proteins, and CD20^–^ T cells did not express PAX5 (Figure 7E). We next asked whether T cell activation could induce the PAX5/CD20 axis. Primary human T cells were isolated and stimulated with anti-CD3/CD28 Dynabeads, and PAX5 expression was monitored over time. Upon stimulation, PAX5 expression increased in a time-dependent manner and peaked at 72 hours after stimulation (Supplemental Figure 20, A and B), consistent with activation-induced engagement of this pathway in T cells.

Although PAX5 is well known for its role in B cell development (27, 28), its association with MS4A1 regulation in T cells has not been previously described to our knowledge. To more directly test the role of PAX5 in regulating CD20 expression in T cells, we generated *PAX5*-KO primary human T cells using CRISPR/Cas9 technology and confirmed efficient knockout by qPCR after lentiviral transduction (Supplemental Figure 21A). Loss of *PAX5* resulted in a marked reduction in *MS4A1* mRNA levels, accompanied by decreased frequencies of total CD20^+^ T cells as well as CD4^+^CD20^+^ and CD8^+^CD20^+^ T cell subsets (Figure 7, F and G, and Supplemental Figure 21, B–E), supporting an important role for PAX5 in maintaining CD20 expression in T cells. In addition, *PAX5* deficiency was associated with reduced frequencies of CD4^+^CXCR5^+^PD-1^+^ T cells and decreased IL-21 and IFN-γ production relative to NC-KO T cells (Figure 7, H and I, and Supplemental Figure 21, G–J), suggesting that PAX5 also contributes to the maintenance of Tfh-like features in this context. Together, these data support a model in which PAX5 contributes to endogenous CD20 expression in T cells and helps sustain aspects of the Tfh-like program associated with CD20^+^ T cells.

### The differentiation of CD20^+^ T cells is regulated by DNA methylation.

**Figure 8:**
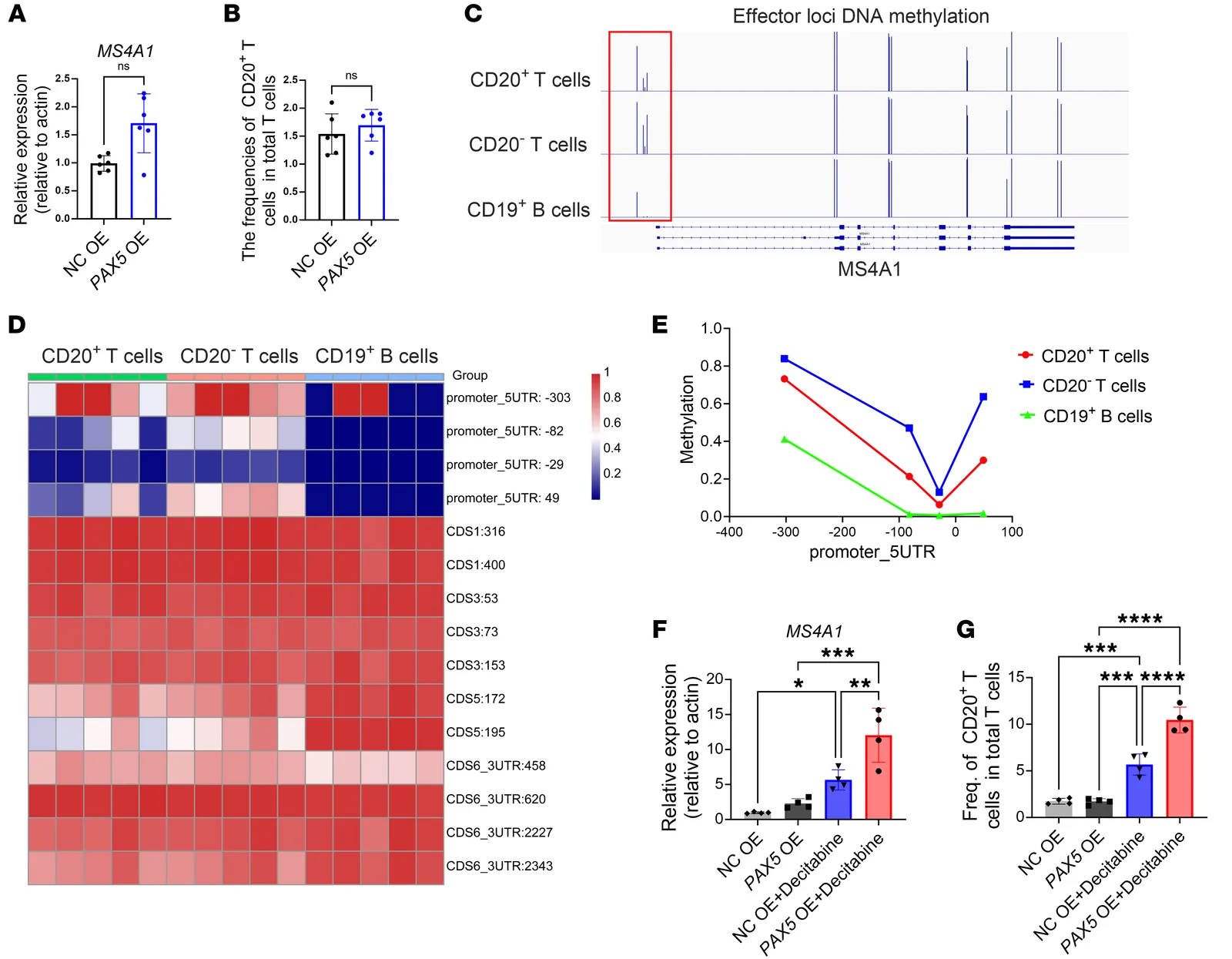
CD20^+^ T cell differentiation is epigenetically regulated by DNA methylation. (**A**) *MS4A1* mRNA expression in NC and *PAX5*-overexpressing T cells (*PAX5* OE) following activation with anti-CD3/CD28 Dynabeads (*n* = 6). (**B**) Frequencies of CD20^+^ T cells in control and *PAX5* OE T cells (*n* = 6). (**C**) CD3^+^CD20^+^ T cells, CD3^+^CD20^–^ T cells and B cells were sorted from 5 healthy controls (*n* = 5). Genome Browser snapshots of the DNA methylation levels of *MS4A1* across gene regions in these cells. (**D**) Heatmap of the CpG methylation status (promoter and exons) at the *MS4A1* locus in the same cell populations as in **C** (*n* = 5). (**E**) DNA methylation levels at individual CpG sites within the *MS4A1* promoter (positions –303, –82, –29, and +49 relative to the transcription start site) in CD3^+^CD20^+^ T cells, CD3^+^CD20^–^ T cells, and B cells. (**F**) *MS4A1* mRNA expression in control and *PAX5* OE T cells treated with or without decitabine (*n* = 4). (**G**) Relative frequencies of CD20^+^ T cells among control and *PAX5* OE T cells treated with or without decitabine (*n* = 4). All data are presented as the mean ± SD. An unpaired 2-tailed Student’s *t* test was used for **A** and **B**; 1-way ANOVA with Tukey’s post hoc test was used for **F** and **G**. *P < 0.05, **P < 0.01, ***P < 0.001, ****P < 0.0001.

We next examined the impact of PAX5 overexpression on T cell differentiation. Notably, overexpressing *PAX5* via lentiviral transduction did not significantly increase *MS4A1* mRNA levels (Figure 8A), although the expression levels of *PAX5* were 5–10 times higher than those in control cells (Supplemental Figure 22A). Similarly, flow cytometry revealed that *PAX5* overexpression did not affect the proportion of CD3^+^CD20^+^ T cells (Figure 8B and Supplemental Figure 22B). Therefore, we hypothesized that epigenetic processes may also regulate CD20^+^ T cell differentiation and function. To investigate whether epigenetic mechanisms contribute to the observed differential expression of CD20, we analyzed DNA methylation patterns in CD20^+^ T cells and CD20^–^ T cells. We isolated CD3^+^CD20^+^ T cells, CD3^+^CD20^–^ T cells, and CD19^+^ B cells from 5 healthy donors and conducted amplicon bisulfite sequencing to analyze DNA methylation levels across the *MS4A1* gene. The results revealed that differences in methylation levels among these cell populations were primarily attributed to differential methylation in the promoter region of *MS4A1* (Figure 8, C and D). Specifically, compared with CD3^+^CD20^+^ T cells, CD3^+^CD20^–^ T cells exhibited higher methylation levels at certain loci within the *MS4A1* promoter, such as –82 and +49 relative to the transcription start site (Figure 8E). Given the altered promoter methylation levels observed in CD20^+^ T cells, we introduced decitabine, a DNA methyltransferase (DNMT) inhibitor, into our coculture system. Our results revealed that DNMT inhibitor treatment increased CD20 expression in T cells overexpressing *PAX5* in vitro (Figure 8, F and G, and Supplemental Figure 22C), suggesting that DNA methylation and PAX5 jointly regulate CD20^+^ T cell development. These findings indicate that DNMT-mediated DNA methylation regulates the differentiation fate of CD20^+^ T cells.

## Discussion

In this study, we identify CD20^+^ T cells as a previously underappreciated component of the adaptive immune response in BP and provide evidence that these cells contribute to pathogenic humoral autoimmunity. We found that CD20^+^ T cells were expanded in both the peripheral blood and skin lesions of patients with BP and that their abundance correlated with serum anti-BP180 autoantibody titers and disease activity. While previous research has revealed correlations between CD20^+^ T cells and autoimmune or viral diseases, their functions, transcriptional profiles, and origins in humans have remained poorly defined. We show that CD20^+^ T cells are enriched for antigen-specific T cells; display increased proliferative, metabolic, and pro-inflammatory activity; and comprise functionally distinct CD4^+^ and CD8^+^ subsets. In particular, CD4^+^CD20^+^ T cells exhibit a pronounced Tfh-like phenotype and promote B cell differentiation and autoantibody production, whereas CD8^+^CD20^+^ T cells display predominantly cytotoxic and pro-inflammatory features. Mechanistically, we further show that *MS4A1* is endogenously expressed in T cells and is regulated by DNA methylation and the transcription factor PAX5. Our study identifies CD20^+^ T cells as an important pathogenic adaptive immune component in BP and suggests that these cells may contribute to disease amplification and humoral autoimmunity.

One important advance of the present study is the demonstration that CD20^+^ T cells are present not only in the circulation but also within BP lesional skin and BF. CD20^+^ T cells have previously been described in the blood and in various tissues such as the thymus, bone marrow, secondary lymphoid organs, cerebrospinal fluid, and brain tissue (3, 22, 26). However, their presence within the tissue microenvironment of autoimmune blistering disease has not been demonstrated previously. The enrichment of these cells in BP lesions suggests that they are actively recruited to sites of inflammation and may participate directly in local immune amplification. In this regard, our data indicate that lesional CD20^+^ T cells in BP are predominantly CD4^+^ T cells, in contrast with the predominance of CD8^+^CD20^+^ T cells described in central nervous system lesions of multiple sclerosis (29). This disease-specific difference reinforces the notion that CD20^+^ T cells are a heterogeneous compartment whose functional polarization is shaped by inflammatory context. In BP, our findings suggest that the lesional environment favors the accumulation of a CD4^+^ Tfh-skewed CD20^+^ subset, rather than a predominantly cytotoxic CD8^+^ population.

T cells are central to autoimmune processes, where the breakdown of self-tolerance can lead to the production of autoantibodies, inflammation, and tissue infiltration by immune cells, ultimately resulting in the onset of autoimmune diseases (30). In this study, we show that CD20^+^ T cells in BP are enriched for autoreactive cells and display heightened effector responsiveness. Using peptide stimulation assays and pMHC II tetramer staining, we found that the CD4^+^CD20^+^ subset was enriched for BP180-NC16A–reactive T cells. In contrast with conventional T cells, CD20^+^ T cells in BP exhibited increased proliferative capacity and pro-inflammatory responses to autoantigens, characterized by increased production of cytokines, including IL-4, IL-17, and IFN-γ, which is consistent with observations in other autoimmune diseases (4, 7, 31). These findings extend previous correlative studies by showing that CD20^+^ T cells in BP are not passive bystanders but rather a highly responsive autoreactive population capable of amplifying both autoimmune and inflammatory responses.

Our additional scRNA-seq analyses, together with complementary flow cytometric phenotyping, show that CD20^+^ T cells are not a uniform population. Instead, CD4^+^CD20^+^ T cells preferentially express molecules associated with B cell help and Tfh-like programming, including PD-1, CXCR5, BCL6, MAF, and IL-21, whereas CD8^+^CD20^+^ T cells are enriched for activation and cytotoxic pathways and express higher levels of IFN-γ, granzyme B, and perforin. This distinction is important because it places the pathogenic relevance of CD20^+^ T cells in BP primarily within the CD4^+^ subset, while acknowledging that the CD8^+^ subset may contribute to tissue inflammation through cytotoxic and pro-inflammatory mechanisms. More broadly, these data support the idea that CD20^+^ T cells exhibit context-dependent functional plasticity across diseases. In multiple sclerosis, CD20^+^ T cells have been reported to display a predominantly Th1/Tc1-like phenotype (22), whereas in rheumatoid arthritis they are enriched for CXCR5^+^PD-1^+^ Tfh and CXCR5^–^PD-1^+^ peripheral helper states (7). Our data indicate that in BP, CD20^+^ T cells are functionally biased toward a Tfh-like B cell helper program, particularly within the CD4^+^ subset.

Although our data support a clear Tfh-like bias in CD4^+^CD20^+^ T cells, we intentionally define this population as Tfh-like rather than canonical Tfh cells. In addition to expressing PD-1, CXCR5, BCL6, MAF, and IL-21, these cells retain features associated with other Th cell programs, including Th1- and Th17-related signatures. Such functional overlap is not unexpected in chronic autoimmune inflammation and is consistent with the concept of hybrid Th cell states in inflamed tissues. Despite this heterogeneity, our data strongly support a dominant B cell helper role for CD4^+^CD20^+^ T cells in BP. These cells, preferentially enriched in BP skin lesions, promoted GC B cell differentiation, plasma cell generation, and autoantibody production more efficiently than CD4^+^CD20^–^ or CD8^+^CD20^+^ T cells in both ex vivo coculture and adoptive transfer assays, and their B cell helper activity was markedly attenuated by IL-21 neutralization in vivo. Together, these findings strengthen the mechanistic link between the Tfh-like phenotype of CD4^+^CD20^+^ T cells and their pathogenic B cell helper function in BP.

Although CD20^+^ T cells were identified in the 1990s, their origin remains contentious. Recent research highlights the role of trogocytosis, the process of transferring intact membrane patches between cells, in modulating immune responses, particularly between T cells and antigen-presenting cells (32). For example, de Bruyn et al. demonstrated that HLA-DR and CD20 are rapidly transferred from B cells to T cells (within 15 minutes) during coculture experiments (31). Additionally, studies using mouse models have shown that T cells can acquire CD20 from CD20-expressing B cells through direct contact, supporting the trogocytosis hypothesis (4). However, CD20^+^ T cells may also express CD20 endogenously. In our study, we found that CD20^+^ T cells isolated from human peripheral blood expressed *MS4A1* mRNA, which is consistent with previous findings that human T cells can transcribe *MS4A1* and synthesize CD20 themselves (25, 26, 33). Additionally, another study reported that CD3^+^CD20^+^ T cells do not express HLA-DR, a molecule typically transferred to T cells during trogocytosis (26). Furthermore, our bulk RNA-seq analysis revealed low or no expression of other membrane proteins associated with CD20, such as HLA-DR, CD40, and CD81, in CD20^+^ T cells, suggesting that CD20 is endogenously expressed rather than acquired via trogocytosis. Studies involving lymph node T cells from monkeys revealed a marked increase in *MS4A1* mRNA expression when these cells were stimulated with mitogens and IL-2 in vitro, suggesting that the cellular microenvironment plays a crucial role in regulating CD20 expression (34). Our results demonstrated that CD20 expression on CD20^+^ T cells is upregulated following TCR activation, independently of the presence of B cells. Thus, our data support the view that at least a subset of CD20^+^ T cells can endogenously express CD20, rather than acquiring it solely through trogocytosis.

PAX5 is a crucial transcription factor involved in lineage commitment and identity, particularly in B cells and T cells (28, 35). Loss of PAX5 can reprogram pro-B cells into hematopoietic progenitors with the potential to differentiate into various cell types, including myeloid cells, macrophages, granulocytes, osteoclasts, NK cells, and T cells (28, 36). These findings underscore the important role of PAX5 in immune cell development. Aberrant expression of PAX5 has been implicated in primary cutaneous T cell lymphomas (37), and experimental models have demonstrated that PAX5 overexpression can induce T cell neoplasms (38). Our study revealed that PAX5 was expressed in CD20^+^ T cells, that the *MS4A1* regulatory region contained a predicted PAX5-binding motif, and mutation of this site reduced reporter activity. In parallel, TCR stimulation induced PAX5 expression, followed by increased CD20 expression, supporting activation-dependent engagement of the PAX5/CD20 axis in T cells. Consistent with this model, *PAX5* deficiency reduced CD20 expression, decreased the frequency of CXCR5^+^PD-1^+^ cells, and lowered IL-21 production. These findings suggest that PAX5 helps sustain the activation state of CD20^+^ T cells and reinforces their Tfh-like features, although it is unlikely to act as the sole determinant of Tfh lineage commitment.

In addition to transcription factors, epigenetic mechanisms are crucial for regulating CD20 expression, particularly in B cells, and involve processes such as histone acetylation and DNA methylation (1). Previous studies have shown that treatment with histone deacetylase inhibitors, such as trichostatin A (39), and DNMT inhibitors, such as 5-aza-2-deoxycytidine (40), can increase CD20 mRNA and protein levels in B cells. In our study, we found that CD20^+^ T cells have lower DNA methylation levels in the *MS4A1* promoter region than do CD20^–^ T cells. These findings suggest that in CD20^+^ T cells, this region has increased accessibility to transcription factors. Interestingly, overexpressing *PAX5* alone did not result in an increase in CD20 expression. However, when *PAX5* overexpression was combined with DNMT inhibitor treatment, CD20 expression in T cells was markedly increased. These findings indicate that DNMT inhibition reduces the methylation of the *MS4A1* promoter, thereby facilitating greater PAX5 binding to the promoter and promoting *MS4A1* transcription and translation. These results highlight the complex interplay between epigenetic modifications and transcription factor regulation in CD20^+^ T cell biology.

Anti-CD20 therapy is commonly used to treat B cell lymphoma and autoimmune disorders. CD20 positivity renders this pathogenic CD20^+^ T cell population susceptible to anti-CD20 therapy. Multiple studies have found that RTX treatment not only efficiently depletes CD20^+^ B cells from the blood but also eliminates the CD20^+^ T cell subset. Both cell types reappear after discontinuation of anti-CD20 antibodies, and CD20^+^ T cells reappear in the peripheral blood earlier than B cells (4). In addition, we found that JAK/STAT signaling was enriched in CD4^+^CD20^+^ T cells and that baricitinib reduced the frequency of CD20^+^ T cells and suppressed Tfh-related effector functions, including IL-21 and IFN-γ production (Supplemental Figure 23). These observations raise the possibility that pathogenic CD20^+^ T cells may be targeted either through CD20-directed depletion or by inhibiting upstream signaling pathways that sustain their activation and effector function.

Although our active immunization model reproduced the major serologic and immunopathologic features of BP, including anti-BP180 autoantibodies and linear IgG/C3 deposition at the dermal-epidermal junction, it did not consistently generate overt blistering and therefore does not fully recapitulate all clinical aspects of human disease. Likewise, adoptive transfer of CD4^+^CD20^+^ T cells together with B cells into Rag1^–/–^ mice enhanced B cell differentiation and antibody production but did not induce overt BP-like disease, indicating that this system is best interpreted as a model of pathogenic B cell help rather than complete disease transfer. In addition, we did not perform T cell–specific conditional deletion of CD20 or PAX5 in vivo, nor did we generate a mouse single-cell transcriptomic atlas to more precisely resolve the heterogeneity and lineage relationships of CD20^+^ T cell subsets in the BP model. These issues will need to be addressed in future studies to define the in vivo functions and therapeutic potential of these cells more precisely.

In conclusion, our study identifies CD20^+^ T cells as a pathogenic adaptive immune component in BP and reveals that CD4^+^CD20^+^ T cells constitute a Tfh-like, antigen-reactive B cell helper subset that promotes B cell differentiation and autoantibody production, whereas CD8^+^CD20^+^ T cells are biased toward cytotoxic and pro-inflammatory functions. We further show that CD20 is endogenously expressed in T cells and is regulated by DNA methylation and PAX5, thereby defining a previously unrecognized regulatory program associated with pathogenic T cell activation in BP. These findings not only expand our understanding of CD20 biology beyond the B cell lineage but also nominate CD20^+^ T cells and their associated signaling pathways as potential therapeutic targets in BP.

## Methods

### Sex as a biological variable.

Female SJL/J mice were used in this study. As previously reported, female SJL/J mice are more susceptible than males to the induction of BP-like skin lesions and phenotypes (24).

### Human samples.

Peripheral blood, BF, and skin tissues were collected from patients with BP at the Department of Dermatology, Xijing Hospital. The study included 36 untreated patients (mean age: 69 years, range: 51–87) and 35 healthy controls (mean age: 60 years, range: 48–69). Healthy controls were frequency-matched for sex and broadly age-matched. The detailed patient characteristics are provided in Supplemental Tables 1 and 2. BP diagnoses were confirmed on the basis of typical clinical and histological presentations, direct or indirect immunofluorescence examinations, and detection of circulating autoantibodies against BP180-NC16A. All enrolled participants developed blisters within 1 month and had not received systemic or topical immunosuppressive treatments before blood sample collection. Ten patients each with pemphigus vulgaris, psoriasis, and lichen planus donated skin lesions for tissue immunofluorescence. Healthy controls were selected to match patients in terms of sex and age, and none exhibited signs of chronic autoimmune diseases or acute infections in their blood samples. PBMCs were isolated from blood samples using human lymphocyte separation medium with Ficoll gradient centrifugation (Dakewei) following the manufacturer’s instructions. BF was collected in an ice bath and immediately centrifuged for 10 minutes at 300*g* to separate the cells from the BF. HLA typing was conducted via Sanger sequencing of the second exon at BGI LifeTech, China.

### Mice.

SJL/J mice (8–10 weeks old) were purchased from Beijing Vital River Laboratory Animal Technology Company. The mice were immunized with a recombinant form of the immunodominant 15th noncollagenous domain of murine BP180 as previously described (24). Briefly, each mouse was injected subcutaneously in the hind footpad with 60 μg of murine BP180 protein (fused with GST; Proteintech Group) emulsified in the nonionic block copolymer adjuvant TiterMax (Sigma-Aldrich, H4397). Boost immunizations were administered twice at 3-week intervals. NC mice were immunized with TiterMax alone. After 4 weeks, biopsies were obtained from nonlesional skin to assess IgG and C3 deposits at the epidermal-dermal junction. Immune cells isolated from blood, lymph nodes, and spleen were stained with fluorochrome-labeled antibodies and analyzed using FlowJo software. Peripheral blood was collected by retro-orbital bleeding under anesthesia.

For the adoptive T cell transfer mouse model, recipient Rag1^–/–^ mice (6–8 weeks old) were obtained from Chengdu Yaokang Biotechnology company. Donor single-cell suspensions were prepared from the spleens and lymph nodes of BP180-immunized mice harvested 4 weeks postimmunization. The tissues were mechanically dissociated through a 70 μm cell strainer (Corning, product number 431751) in RPMI 1640 medium supplemented with 10% FBS, streptomycin (100 μg/mL), penicillin G (100 U/mL), and l-glutamine (0.3 mg/mL). Single cells were subsequently stained with antibodies, followed by sorting of CD4^+^CD20^+^, CD8^+^CD20^+^, and CD4^+^CD20^–^ T cells using a MoFLO XDP (Beckman Coulter) flow cytometer. Magnetically labeled B cells were isolated using a B cell isolation kit (Miltenyi Biotec, 130-090-862). For adoptive transfer, sorted T cells from each subset, along with 1 × 10^6^ B cells, were intravenously injected into the Rag1^–/–^ recipients. In a parallel cohort, a separate group of Rag1^–/–^ mice receiving adoptive transfer of CD4^+^CD20^+^ T cells was concurrently administered an anti–IL-21 neutralizing antibody (100 μg per mouse, Invitrogen, 16-7211-85) via tail vein injection on day 0. Fourteen days after transfer, the skin, spleens, and serum were harvested from recipient mice for subsequent analysis.

### scRNA-seq and data analysis.

ScRNA-seq libraries were constructed using the 10x Genomics Chromium Single Cell 3′ Library & Gel Bead Kit v3.1 according to the manufacturer’s protocol and sequenced on the Illumina NovaSeq 6000 platform. The raw sequencing data were processed using Cell Ranger (v6.1.2, 10x Genomics). The FASTQ files were aligned to the human reference genome GRCh38 (hg38), and gene expression matrices were generated based on unique molecular identifiers (UMIs). Subsequent analyses were conducted using the Seurat package (v5.2.1) in R. Cells with fewer than 200 or more than 4,000 detected genes, total UMI counts exceeding 20,000, mitochondrial gene percentages greater than 10%, or hemoglobin gene percentages greater than 10% were excluded. The filtered data were then normalized using the “LogNormalize” method. Highly variable genes (*n* = 2,000) were identified using the “vst” method, and the data were scaled prior to dimensionality reduction. Principal component analysis was performed on the highly variable genes. For batch correction and data integration across samples, Harmony-based integration was applied using the IntegrateLayers function with the first 20 principal components. The integrated object was joined with JoinLayers, and subsequent dimensionality reduction was conducted via UMAP using the top 15 Harmony components. Cell types were annotated based on the expression of canonical marker genes. CD20^+^ T cells were identified as a subset of T lymphocytes coexpressing canonical T cell markers (e.g., *CD3D*, *CD3E*, *CD4*, and *CD8A*) and *MS4A1*. A threshold for *MS4A1* expression was applied to distinguish CD20^+^ from CD20^–^ T cells, and the resulting CD20^+^ T cell population was extracted from the Seurat object for downstream analyses, including differential gene expression, functional enrichment, and cell-cell communication analyses. To investigate intercellular communication among different cell types, we used the CellChat R package (version 2.1.2). Cell-cell interactions were inferred based on known ligand-receptor pairs. Specifically, the netVisual heatmap function was used to visualize the interaction strengths between CD20^+^ T cells and other immune cell populations.

### Bulk RNA-seq.

Total RNA extracted from the peripheral CD3^+^CD20^+^ T cells, CD3^+^CD20^–^ T cells, and CD19^+^ B cells of patients with BP (*n* = 3) and age- and sex-matched healthy controls (*n* = 3) was subjected to deep sequencing on an Illumina NovaSeq platform (Gene Denovo Biotechnology Corporation). Raw sequencing reads were processed and mapped to the human reference genome, and gene-level raw counts were obtained. Differential gene expression analysis was performed using the DESeq2 R package based on raw count data. Genes with an absolute FC ≥ 1.5 and adjusted *P* value < 0.05 were considered DEGs. For visualization purposes, gene expression levels were normalized as FPKM. Expression profiles and plots were generated using Omicsmart tools. Functional enrichment analyses of DEGs were performed using GO and KEGG databases with the R packages clusterProfiler, org.Hs.eg.db, enrichplot, and ggplot2.

### Tissue and cell immunofluorescence staining.

For the skin tissue samples, 4 μm sections of the paraffin-embedded skin samples were deparaffinized and rehydrated. After being blocked with 5% goat serum for 30 minutes at room temperature, the skin sections were incubated with primary antibodies overnight at 4°C. The following antibodies were used: rat monoclonal CD3 (Abcam, ab11089), rat monoclonal CD4 (Santa Cruz Biotechnology, sc-13573), mouse monoclonal CD20 (Abcam, ab9475), rabbit monoclonal CD20 (Abcam, ab78237), rabbit monoclonal CD19 (Abcam, ab134114), and mouse monoclonal CD8 (Abcam, ab316355). After being washed 3 times with PBS, the skin tissues were incubated with Cy5-conjugated goat anti-rat IgG (Abcam, ab6565), Cy3-conjugated goat anti-mouse IgG (Abcam, ab97035), Cy3-conjugated goat anti-rabbit IgG (Abcam, ab6939), Alexa Fluor 488–conjugated goat anti-mouse IgG (Abcam, ab150113) or Alexa Fluor 488–conjugated goat anti-rabbit IgG (Abcam, ab150077) as secondary antibodies. Nuclear DNA was detected by incubating the sections with Hoechst 33258 (Solarbio Technology, C0021) for 10 minutes at room temperature. Images were acquired using an LSM 880 confocal microscope (Zeiss Optotechnik GmbH).

For cell immunofluorescence staining, CD3^+^CD20^+^ T cells, CD3^+^CD20^–^ T cells and CD19^+^ B cells were isolated from healthy controls via flow cytometry. The suspended cells were seeded on poly-l-lysine–coated coverslips, fixed immediately with 4% paraformaldehyde for 15 minutes, and permeabilized with 0.25% Triton X-100 (Beyotime, P0096) at room temperature. The cells were blocked with 5% goat serum for 30 minutes and then incubated with primary antibodies, including rat monoclonal CD3 (Abcam, ab11089), mouse monoclonal CD20 (Abcam, ab9475), and rabbit monoclonal PAX5 (Abcam, ab109443) antibodies at 4°C overnight. After incubation with the corresponding secondary antibodies and Hoechst 33258, the cells were observed with a confocal microscope (LSM880, Carl Zeiss).

### Flow cytometry analysis.

PBMCs were incubated in FcR blocking reagent (Miltenyi Biotec, 130-059-091) to prevent nonspecific antibody binding and subsequently stained for 30 minutes at 4°C with fluorophore-conjugated antibodies. The following antibodies were used: APC anti-human CD3 antibody (BioLegend, 300312), PE/Cyanine7 anti-human CD4 antibody (BioLegend, 357410), BV605 anti-human CD4 antibody (BioLegend, 317438), PerCP/Cyanine5.5 anti-human CD8 antibody (BioLegend, 344710), FITC anti-human CD8 antibody (BioLegend, 344704), FITC anti-human CD19 antibody (BioLegend, 363008), PE anti-human CD20 antibody (BioLegend, 302306), PE/Cyanine7 anti-human CD20 antibody (BioLegend, 302312), PE anti-human CXCR5 antibody (BioLegend, 356904), PerCP/Cyanine5.5 anti-human CXCR5 antibody (BioLegend, 356910), Pacific Blue anti-human PD-1 antibody (BioLegend, 329916), PerCP/Cyanine5.5 anti-PAX5 antibody (BioLegend, 649710), PE anti-human IFN-γ antibody (BioLegend, 506507), FITC anti-human IL-4 antibody (BioLegend, 500806), Brilliant Violet 605 anti-human IL-17A antibody (BioLegend, 512326), PerCP/Cyanine5.5 anti-human IL-21 antibody (BioLegend, 513012), PE anti-human Perforin Antibody (BioLegend, 308106), and FITC anti-human/mouse Granzyme B Antibody (BioLegend, 515403).

Murine immune cells were stained with APC anti-mouse CD3 antibody (BioLegend, 100236), PE/Cyanine7 anti-mouse CD4 antibody (BioLegend, 100422), BV421 anti-mouse CD4 antibody (BioLegend, 100438), PerCP/Cyanine5.5 anti-mouse CD8a antibody (BioLegend, 100734), BV605 anti-mouse CD8a antibody (BioLegend, 100744), FITC anti-mouse CD19 antibody (BioLegend, 115506), PE anti-mouse CD20 antibody (BioLegend, 150410), Alexa Fluor 488 anti-mouse B220 antibody (BioLegend, 103227), Pacific Blue anti-mouse GL7 antibody (BioLegend, 144614), APC anti-mouse Fas antibody (BioLegend, 152604), PE/Cyanine7 anti-mouse PD-1 antibody (BioLegend, 135216), BV605 anti-mouse CXCR5 antibody (BioLegend, 145513), AF488 anti-mouse IL-21 antibody (R&D Systems, IC594G), PE/Cyanine7 anti-mouse IgD antibody (BioLegend, 405720), APC/Cyanine7 anti-mouse CD138 (Syndecan-1) antibody (BioLegend, 142530), and Zombie UV dye (BioLegend, 423102) in FACS buffer for 30 minutes and then analyzed by flow cytometry (BD LSRFortessa Cell Analyzer). Isotype-matched controls were used to correct for nonspecific antibody binding and spectral overlap, where appropriate. For cytokines, cells were stimulated with PMA and ionomycin with the protein transport inhibitor GolgiPlug (BD Biosciences, 550583) at 37**°**C and 5% CO_2_ for 6 hours. For intracellular staining, the cells were fixed and permeabilized using the Cytofix/Cytoperm Fixation/Permeabilization Solution Kit (BD Biosciences, 554714) or Foxp3/Transcription Factor Staining Buffer Set (eBioscience, 00-5523-00) and stained with fluorescent antibodies for an additional 30 minutes at 4°C in the dark. Data analysis was performed using FlowJo software.

### Tetramer flow cytometry.

PBMCs were isolated from patients with BP and healthy controls, and then, the cells (2 × 10^5^ cells per well) were seeded into 96-well, U-bottom plates and cultured in RPMI 1640 medium (Gibco) supplemented with 10% FBS and streptomycin (100 μg/mL), penicillin G (100 U/mL), and l-glutamine (0.3 mg/mL). PBMCs were stimulated in duplicate using peptide pools (1 μM/well for each peptide) and 20 U/mL recombinant human IL-2 (R&D Systems) at 37°C with 5% CO_2_ for 5 days. Anti-CD3/CD28 Dynabeads (Gibco, 11141D) were used as positive controls to monitor T cell expansion. For a negative control, unstimulated cells were included on each plate.

A PE-labeled ProT2 MHC Tetramer (HLA-DRA1*01:01/DRB1*15:01 ELERIRRSILPYGDS) was constructed by PROIMMUNE. For MHC tetramer staining, 2 μL of tetramer was added to each tube and incubated at room temperature for 1 hour, followed by 1 wash in 15 mL of cold sorting buffer (PBS with 2% FBS and 0.1% NaN_3_) and centrifugation at 300*g* for 5 minutes. For antibody staining, enriched tetramer-binding PBMCs were incubated for 30 minutes at 4°C with APC-conjugated CD3 antibody, BV605-conjugated CD4 antibody, and PE-Cy7–conjugated CD20 antibody against surface markers. The cells were washed and fixed in 2% paraformaldehyde in PBS overnight at 4°C. Single-stained compensation controls were used for spectral compensation. Fluorescence-minus-one controls and/or isotype controls were used where appropriate for gate setting. Tetramer-positive T cells were identified based on their binding to the pMHC II tetramer. The samples were analyzed with a BD LSRFortessa flow cytometer.

### Ex vivo coculture assay.

On day 28 following BP180 immunization, CD4^+^CD20^+^ T cells, CD4^+^CD20^–^ T cells, and CD8^+^CD20^+^ T cells were isolated from the mouse spleen and lymph nodes via FACS. Magnetically labeled B cells were isolated using a B cell isolation kit (Miltenyi Biotec, 130-090-862). Subsequently, each of the 3 T cell subsets was cocultured with B cells at a 1:1 ratio in media supplemented with anti-CD3/CD28 Dynabeads and IL-2. T cell subsets were seeded at a density of 1 × 10^5^ cells per well in a 96-well plate. Following 7 days of incubation, cells were harvested for the analysis of B cell differentiation, and culture supernatants were collected for the measurement of antibody titers by ELISA.

### Cell culture and lentiviral transduction.

For PAX5 overexpression, lentiviral constructs carrying the PAX5 gene or a negative control sequence were generated and packaged by Tsingke Biotechnology. Human primary T cells were extracted from PBMCs using a pan-T isolation kit (Miltenyi Biotec, 130-096-535). First, T cells (2.0 × 10^5^ per well) were activated with anti-CD3/CD28 Dynabeads at a 1:1 ratio in 500 μL of RPMI 1640 medium supplemented with 10% FBS and human rIL-2 (30 IU/mL) in 48-well plates. After 24 hours, 2 × 10^7^ TU of ZsGreen-Puro/CMV-PAX5 or negative control lentivirus in complete RPMI 1640 supplemented with rIL-2 and 8 μg/mL polybrene were added to the cell suspension at a final volume of 750 μL at a multiplicity of infection of 100 for 12 hours. Then, the cells were harvested and incubated in RPMI 1640 culture medium supplemented with 10% FBS, antibiotics, rhIL-2, and anti-CD3/CD28 Dynabeads in 48-well plates for 72 hours. To inhibit promoter methylation in T cells, a 10 μM concentration of the DNMT inhibitor decitabine (MedChemExpress, HY-A0004) was used to pretreat the culture system. After culture, the cells were expanded, and the expression of PAX5 and MS4A1 was evaluated via RT-qPCR.

*PAX5* knockout in primary human T cells and *MS4A1* knockout in primary mouse T cells were performed via the CRISPR/Cas9 system. The cutting efficiency of each CRISPR guide RNA construct was validated by transfecting HEK293T cells (Fuheng Biology) and sequencing the target regions in the genome. Recombinant *PAX5*-KO lentiviruses and *MS4A1*-KO lentiviruses were synthesized by Tsingke Biotechnology and transduced into primary human or mouse T cells as described above.

### Statistics.

Each experiment was performed at least 3 times. All the data are presented as the mean ± SD. Statistical analyses of the data were performed using GraphPad Prism (version 9.0.2) and R. Two-tailed paired and unpaired Student’s *t* tests and 1-way and 2-way ANOVA were used to evaluate the statistical significance of differences among groups. Correlation analysis was performed via Spearman’s rank correlation test. *P* < 0.05 was considered to indicate statistical significance.

### Study approval.

All procedures involving the use of human samples were performed in accordance with institutional guidelines and with approval from the Ethical Committee of Xijing Hospital of the Fourth Military Medical University. Written informed consent was obtained from all participants prior to sample collection. All animal experiments were performed according to institutional guidelines and were approved by the Review Committee for the Use of Animals of the Fourth Military Medical University.

### Data availability.

The accession numbers for the sequencing raw data in this paper are GSA (Genome Sequence Archive in BIG Data Center, Beijing Institute of Genomics, Chinese Academy of Sciences): HRA008361 and HRA018190. Values for all data points in graphs are reported in the Supporting Data Values file.

Additional materials and methods, including primer sequences (Supplemental Table 3), peptide sequences (Supplemental Table 4), and methylation-specific PCR primers (Supplemental Table 5), are provided in the online Supplemental Methods.

## Author contributions

HF, S Shen, S Shao, ED, and GW conceived the project and designed the experiments. HF, S Shen, KL, TC, BW, HM, KX, LL, XL, and YB performed the experiments and data analyses. PQ, JZ, CZ, CX, and BP provided technical assistance. GW, HF, S Shao, and H Qu obtained the funding for this study. HF and S Shen wrote the manuscript with input and revision from GW, ED, S Shao, MF, and H Qiao. All the authors read and approved the final manuscript.

## Conflict of interest

The authors have declared that no conflict of interest exists.

## Funding support

- The National Key R&D Program of China (2022YFC3601800 to GW).
- The National Natural Science Foundation of China (82530098 to GW; 82173410 to HF; 82322057 to S Shao).
- The Natural Science Foundation of Shaanxi Province, China (2024JC-YBQN-0772 to H Qu).

## Supplementary Material

Supplemental data

Unedited blot and gel images

Supporting data values

## Version 1 07/07/2026

In-Press Preview

## Version 2 09/01/2026

Electronic publication
