# Supplementary material for: CD20+ T follicular helper–like cells drive antigen-specific autoimmunity in bullous pemphigoid
Source: J Clin Invest. 2026 Jul 7;136(17):e197665. doi: 10.1172/JCI197665 (PMC13528933; doi:10.1172/JCI197665)
Supplement: Supplementary file 1 [file jci-136-197665-s229.pdf]

This file includes:

Supplemental Materials and Methods

Supplemental Figure 1. Expression of $CD20^+$ T cells and their subsets in the peripheral blood of BP patients.

Supplemental Figure 2. Single-cell RNA sequencing analysis of immune cell subsets in peripheral blood from BP patients and healthy controls.

Supplemental Figure 3. $CD20^+$ T cells in blister fluid from BP patients and other autoimmune and inflammatory skin diseases.

Supplemental Figure 4. Quantification of $CD3^+CD20^+$ and $CD3^+CD20^-$ T cells in BP lesions.

Supplemental Figure 5. Transcriptome characteristics of $CD20^+$ T cells.

Supplemental Figure 6. Analysis of proliferation and intracellular cytokine expression in PBMCs with or without peptide stimulation.

Supplemental Figure 7. TCR activation promotes CD20 expression on T cells.

Supplemental Figure 8. Analysis of tetramer-positive $CD4^+$ T cells in PBMCs with or without peptide stimulation.

Supplemental Figure 9. GSEA enrichment plots for $CD8^+CD20^+$ T cells.

Supplemental Figure 10. Expression of intracellular cytokines in $CD4^+CD20^+$ T cells and $CD8^+CD20^+$ T cells from control and BP patients.

Supplemental Figure 11. Flow cytometry analysis of $CXCR5^+PD\text{-}1^+$ T cells, and IL-21, MAF, and BCL6 expression in peripheral blood $CD4^+CD20^+$ versus $CD4^+CD20^-$ T cell subsets from control and BP patients.

Supplemental Figure 12. Detection of $CD4^+CXCR5^+CD20^+$ T cells in BP patient skin lesions by immunofluorescence.

Supplemental Figure 13. Frequencies of $CD3^+CD20^+$ T cells and their subsets in control and BP180-immunized mice.

Supplemental Figure 14. Frequencies of $CD4^+$ or $CD8^+$ subsets within $CD3^+CD20^+$ T cells in control and BP180-immunized mice.

Supplemental Figure 15. Frequencies of $CXCR5^+PD\text{-}1^+$ T cells and expression of IL-21, BCL6 and MAF in $CD4^+CD20^+$ versus $CD4^+CD20^-$ T cells from control and BP180-immunized mice.

Supplemental Figure 16. Single-cell sequencing analysis of the interaction between $CD20^+$ T cells and other immune cells in human peripheral blood.

Supplemental Figure 17. $CD4^+CD20^+$ T cells promote B cell differentiation and antibody production ex vivo.

Supplemental Figure 18. Flow cytometry analysis of $GL7^+Fas^+$ GC B cells and IgD^-CD138^+ plasma cells in the spleens of recipient $Rag1^{-/-}$ mice after adoptive transfer.

Supplemental Figure 19. The effect of *MS4A1* knockout on T cell subsets and function.

Supplemental Figure 20. TCR activation upregulates PAX5 expression in $CD3^+$ T cells.

Supplemental Figure 21. *PAX5* knockout in T cells reduces the proportion of $CD20^+$ T cells and suppresses Tfh-related functions.

Supplemental Figure 22. DNA methylation and PAX5 govern $CD20^+$ T cell differentiation.

Supplemental Figure 23. Inhibition of the JAK-STAT signaling pathway attenuates the frequencies of $CD20^+$ T cells.

Supplemental Table 1. Demographic characteristics of patients with BP and healthy controls.

Supplemental Table 2. Patient characteristics.

Supplemental Table 3. Sequences of primers for real-time PCR.

Supplemental Table 4. Sequence of overlapping peptides spanning the entire extracellular NC16A region of BP180 (490 – 566).

Supplemental Table 5. Bisulfite sequencing PCR primer sequences for methylation analysis.

Supplemental Materials and Methods

RNA extraction, real-time PCR, and gel electrophoresis

Total RNA was isolated via TRIzol (Invitrogen, Cat# 15596018CN) and subsequent chloroform/isopropanol/ethanol purification. The concentrations and purities of the extracted RNA were determined using a spectrophotometer (Thermo Fisher Scientific, N12391). cDNA was synthesized with a PrimeScript RT reagent Kit (TaKaRa, Cat# RR036A-1) according to the manufacturer's protocols and used as the template for quantitative PCR. Real-time PCR was performed using SYBR Premix Ex Taq II SYBR Green Master Mix (TaKaRa, Cat# RR820A) in 384-well plates according to the manufacturer's instructions. The sizes of the PCR products were analyzed by gel electrophoresis on a 2% agarose gel containing GelRed to stain nucleic acids. The relative quantitative value for each gene was determined via the $2^{-\Delta\Delta CT}$ method. The primers used are listed in Supplemental Table 3. The samples were analyzed in triplicate and considered valid when Ct < 35 and SD Ct < 0.5.

Peptide synthesis

A series of 15-mer peptides, which overlap by 12 amino acids and span the entire sequence of BP180-NC16A, were synthesized by APeptides, China (Supplemental Table 4). Peptides were purified via high-performance liquid chromatography (>95% purity) and validated via mass spectrometry. The synthesized peptides were dissolved in DMSO at 10 mg/mL and stored at -80°C.

T cell proliferation assay

PBMCs were isolated and labeled with carboxyfluorescein succinimidyl ester (Invitrogen, Cat# C34554) at 37°C for 15 minutes. The labeling reaction was stopped by adding fetal bovine serum, after which the cells were counted and seeded into a 96-well U-bottom plate at a density of 2×10^5 cells per well. PBMCs were cultured in the presence of BP180-NC16A peptides at a final concentration of 4 μg/well along with 30 U/mL rhIL-2. As a positive control, anti-CD3/CD28 Dynabeads were added to the

culture medium at a bead-to-cell ratio of 1:1. After 7 days, cells were harvested and stained with anti-CD3 and anti-CD20 antibodies for 30 minutes at 4°C. The proliferation of $CD20^+$ T cells and $CD20^-$ T cells was assessed by flow cytometry.

Fluorescence *in situ* hybridization (FISH)

For FISH experiments, Cy5-labeled probes for human *CD3E* and 6-FAM-labeled probes for human *MS4A1* (Tsingke Biotech) were utilized following the manufacturer's protocol. Thin sections (4-μm thick) of paraffin-embedded samples were deparaffinized in xylene and subsequently rehydrated through a series of ethanol dilutions from 100% to 25%. The slides were treated with diethyl pyrocarbonate-treated water and subjected to proteinase K digestion (40 μg/mL). Following prehybridization in hybridization solution at 37°C for 2 hours, a Cy5-labeled *CD3E* probe and a 6-FAM-labeled *MS4A1* probe (1:40 dilution in 100 μL of hybridization solution) were added and allowed to hybridize for 16 hours at 37°C. After being washed with decreasing concentrations of SSC buffer (from 5× to 2×) at the same temperature used for hybridization, the samples were stained with DAPI (Beyotime, Cat# C1006). Images were captured using an immunofluorescence microscope (Nikon Eclipse, TI-SR).

Enzyme-linked immunosorbent assay (ELISA)

Commercially available ELISA kits (MBL, 7695E) were used to detect anti-BP180 antibodies in patient serum according to the manufacturer's instructions. For detection of circulating BP180-specific IgG in mouse serum, 96-well plates were coated overnight at 4°C with recombinant BP180 (fused with His, Proteintech) at a concentration of 250 ng/well. After blocking with 5% BSA, wells were incubated with diluted mouse serum (1:100-1:100,000,000) for 2 hours, followed by incubation with horseradish peroxidase-conjugated goat anti-mouse antibody (1:10,000 dilution, CWBIO, Cat# CW0102S) for 1 hour at room temperature. After a final wash step, substrate solution was added and the absorbance was read at 450 nm. OD values were normalized to those of negative control mouse sera.

Luciferase reporter assays

For the luciferase reporter assay of the *MS4A1* promoter region, HEK293T cells were co-transfected with a luciferase reporter containing the sequence of the *MS4A1* promoter region and the empty vector or the *PAX5* plasmid (Tsingke, China). The promoter region sequences were synthesized and cloned and inserted into the pGL3-basic luciferase reporter vector (Promega). Luciferase activity was measured with the Dual Luciferase Reporter Assay System (Promega) and normalized to Renilla luciferase activity.

Bisulfite sequencing of the MS4A1 promoter region

Following sorting of $CD3^+CD20^+$ T cells, $CD3^+CD20^-$ T cells, and $CD19^+$ B cells from peripheral blood, DNA extraction was performed using an Omega DNA extraction kit according to the manufacturer's instructions. Bisulfite sequencing of target genes was conducted by E-Gene Co., Ltd. (Shenzhen, China). The primers (Supplemental Table 5) were designed to target regions without CpG sites to prevent amplification bias between methylated and unmethylated sequences. Bisulfite sequencing PCR validation experiments were performed as follows: 500 ng of genomic DNA was converted using the ZYMO EZ DNA Methylation-Gold Kit™ (Zymo Research, D5006), followed by purification of the converted products. PCR amplification was performed in a final reaction volume of 50 µL. After amplification, the PCR products were used for library construction, and the final libraries were assessed for quantity via an Agilent 2100 Bioanalyzer (Agilent Technologies) and real-time PCR. Sequencing was conducted on the Illumina HiSeq platform.

Supplemental Figures

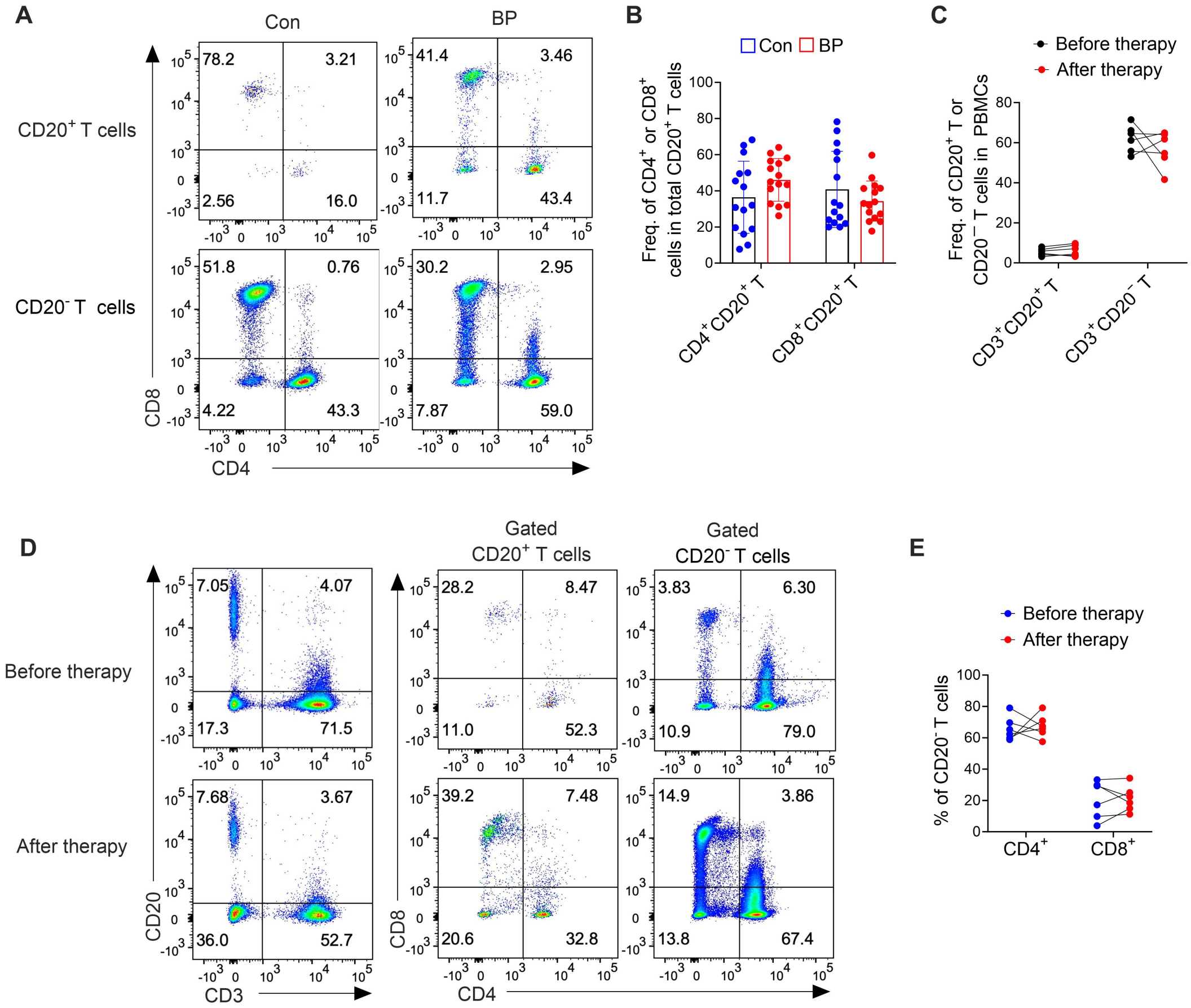

Supplemental Figure 1. Expression of $CD20^+$ T cells and their subsets in the peripheral blood of BP patients. (A) Representative flow cytometry plots showing $CD4^+CD20^+$ or $CD8^+CD20^+$ T cell subsets, and **(B)** frequencies of these subsets in PBMCs of healthy controls (Con) and patients with BP (n = 15). **(C)** Frequencies of $CD3^+CD20^+$ and $CD3^+CD20^-$ T cells in BP patients before and after 9–12 months glucocorticoids treatment (n = 6). **(D)** Representative flow cytometry plots showing total $CD3^+CD20^+$ T cells and $CD20^+$ and $CD20^-$ T cell subsets before and after glucocorticoid treatment. **(E)** Frequencies of $CD4^+$ and $CD8^+$ T cells within $CD20^-$ T cells before and after glucocorticoids treatment in BP patients. All data are presented as the mean ± SD. An unpaired 2-tailed Student's *t* test was used for B; Paired Student's t-test was used for C and E.

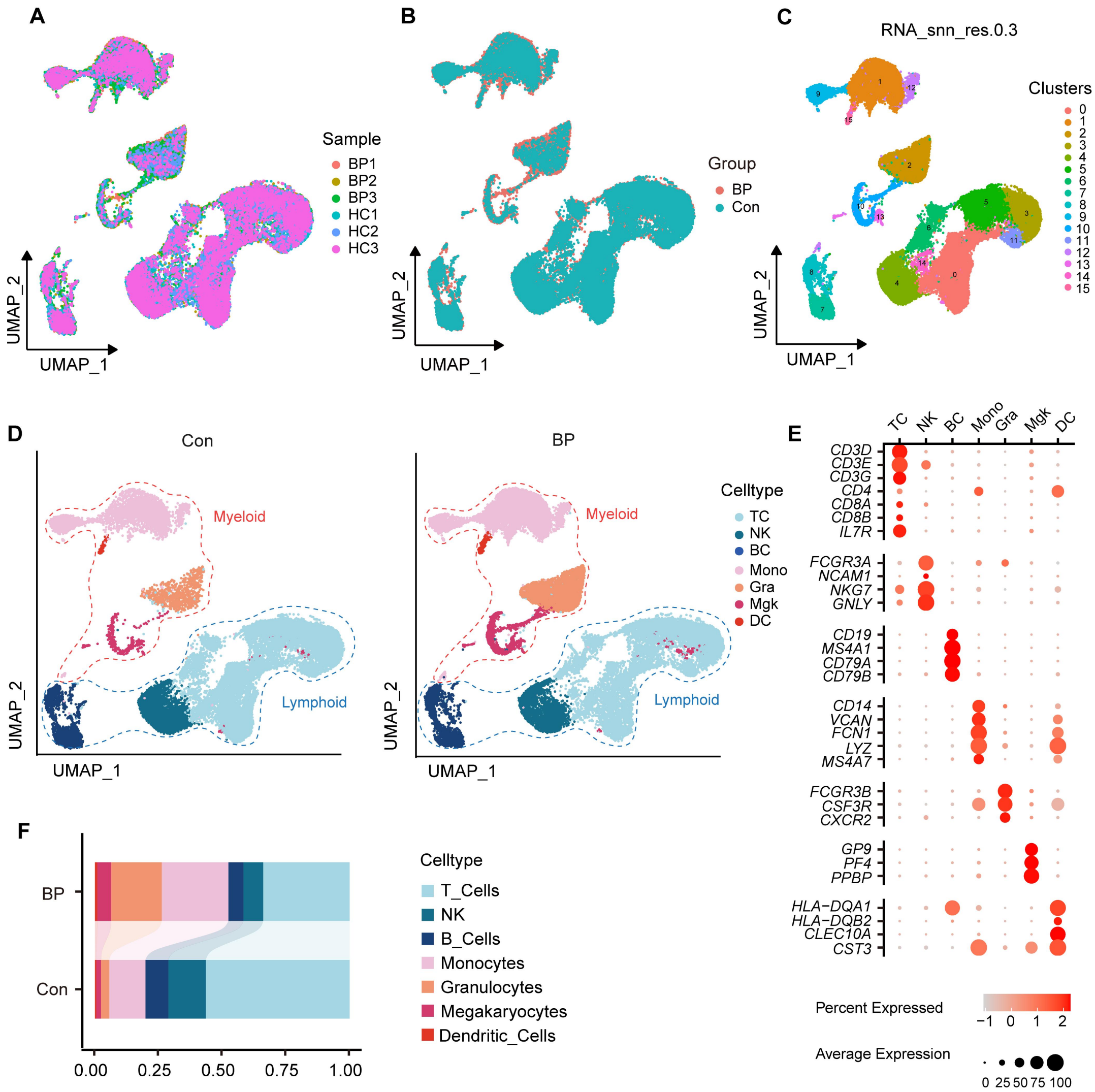

Supplemental Figure 2. Single-cell RNA sequencing analysis of immune cell subsets in peripheral blood from BP patients and healthy controls. (A) Uniform Manifold Approximation and Projection (UMAP) visualization of PBMCs from all individual samples (n = 3 per group). Each dot represents a single cell, colored according to its sample of origin. **(B)** UMAP plot showing the distribution of PBMCs colored by group (BP *vs.* Con). **(C)** UMAP plot showing the clustering of PBMCs from BP patients and healthy controls. **(D)** UMAP plots showing the major annotated immune cell types in Con (left) and BP (right) groups. Each dot represents a single cell, colored according to its assigned cell type: TC (T cells), NK (natural killer cells), BC (B cells), Mono (monocytes), Gra (granulocytes), Mgk (megakaryocytes), and DC (dendritic cells). **(E)** Dot plots showing the expression of canonical marker genes used to define major immune cell types. Columns represent cell types and rows represent marker genes. Dot size indicates the percentage of cells expressing the gene, and color intensity indicates the average expression level. **(F)** Stacked bar plot showing the proportions of different immune cell types between BP patients and controls.

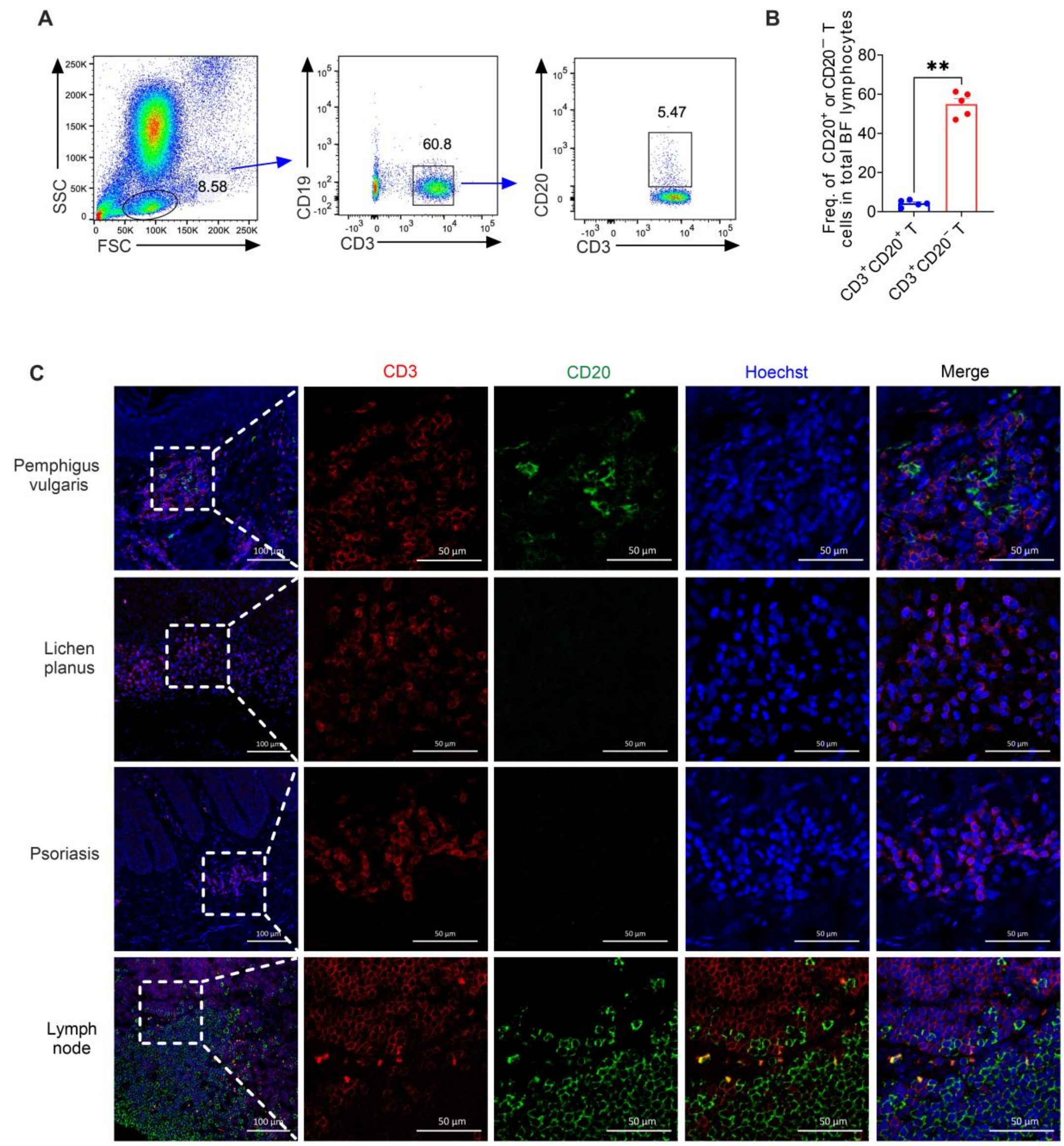

Supplemental Figure 3. CD20^{+} T cells in blister fluid from BP patients and other autoimmune and inflammatory skin diseases. **(A)** Representative flow cytometry plots and **(B)** frequencies of CD3^{+}CD20^{+} and CD3^{+}CD20^{-} T cells in blister fluid from BP patients (n = 5). **(C)** Representative immunofluorescence staining of CD3 (red), CD20 (green) and Hoechst (blue) in skin lesions from patients with pemphigus vulgaris, lichen planus, or psoriasis and in human lymph node. Scale bar =100 μm; Scale bar insert = 50 μm. All data are presented as the mean ± SD. An unpaired 2-tailed Student's *t* test was used for B. $^{**}P < 0.01$.

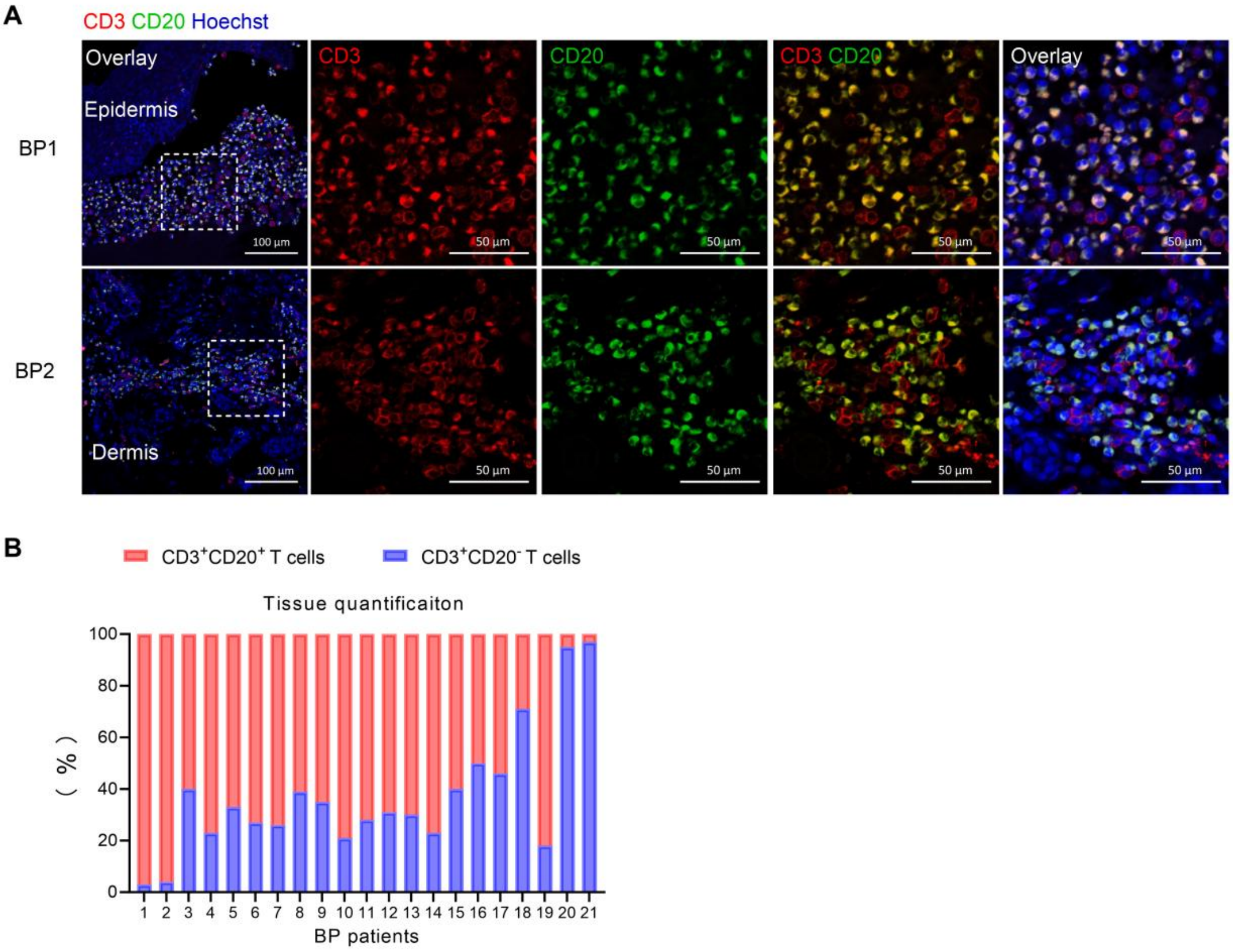

Supplemental Figure 4. Quantification of $CD3^+CD20^+$ and $CD3^+CD20^-$ T cells in BP lesions. (**A**) Representative immunofluorescence staining of CD3 (red), CD20 (green) and Hoechst (blue) in skin lesions from patients with BP. Scale bar =100 μm; Scale bar insert = 50 μm. (**B**) Percentages of $CD3^+CD20^+$ and $CD3^+CD20^-$ T cells among total $CD3^+$ T cells in skin lesions from 21 BP patients.

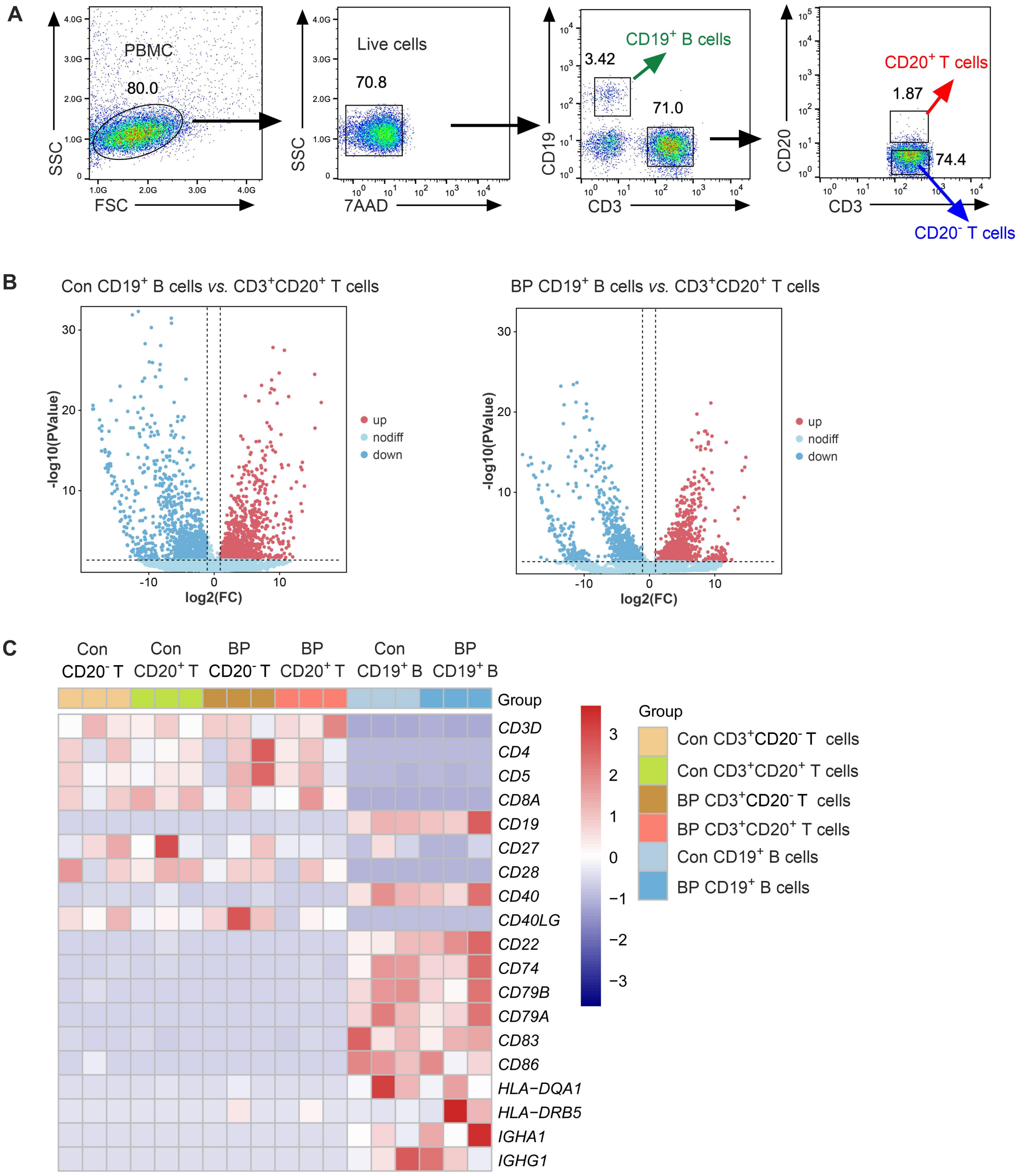

Supplemental Figure 5. Transcriptome characteristics of CD20$^+$ T cells. (A) Gating strategy for sorting CD3$^+$CD20$^+$ T cells, CD3$^+$CD20$^-$ T cells, and CD19$^+$ B cells from peripheral blood. **(B)** Volcano plot showing the number of differentially expressed genes (DEGs) between CD20$^+$ T cells and CD19$^+$ B cells from healthy controls (n = 3) and BP patients (n = 3). **(C)** Heatmap of DEGs among CD20$^+$ T cells, CD20$^-$ T cells and CD19$^+$ B cells from BP patients and healthy controls.

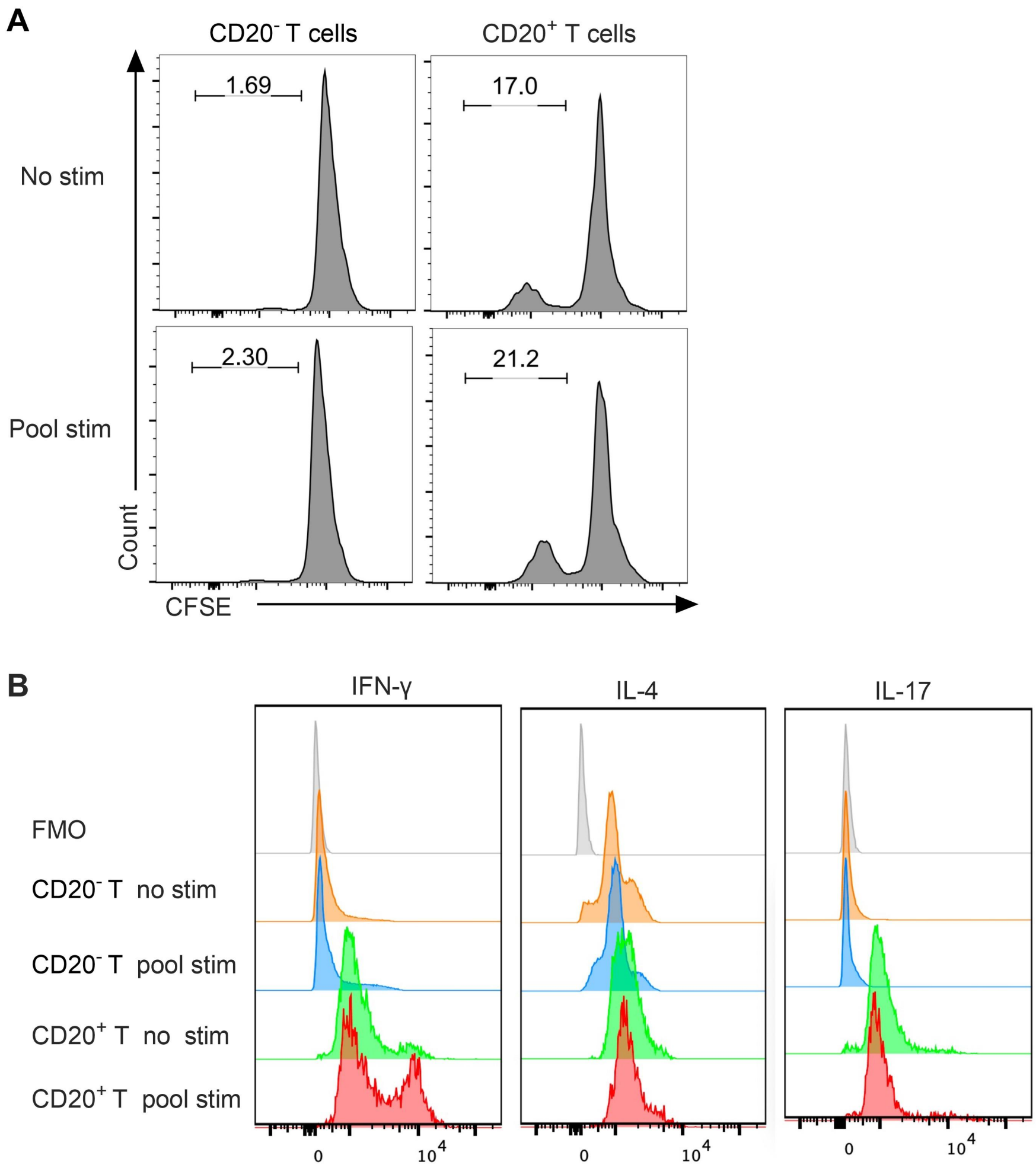

Supplemental Figure 6. Analysis of proliferation and intracellular cytokine expression in PBMCs with or without peptide stimulation. (**A**) Representative flow cytometry analysis of the percentages of proliferating $CD20^+$ and $CD20^-$ T cells in BP patients with or without peptide stimulation. (**B**) Representative flow cytometry analysis showing the expression of IFN-γ, IL-4, and IL-17 in $CD20^+$ and $CD20^-$ T cells from BP patients under the indicated conditions.

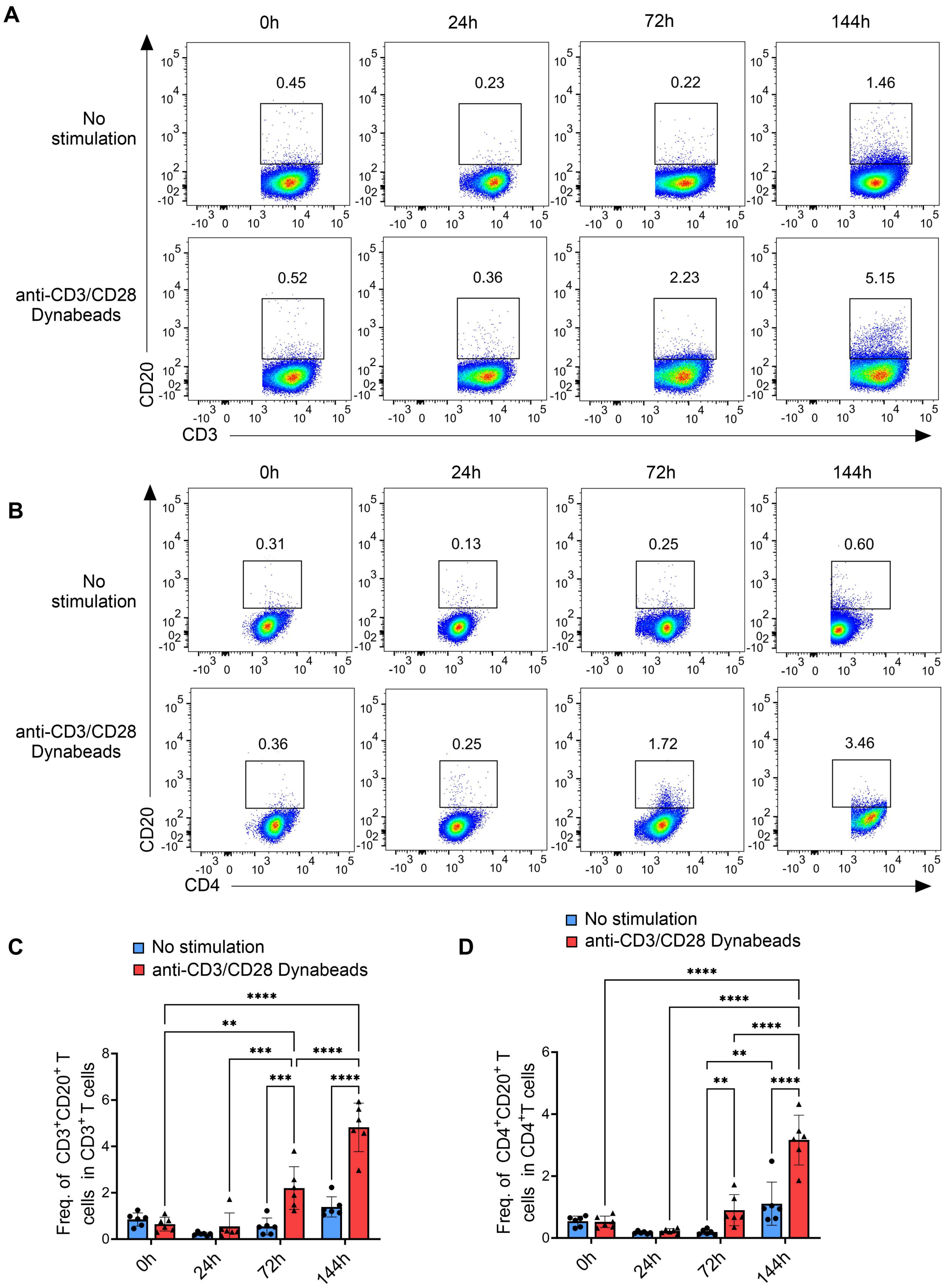

Supplemental Figure 7. TCR activation promotes CD20 expression on T cells. (**A**) Representative flow cytometry plots of $CD3^+CD20^+$ T cells and (**B**) $CD4^+CD20^+$ T cells among primary human T cells stimulated with anti-CD3/CD28 antibody- coated beads at the indicated time points. (**C**) Frequencies of $CD3^+CD20^+$ T cells and (**D**) $CD4^+CD20^+$ T cells under the indicated conditions (n = 6). All data are presented as the mean ± SD. Two-way ANOVA with Tukey's post hoc test was used for C and D. ***P* < 0.01, ****P* < 0.001, *****P* < 0.0001.

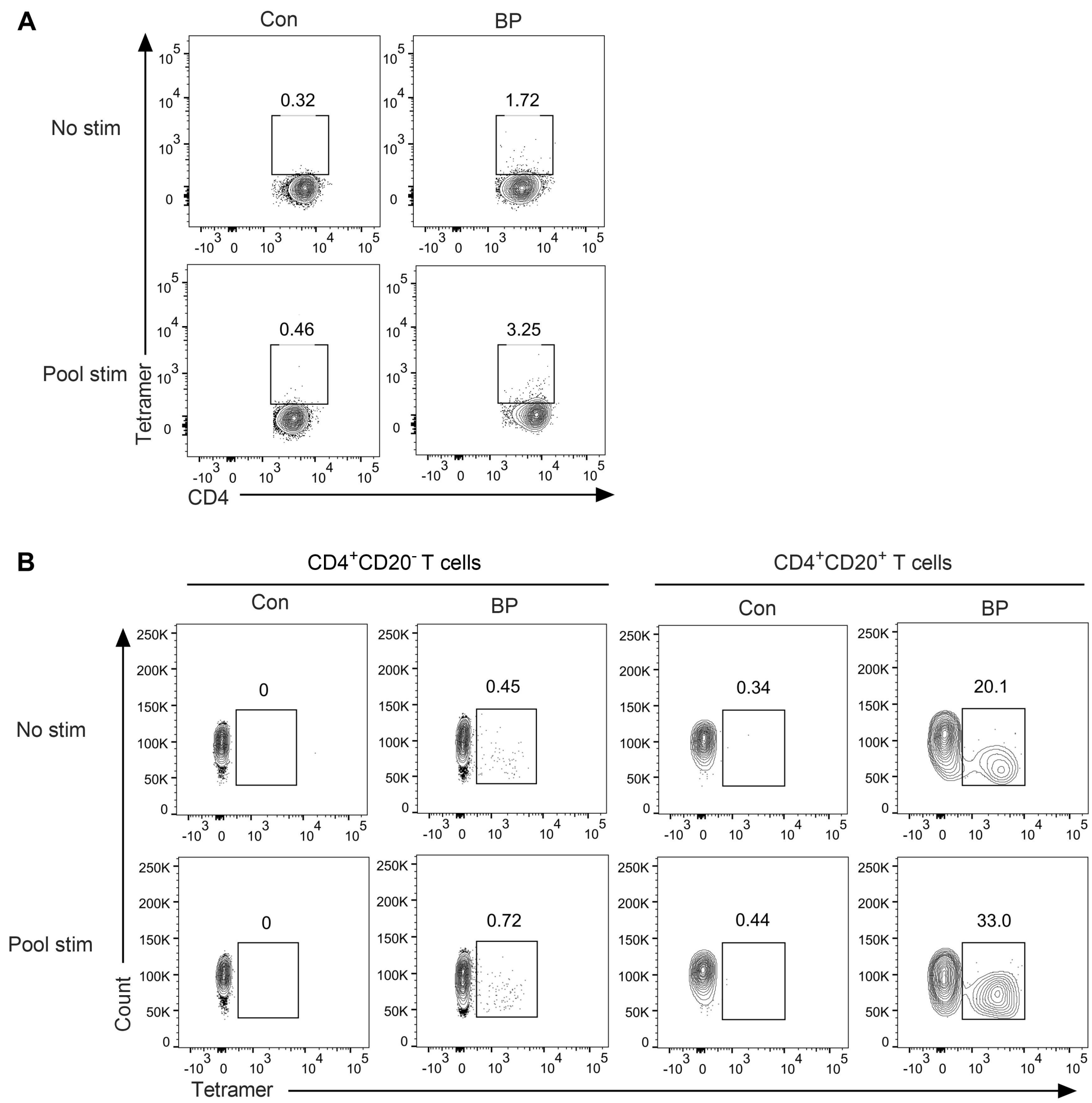

Supplemental Figure 8. Analysis of tetramer-positive CD4$^+$ T cells in PBMCs with or without peptide stimulation. (**A**) Representative flow cytometry plots showing tetramers and CD4 staining in PBMCs from untreated BP patients and HLA allele-matched healthy controls. (**B**) Representative flow cytometry plots showing tetramer-positive T cells within gated CD4$^+$CD20$^+$ and CD4$^+$CD20$^-$ T cell populations from untreated BP patients and HLA allele-matched healthy controls with or without peptide stimulation.

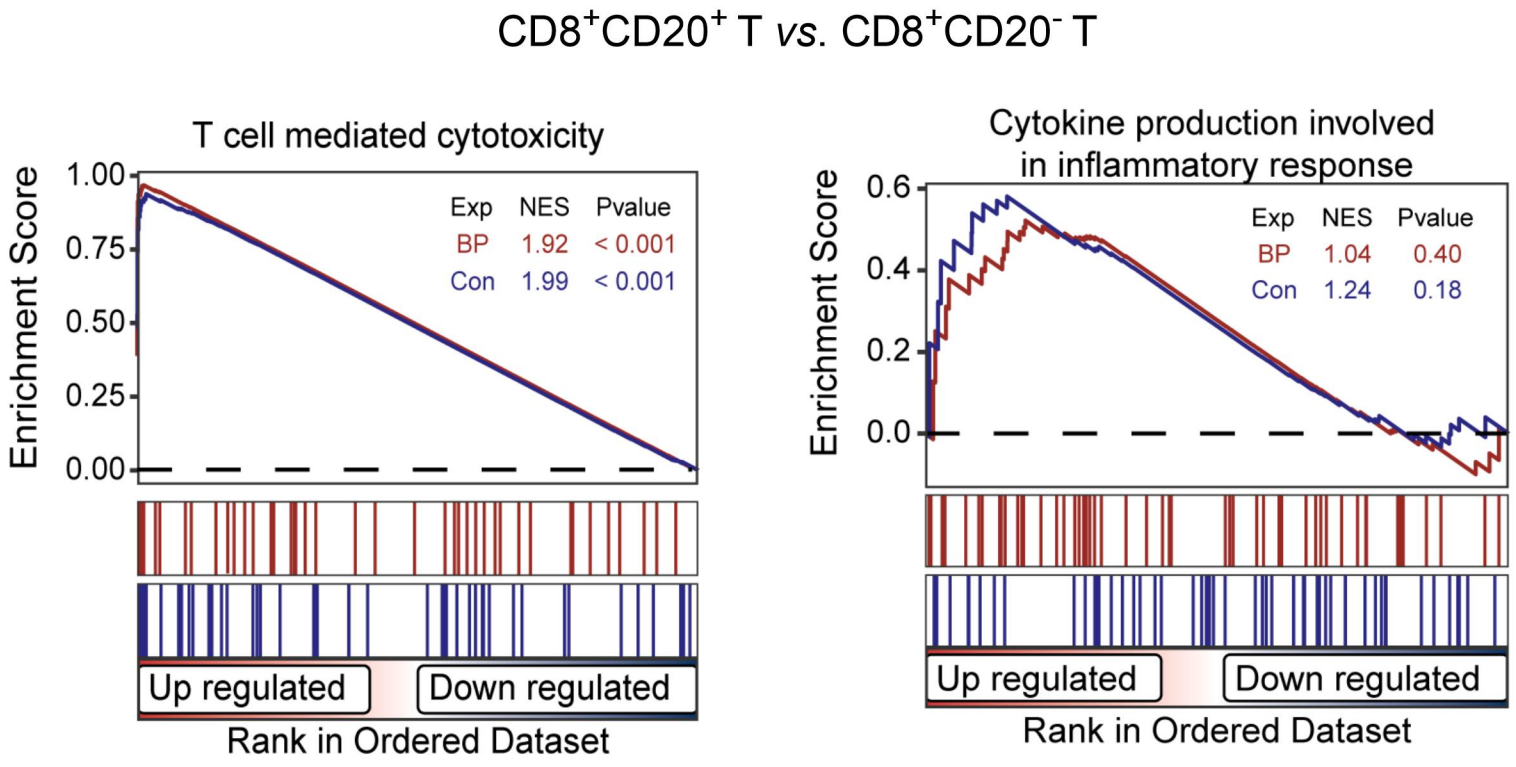

Supplemental Figure 9. GSEA enrichment plots for CD8$^+$CD20$^+$ T cells. GSEA showing enrichment of genes related to T cell mediated cytotoxicity and inflammatory response-related cytokine production in CD8$^+$CD20$^+$ T cells.

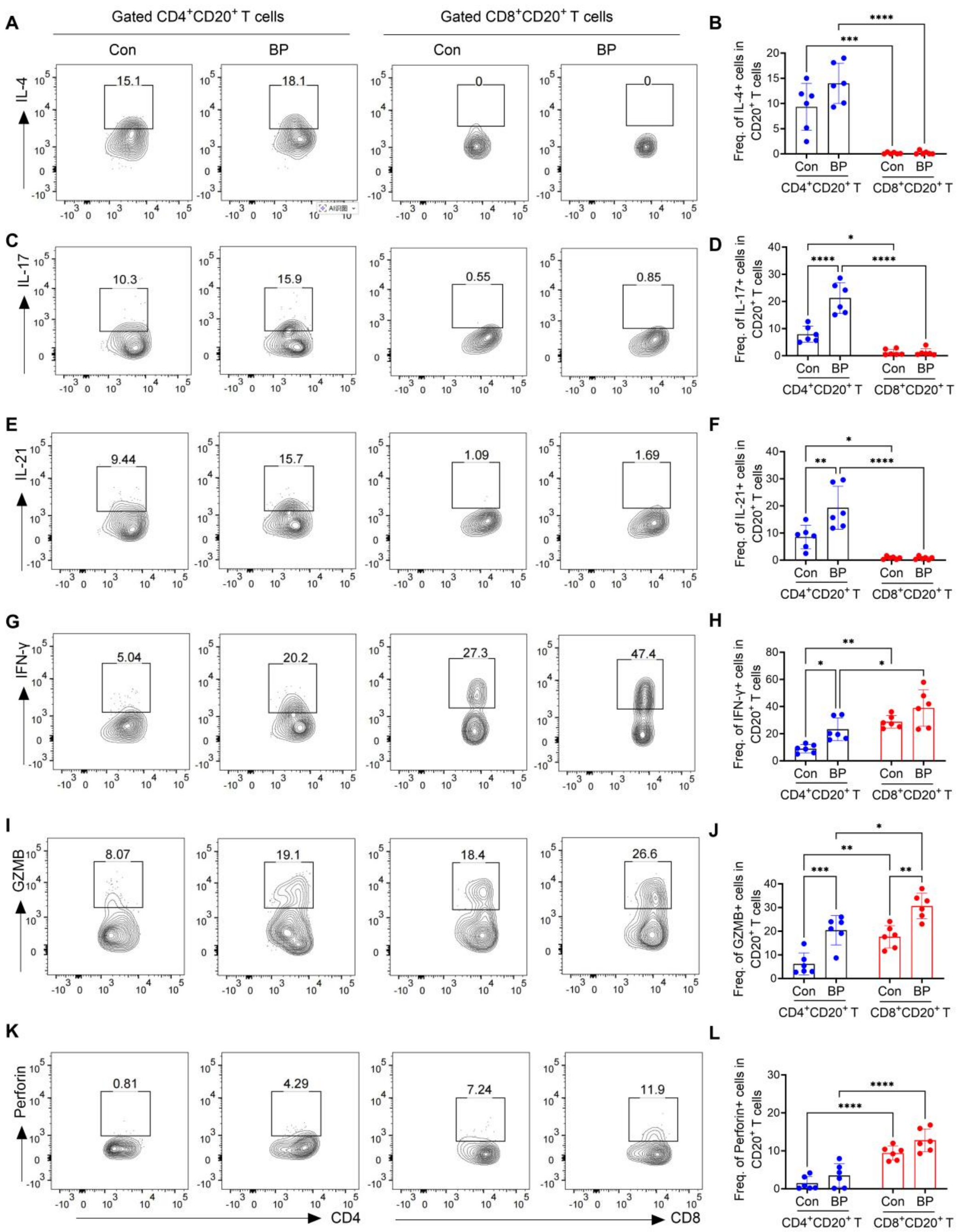

Supplemental Figure 10. Expression of intracellular cytokines in $CD4^+CD20^+$ and $CD8^+CD20^+$ T cells from healthy controls (Con) and BP patients. (**A**) Representative flow cytometry plots of IL-4^+ cells in $CD4^+CD20^+$ and $CD8^+CD20^+$ T cells from Con and BP patients. (**B**) Frequencies of IL-4^+ cells in $CD4^+CD20^+$ and $CD8^+CD20^+$ T cells from Con and BP patients (n = 6). (**C**) Representative flow cytometry plots of IL-17^+ cells in $CD4^+CD20^+$ and $CD8^+CD20^+$ T cells from Con and BP patients. (**D**) Frequencies of IL-17^+ cells in $CD4^+CD20^+$ and $CD8^+CD20^+$ T cells from Con and BP patients (n = 6). (**E**) Representative flow cytometry plots of IL-21^+ cells in $CD4^+CD20^+$ and $CD8^+CD20^+$ T cells from Con and BP patients. (**F**) Frequencies of IL-21^+ cells in $CD4^+CD20^+$ and $CD8^+CD20^+$ T cells from Con and BP patients (n = 6). (**G**)

Representative flow cytometry plots of IFN-γ^+ cells in $CD4^+CD20^+$ and $CD8^+CD20^+$ T cells from Con and BP patients. (**H**) Frequencies of IFN-γ^+ cells in $CD4^+CD20^+$ and $CD8^+CD20^+$ T cells from Con and BP patients (n = 6). (**I**) Representative flow cytometry plots of $GZMB^+$ cells in $CD4^+CD20^+$ and $CD8^+CD20^+$ T cells from Con and BP patients. (**J**) Frequencies of $GZMB^+$ cells in $CD4^+CD20^+$ and $CD8^+CD20^+$ T cells from Con and BP patients (n = 6). (**K**) Representative flow cytometry plots of $perforin^+$ cells in $CD4^+CD20^+$ and $CD8^+CD20^+$ T cells from Con and BP patients. (**L**) Frequencies of $perforin^+$ cells in $CD4^+CD20^+$ and $CD8^+CD20^+$ T cells from Con and BP patients (n = 6). All data are presented as the mean ± SD. Two-way ANOVA with Tukey's post hoc test was used for B, D, F, H, J and L. $*P < 0.05$, $**P < 0.01$, $***P < 0.001$, $****P < 0.0001$.

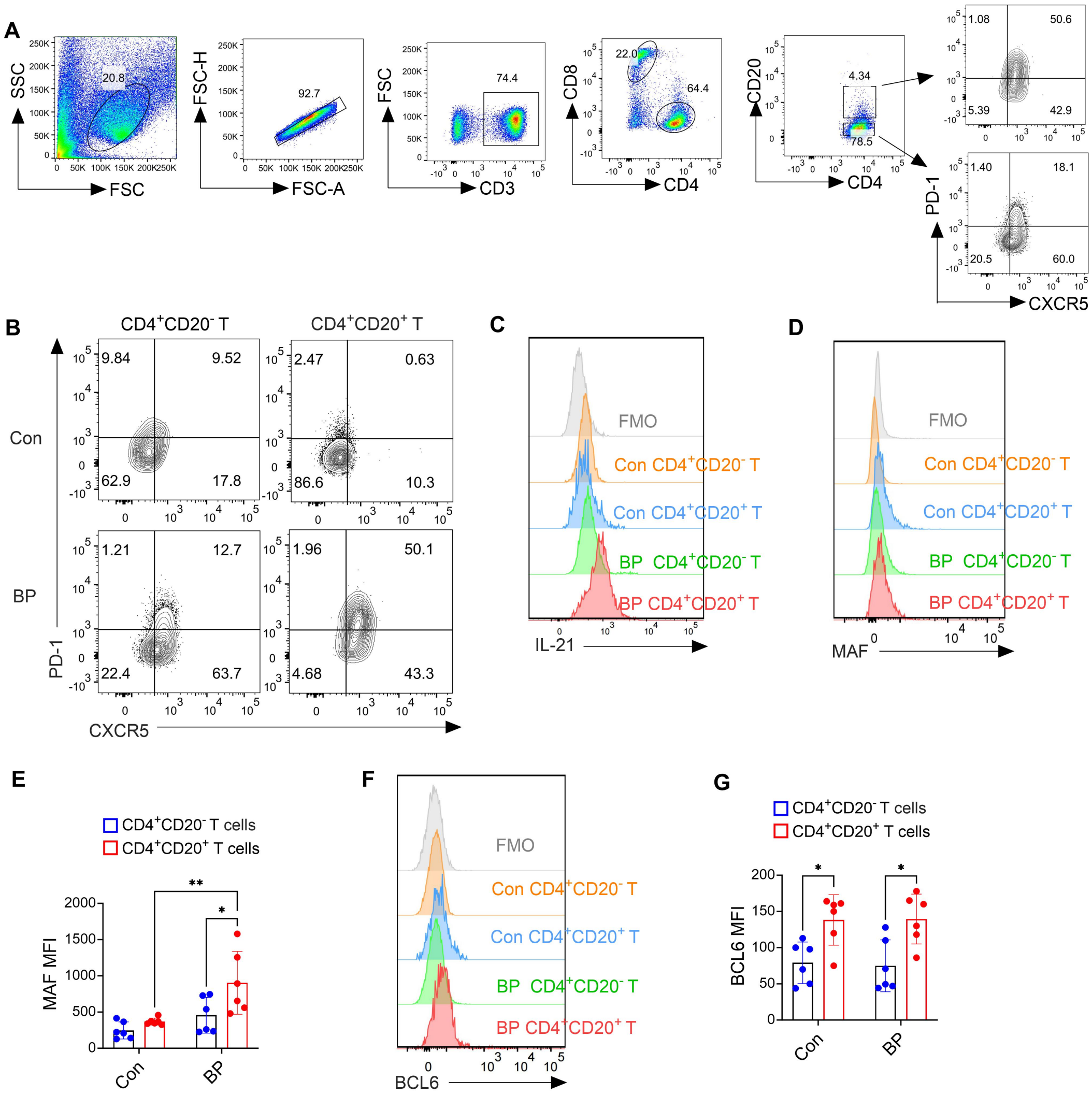

Supplemental Figure 11. Flow cytometry analysis of CXCR5⁺PD-1⁺ T cells, and IL-21, MAF, and BCL6 expression in peripheral blood CD4⁺CD20⁺ versus CD4⁺CD20⁻ T cell subsets from control and BP patients. (**A**) Gating strategy for the analysis of CXCR5⁺PD-1⁺ T cells within CD4⁺CD20⁺ and CD4⁺CD20⁻ T cell subsets. (**B**) Representative flow cytometry dot plots showing CXCR5 and PD-1 expression on gated CD4⁺CD20⁺ and CD4⁺CD20⁻ T cells from Con and BP patients. (**C**) Representative flow cytometry histograms of IL-21 expression in CD4⁺CD20⁺ and CD4⁺CD20⁻ T cells. (**D**) Representative flow cytometry histograms of MAF expression in CD4⁺CD20⁺ and CD4⁺CD20⁻ T cells. (**E**) Quantification of MAF mean fluorescence intensity (MFI) of MAF in CD4⁺CD20⁺ versus CD4⁺CD20⁻ T cells from the Con and BP groups (n = 6). (**F**) Representative flow cytometry histograms of BCL6 expression in CD4⁺CD20⁺ and CD4⁺CD20⁻ T cells from Con and BP patients. (**G**) Quantification of BCL6 MFI in CD4⁺CD20⁺ versus CD4⁺CD20⁻ T cells from the Con and BP groups (n = 6). All data are presented as the mean ± SD. Two-way ANOVA with Tukey's post hoc test was used for E and G. **P < 0.05, **P < 0.01.*

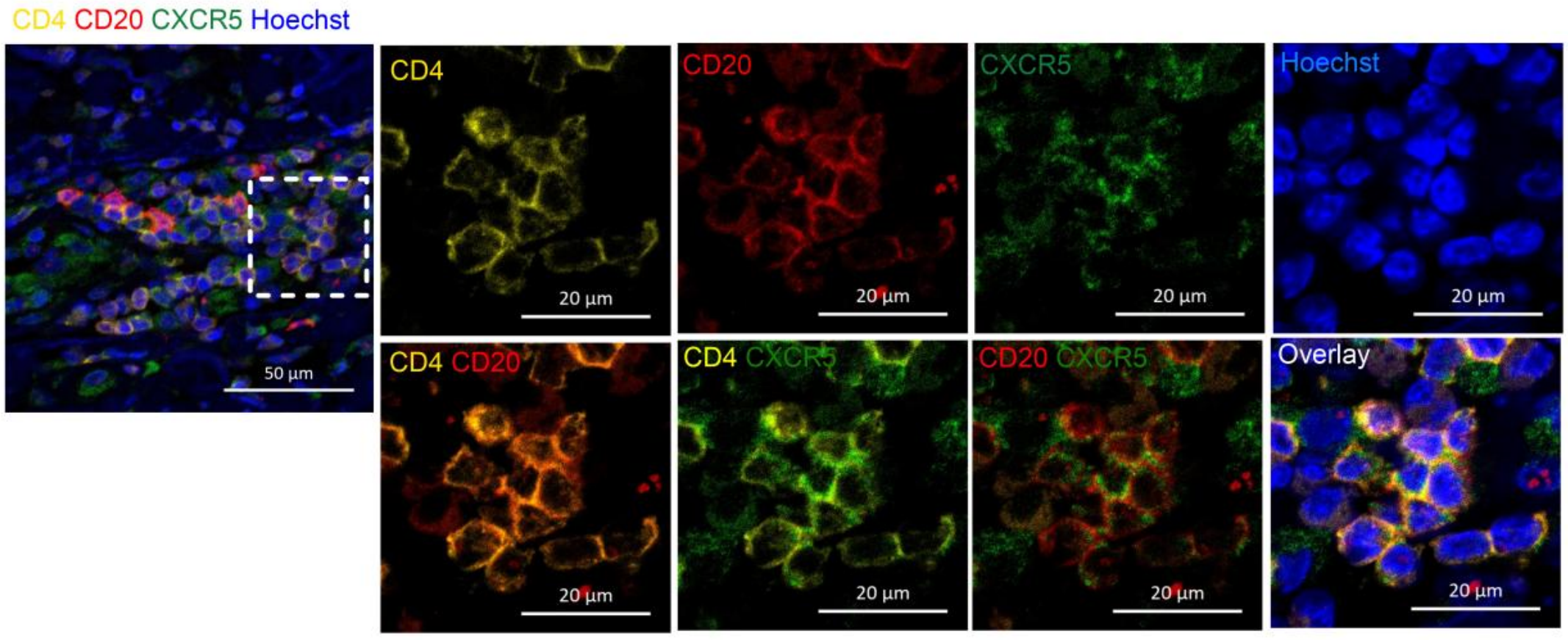

Supplemental Figure 12. Detection of CD4$^+$CXCR5$^+$CD20$^+$ T cells in BP patient skin lesions by immunofluorescence. Representative immunofluorescence staining of CD4 (yellow), CXCR5 (green), CD20 (red) and DNA (Hoechst, blue) in BP skin lesions. Scale bar = 50 µm; Scale bar insert = 20 µm.

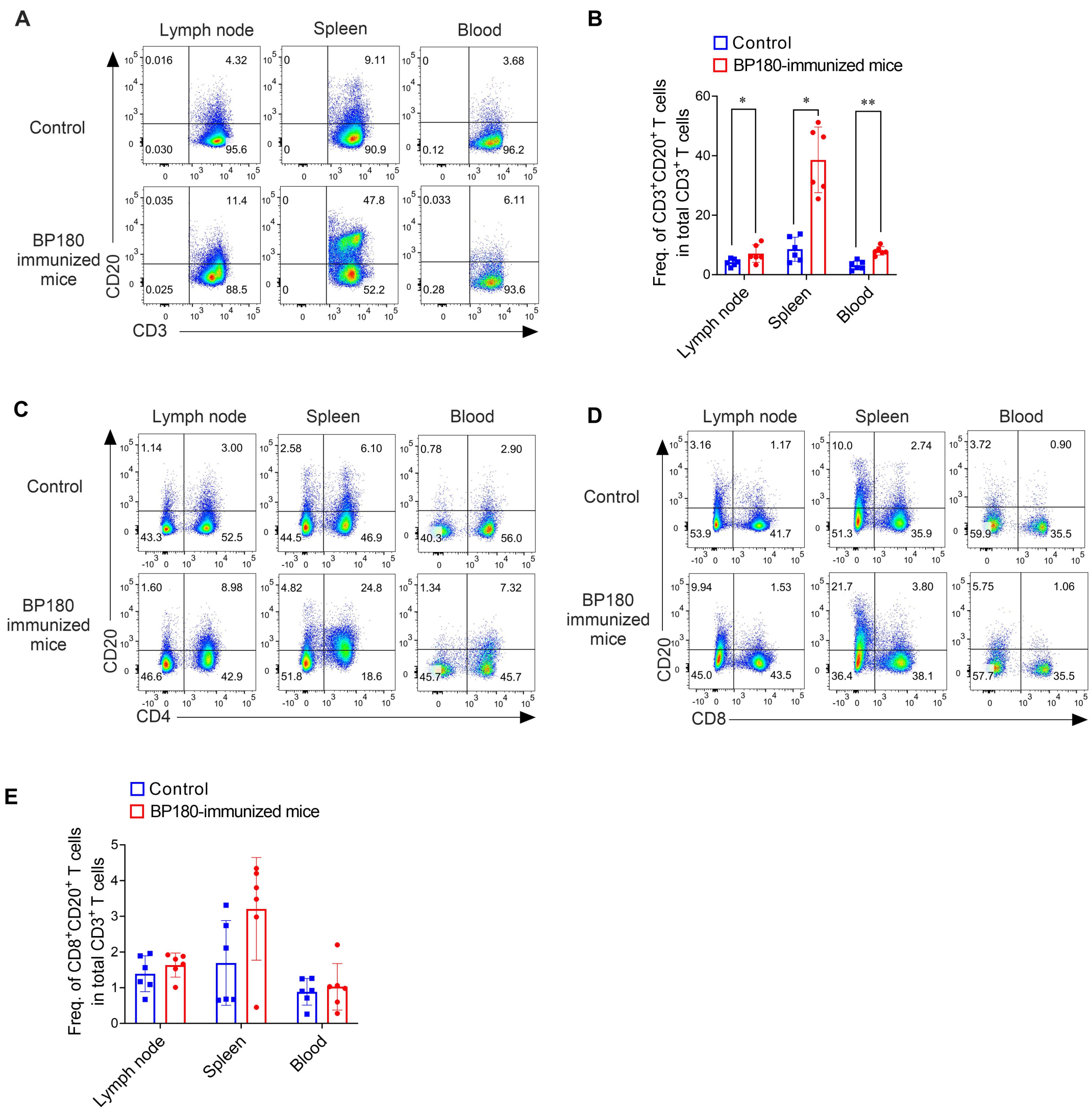

Supplemental Figure 13. Frequencies of $CD3^+CD20^+$ T cells and their subsets in control and BP180-immunized mice. (**A**) Representative flow cytometry plots of $CD3^+CD20^+$ T cells among total T cells in the spleen, lymph nodes, and peripheral blood of control and BP180-immunized mice. (**B**) Frequencies of $CD3^+CD20^+$ T cells among total T cells in the spleen, lymph nodes, and peripheral blood of control and BP180-immunized mice (n = 6). (**C**) Representative flow cytometry plots of $CD4^+CD20^+$ T cells among total T cells in the spleen, lymph nodes, and peripheral blood of control and BP180-immunized mice. (**D**) Representative flow cytometry plots of $CD8^+CD20^+$ T cells among total T cells in the spleen, lymph nodes, and peripheral blood of control and BP180-immunized mice. (**E**) Frequencies of $CD8^+CD20^+$ T cells among total T cells in the spleen, lymph nodes, and peripheral blood of control and BP180-immunized mice (n = 6). All data are presented as the mean ± SD. An unpaired 2-tailed Student's *t* test was used for B and E. *$^*P < 0.05$, $^{**}P < 0.01$.*

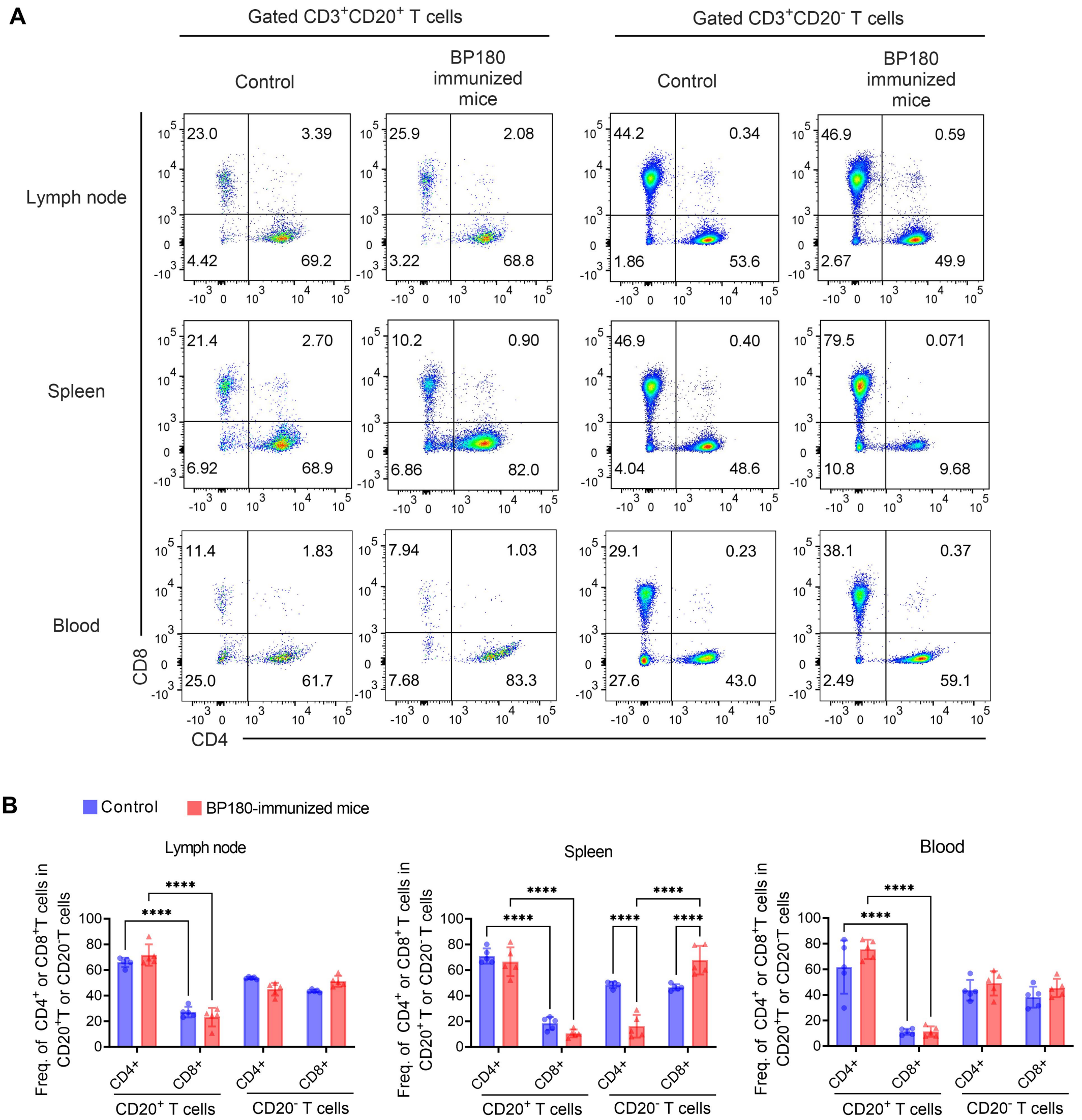

Supplemental Figure 14. Frequencies of CD4⁺ or CD8⁺ subsets within CD3⁺CD20⁺ T cells in control and BP180-immunized mice. (**A**) Representative flow cytometry plots showing $CD4^+$ and $CD8^+$ T cells within gated $CD3^+CD20^+$ T cells in the spleen, lymph nodes, and peripheral blood of control and BP180-immunized mice. (**B**) Frequencies of $CD4^+$ or $CD8^+$ T cells within $CD3^+CD20^+$ T cells in the spleen, lymph nodes, and peripheral blood of control and BP180-immunized mice (n = 5). All data are presented as the mean ± SD. Two-way ANOVA with Tukey's post hoc test was used for B. *****P < 0.0001*.

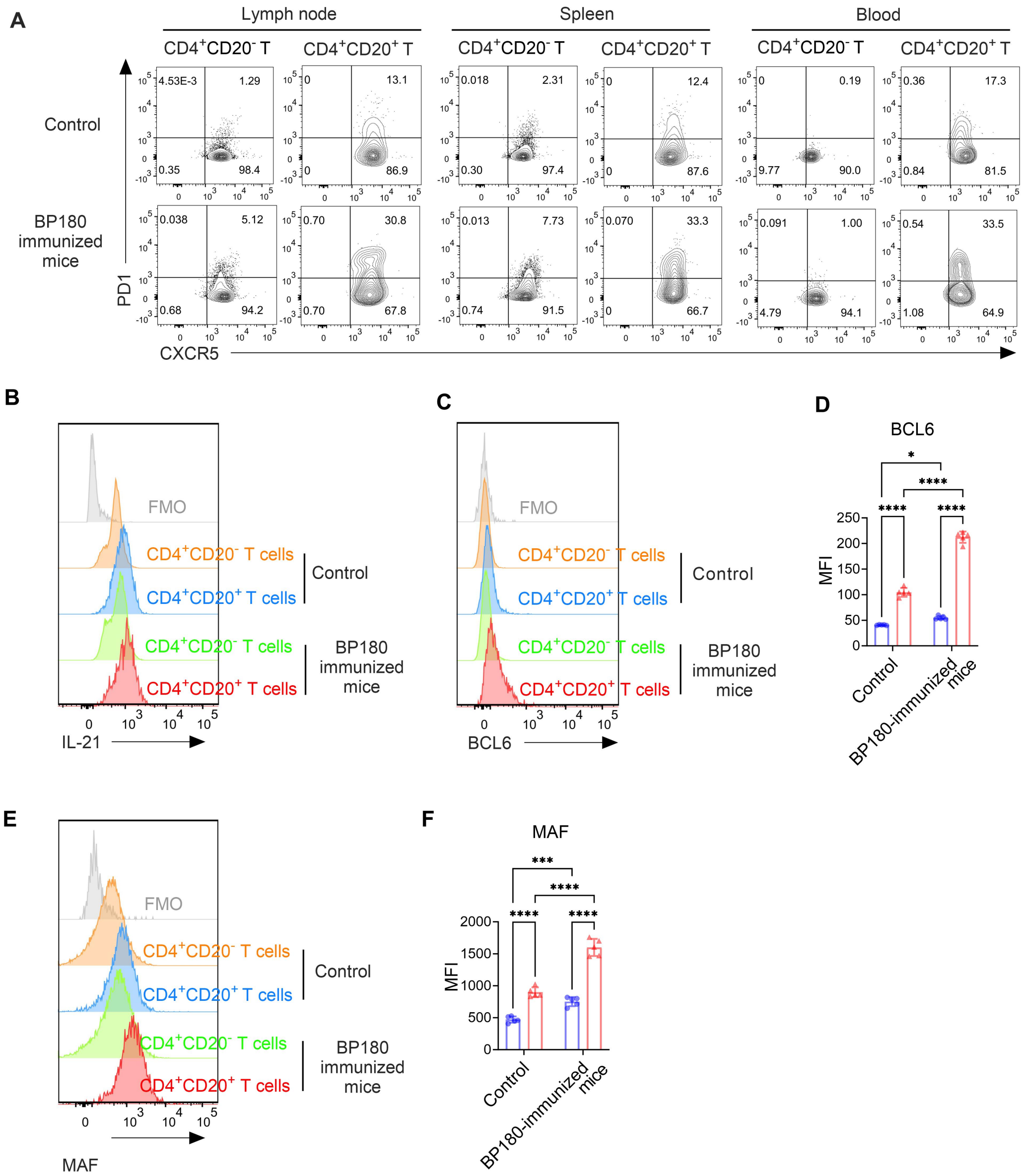

Supplemental Figure 15. Frequencies of CXCR5$^+$PD-1$^+$ T cells and expression of IL-21, BCL6 and MAF in CD4$^+$CD20$^+$ versus CD4$^+$CD20$^-$ T cells from control and BP180-immunized mice. (**A**) Representative flow cytometry plots of CXCR5$^+$PD-1$^+$ T cells gated on CD4$^+$CD20$^+$ or CD4$^+$CD20$^-$ T cells in the spleen, lymph nodes, and peripheral blood of control and BP180-immunized mice. (**B**) Representative flow cytometry histograms of IL-21 expression in the indicated cell types. (**C**) Representative flow cytometry histograms of BCL6 expression in the indicated cell types. (**D**) Quantification of BCL6 MFI in CD4$^+$CD20$^+$ versus CD4$^+$CD20$^-$ T cells in the spleen of control and BP180-immunized mice (n = 5). (**E**) Representative flow cytometry histograms of MAF expression in the indicated cell types. (**F**) Quantification of MAF MFI in CD4$^+$CD20$^+$ versus CD4$^+$CD20$^-$ T cells in the spleen of control and BP180-immunized mice (n = 5). All data are presented as the mean ± SD. Two-way ANOVA with Tukey's post hoc test was used for D and F. *$P < 0.05$, ***$P < 0.001$, ****$P < 0.0001$.

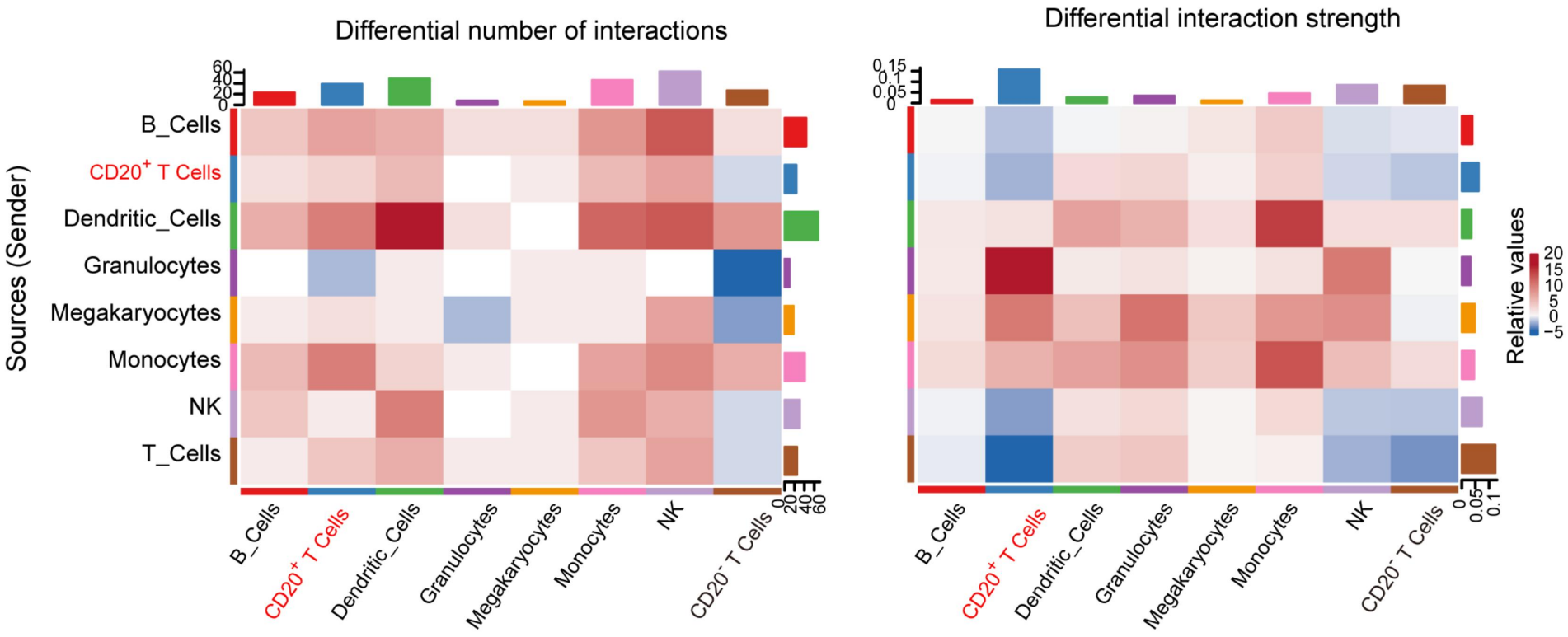

Supplemental Figure 16. Single-cell sequencing analysis of the interaction between CD20⁺ T cells and other immune cells in human peripheral blood. Heatmaps of differential cell–cell communication patterns in BP patients versus healthy controls. Left: Differential number of interactions; Right: Differential interaction strength.

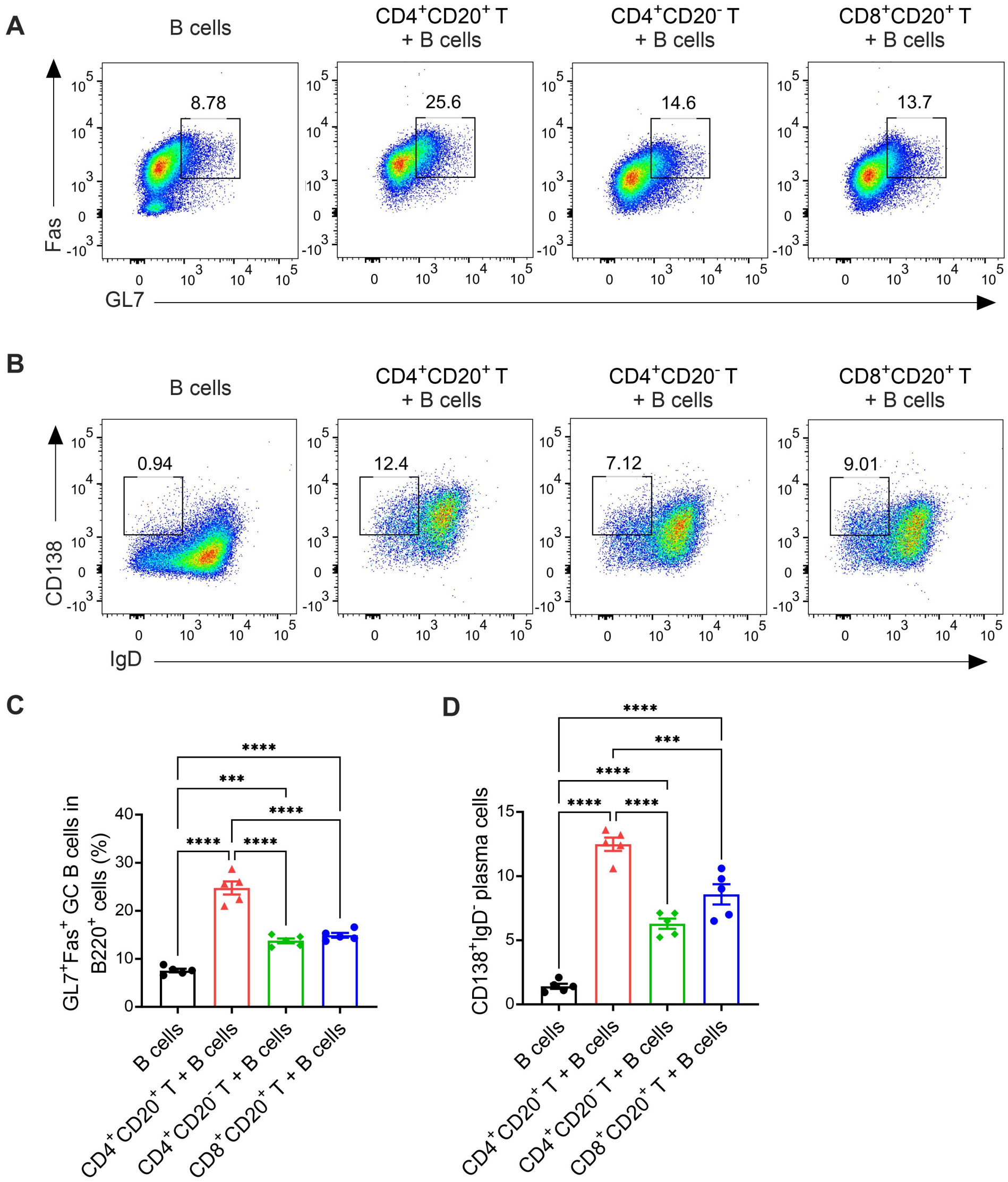

Supplemental Figure 17. CD4$^+$CD20$^+$ T cells promote B cell differentiation and antibody production ex vivo. CD4$^+$CD20$^+$ T cells, CD4$^+$CD20$^-$ T cells, CD8$^+$CD20$^+$ T cellswere isolated from the spleen and lymph nodes of BP180- immunized mice and then cocultured with B cells at a 1:1 ratio. (**A**) Representative flow cytometry plots showing GL7$^+$Fas$^+$ germinal center (GC) B cells gated on B220$^+$ B cells in the indicated groups. (**B**) Representative flow cytometry plots of IgD$^-$CD138$^+$ plasma cells in the indicated groups. (**C**) Frequencies of GL7$^+$Fas$^+$ GC B cells and (**D**) IgD$^-$CD138$^+$ plasma cells in the co-culture system (n = 5). All data are presented as the mean ± SD. One-way ANOVA with Tukey's post hoc test was used for C and D. ***P < 0.001, ****P < 0.0001.

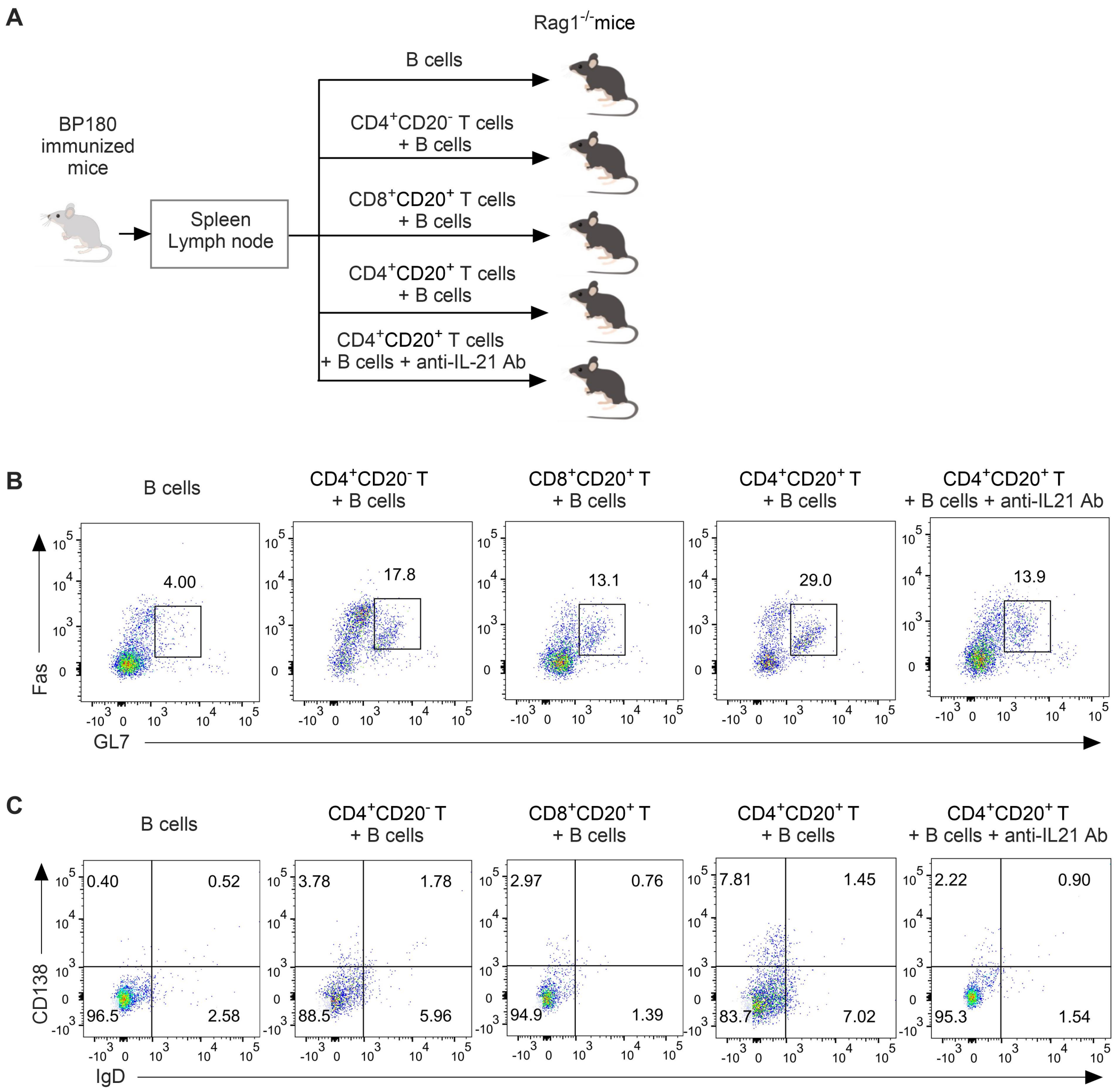

Supplemental Figure 18. Flow cytometry analysis of GL7$^+$Fas$^+$ GC B cells and IgD$^-$CD138$^+$ plasma cells in the spleens of recipient Rag1$^{-/-}$ mice after adoptive transfer. (**A**) Schematic diagram of the adoptive transfer experiment. CD4$^+$CD20$^+$ T cells, CD4$^+$CD20$^-$ T cells, CD8$^+$CD20$^+$ T cells and B220$^+$ B cells were sorted from BP180-immunized mice and transferred into Rag1$^{-/-}$ mice. Splenocytes were harvested for flow cytometry analysis 14 days post-transfer. (**B**) Representative flow cytometry plots showing GL7$^+$Fas$^+$ GC B cells gated on B220$^+$ B cells in the spleens of recipient mice. (**C**) Representative flow cytometry plots of IgD$^-$CD138$^+$ plasma cells in the spleens of recipient mice.

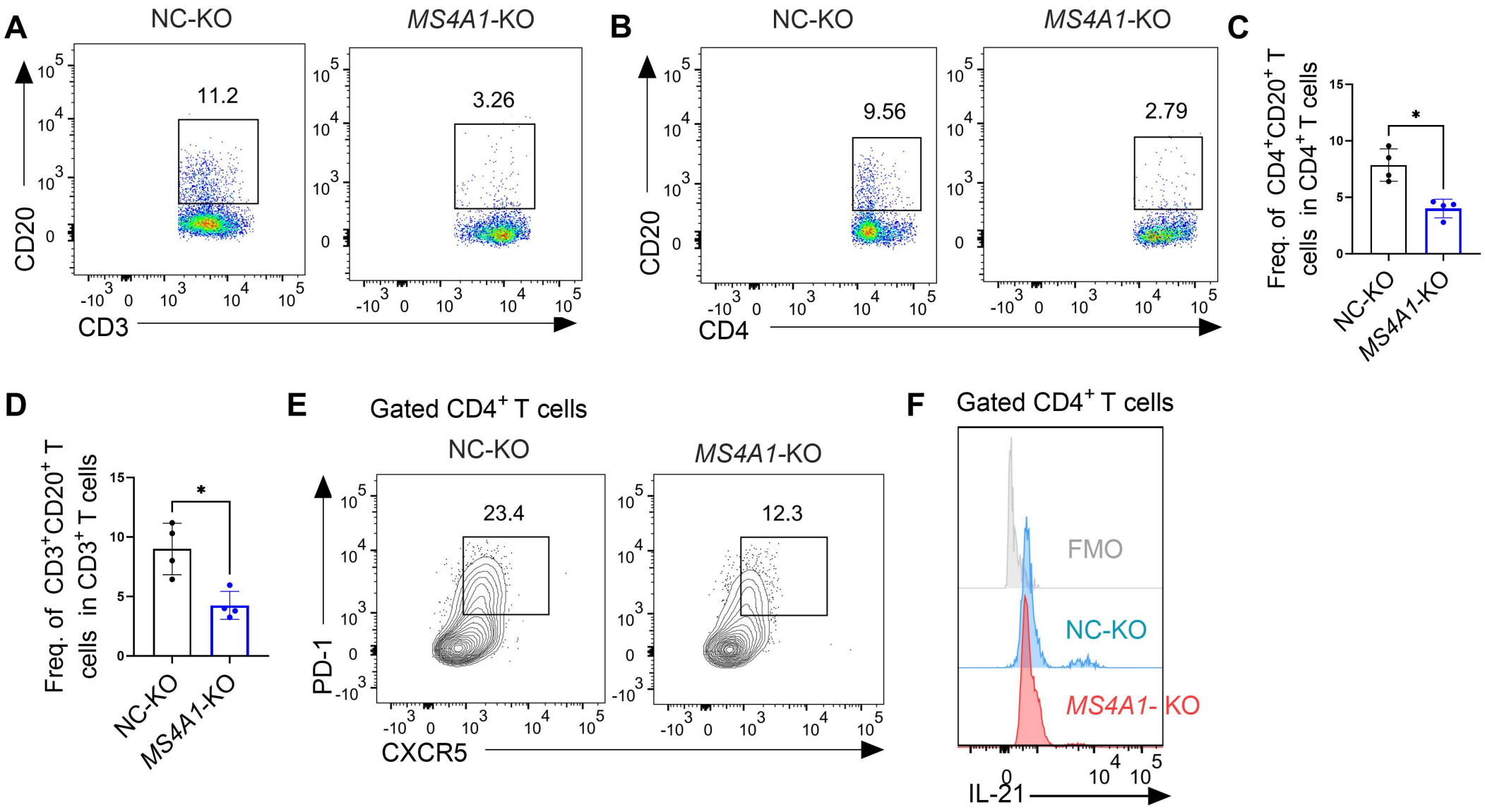

Supplemental Figure 19. The effect of *MS4A1* knockout on T cell subsets and function. (**A**) Representative flow cytometry plots of CD3$^+$CD20$^+$ T cells gated on total T cells from control and *MS4A1*-knockout T cells. (**B**) Representative flow cytometry plots of CD4$^+$CD20$^+$ T cells from control and *MS4A1*-knockout T cells. (**C**) Frequencies of CD4$^+$CD20$^+$ T cells in control and *MS4A1*-knockout T cells (n = 4). (**D**) Frequencies of CD3$^+$CD20$^+$ T cells in control and *MS4A1*-knockout T cells (n = 4). (**E**) Representative flow cytometry dot plots showing CXCR5$^+$PD-1$^+$ T cells gated on CD4$^+$ T cells form control and *MS4A1*-knockout T cells. (**F**) Representative flow cytometry histograms of intracellular IL-21 expression in CD4$^+$ T cells from control and *MS4A1*-knockout T cells. All data are presented as the mean ± SD. An unpaired 2-tailed Student's *t* test was used for C and D. $^*P < 0.05$.

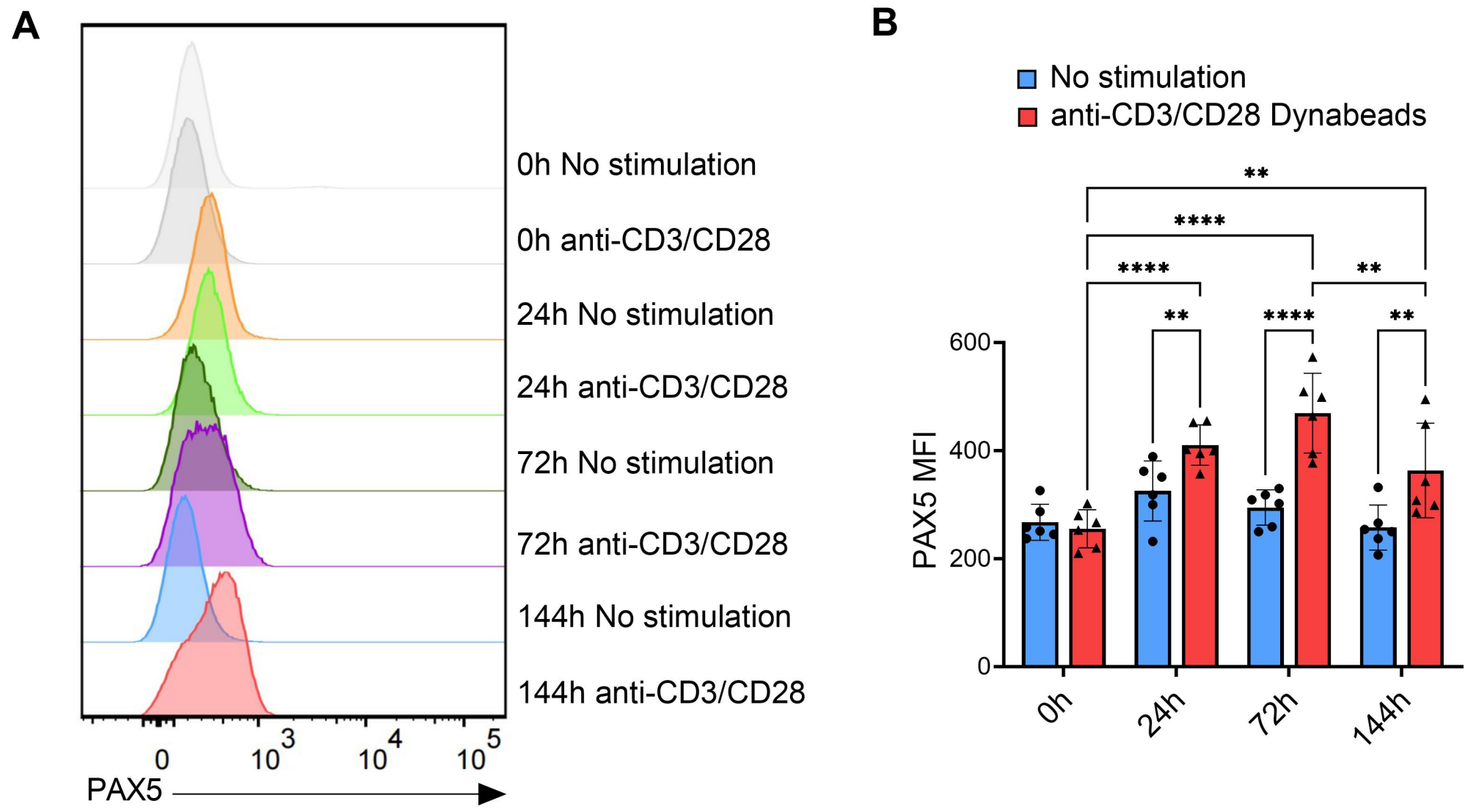

Supplemental Figure 20. TCR activation upregulates PAX5 expression in $CD3^+$ T cells. $CD3^+$ T cells were stimulated with or without anti-CD3/CD28 Dynabeads, and PAX5 expression was examined at the indicated time points.(**A**) Representative flow cytometry histograms of PAX5 expression in stimulated versus unstimulated $CD3^+$ T cells at the indicated time points (n = 6). (**B**) Quantification of PAX5 MFI in $CD3^+$ T cells under the indicated conditions (n = 6). All data are presented as the mean ± SD. Two-way ANOVA with Tukey's post hoc test was used for B. $^{**}P < 0.01$, $^{****}P < 0.0001$.

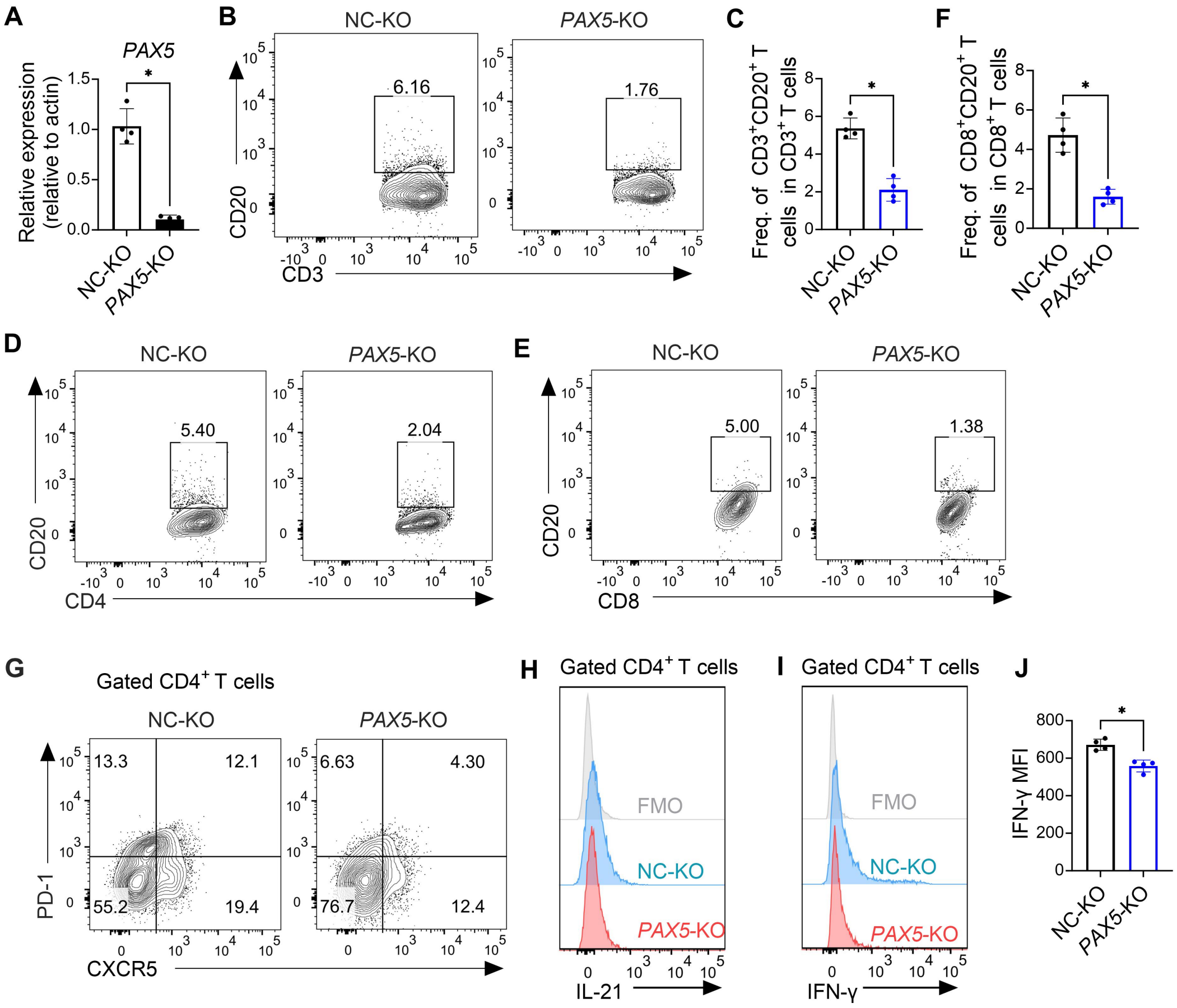

Supplemental Figure 21. *PAX5* knockout in T cells reduces the proportion of CD20$^+$ T cells and suppresses Tfh-related functions. (**A**) *PAX5* mRNA expression in control and *PAX5*-knockout T cells activated with anti-CD3/CD28 Dynabeads (n = 4). (**B**) Representative flow cytometry plots and (**C**) frequencies of CD3$^+$CD20$^+$ T cells in control and *PAX5*-knockout T cells (n = 4). (**D**) Representative flow cytometry plots of CD4$^+$CD20$^+$ T cells and (**E**) CD8$^+$CD20$^+$ T cells in control and *PAX5*-knockout T cells. (**F**) Frequencies of CD8$^+$CD20$^+$ T cells in control and *PAX5*-knockout T cells (n = 4). (**G**) Representative flow cytometry dot plots showing CXCR5$^+$PD-1$^+$ T cells gated on CD4$^+$ T cells in control and *PAX5*-knockout T cells. (**H**) Representative flow cytometry histograms of intracellular IL-21 production in CD4$^+$ T cells. (**I**) Representative flow cytometry histograms of IFN-γ in CD4$^+$ T cells. (**J**) Quantification of IFN-γ MFI of in CD4$^+$ T cells (n = 4). All data are presented as the mean ± SD. An unpaired 2-tailed Student's *t* test was used for A, C, F and J. *$P < 0.05$.

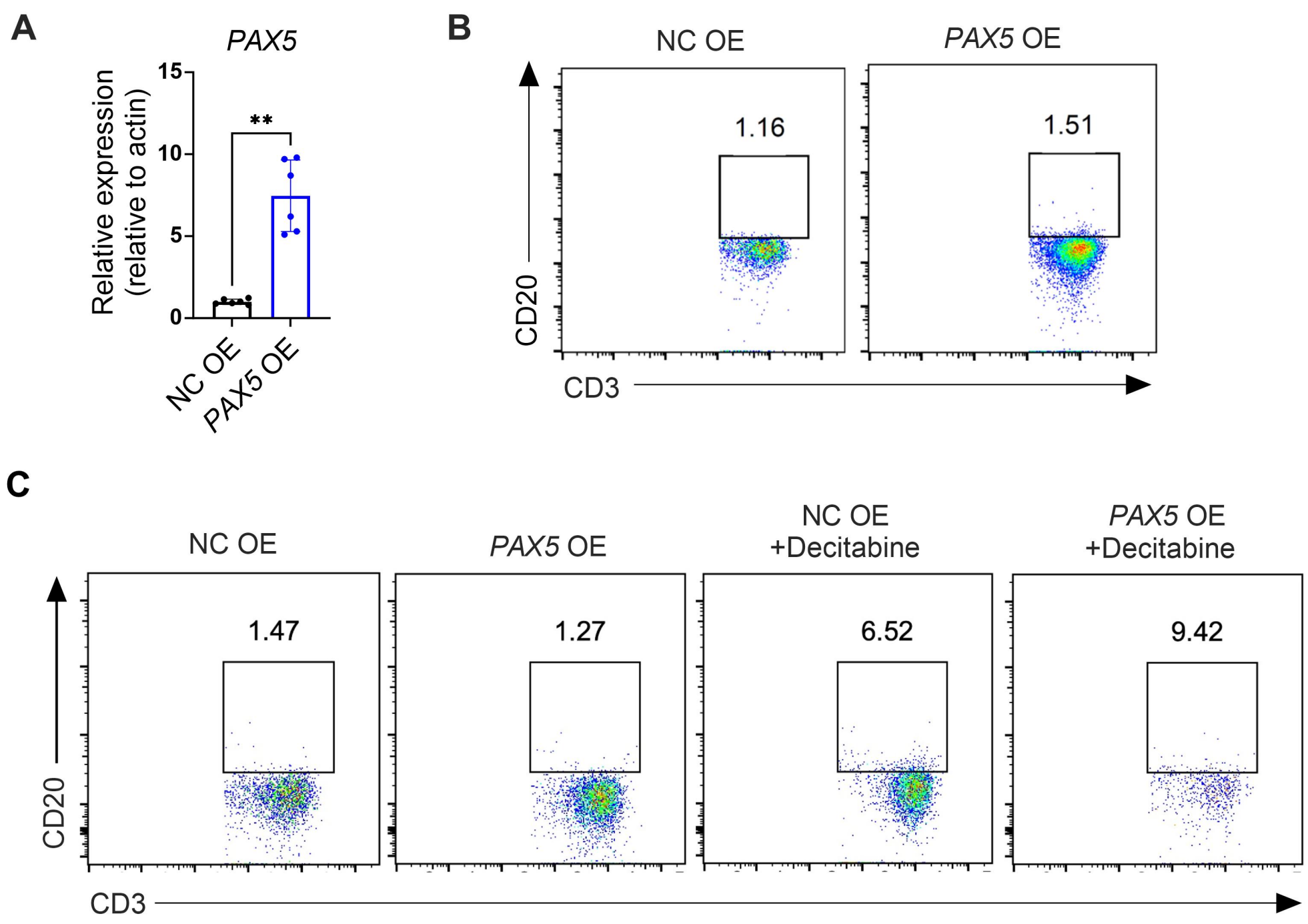

Supplemental Figure 22. DNA methylation and PAX5 govern CD20^{+} T cell differentiation. (A) *PAX5* mRNA expression in control and *PAX5*-overexpressing T cells activated with anti-CD3/CD28 Dynabeads (n = 6). (B) Representative flow cytometry plots of CD20^{+} T cells in control and *PAX5*-overexpressing T cells. (C) Representative flow cytometry plots of CD20^{+} T cells in control and *PAX5*-overexpressing T cells treated with or without decitabine. All data are presented as the mean ± SD. An unpaired 2-tailed Student's *t* test was used for A. $^{**}P < 0.01$.

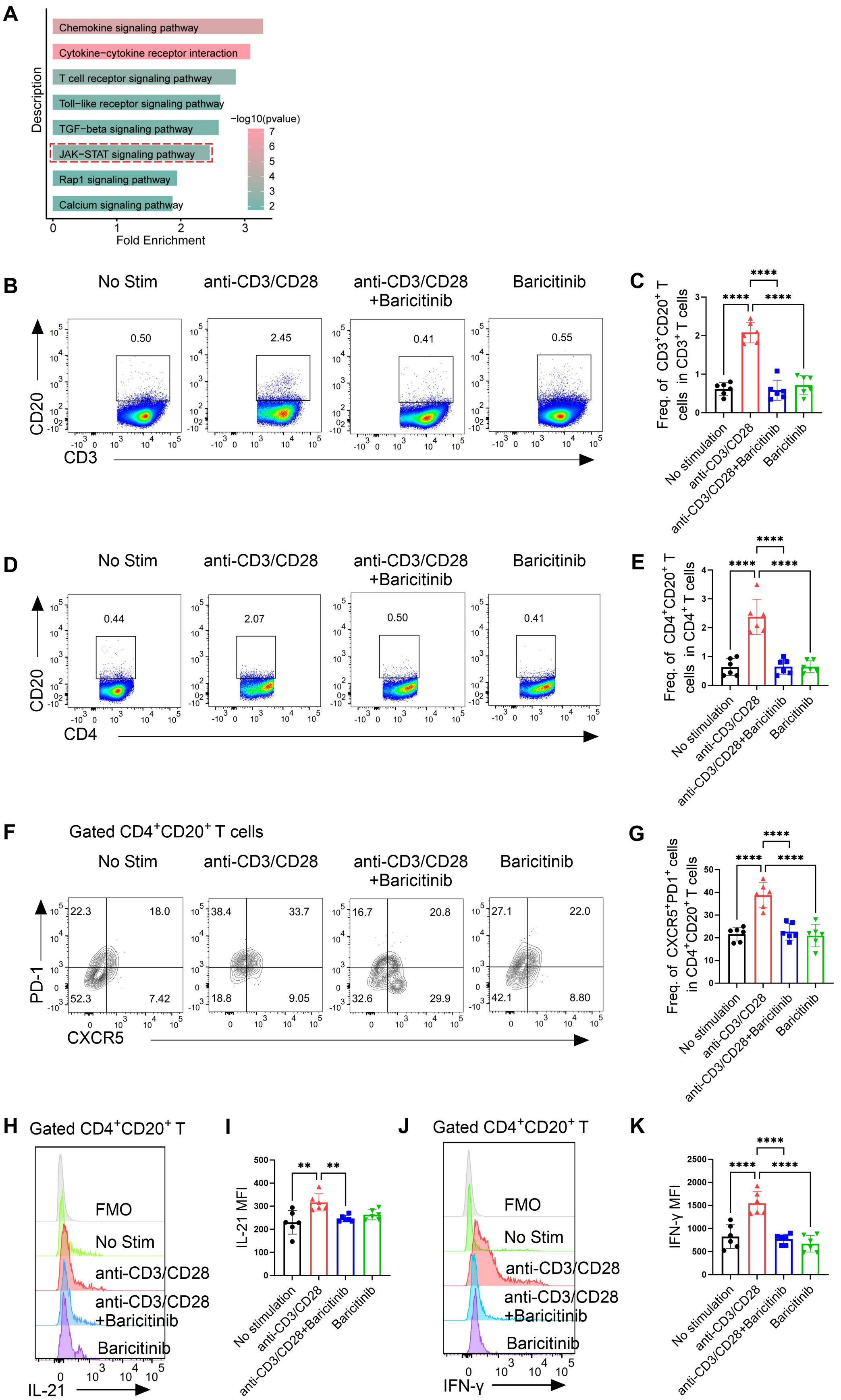
A
Chemokine signaling pathway
Cytokine−cytokine receptor interaction
T cell receptor signaling pathway
Toll−like receptor signaling pathway
TGF−beta signaling pathway
JAK−STAT signaling pathway
Rap1 signaling pathway
Calcium signaling pathway
Description
Fold Enrichment
−log10(pvalue)
B
No Stim
anti-CD3/CD28
anti-CD3/CD28 +Baricitinib
Baricitinib
0.50
2.45
0.41
0.55
CD20
CD3
C
Freq. of CD3+CD20+ T cells in CD3+ T cells
No stimulation
anti-CD3/CD28
anti-CD3/CD28+Baricitinib
Baricitinib
D
0.44
2.07
0.50
0.41
CD4
E
Freq. of CD4+CD20+ T cells in CD4+ T cells
F
Gated CD4+CD20+ T cells
22.3
18.0
52.3
7.42
38.4
33.7
18.8
9.05
16.7
20.8
32.6
29.9
27.1
22.0
42.1
8.80
PD-1
CXCR5
G
Freq. of CXCR5+PD1+ cells in CD4+CD20+ T cells
H
Gated CD4+CD20+ T
FMO
IL-21
I
IL-21 MFI
J
IFN-γ
K
IFN-γ MFI

Supplemental Figure 23. Inhibition of the JAK-STAT signaling pathway attenuates the frequencies of $CD20^+$ T cells. (**A**) KEGG pathway enrichment analysis of differentially expressed genes in $CD4^+CD20^+$ T cells compared with $CD4^+CD20^-$ T cells. (**B**) Representative flow cytometry plots and (**C**) frequencies of $CD3^+CD20^+$ T cells within the total $CD3^+$ T cells following treatment with or without anti-CD3/CD28 Dynabeads and baricitinib (n = 6). (**D**) Representative flow cytometry plots and (**E**) frequencies of $CD4^+CD20^+$ T cells within the total $CD4^+$ T cells following treatment with or without anti-CD3/CD28 Dynabeads and baricitinib (n = 6). (**F**) Representative flow cytometry dot plots showing $CXCR5^+PD\text{-}1^+$ T cells gated on $CD4^+CD20^+$ T cells following treatment with or without anti-CD3/CD28 Dynabeads and baricitinib. (**G**) Frequencies of $CXCR5^+PD\text{-}1^+$ T cells within $CD4^+CD20^+$ T cells following treatment with or without anti-CD3/CD28 Dynabeads and baricitinib (n = 6). (**H**) Representative flow cytometry histograms and (**I**) MFI of intracellular IL-21 staining in $CD4^+$CD20+ T cells following treatment with or without anti-CD3/CD28 Dynabeads and baricitinib (n = 6). (**J**) Representative flow cytometry histograms and (**K**) MFI of intracellular IFN-γ staining in $CD4^+CD20^+$ T cells following treatment with or without anti-CD3/CD28 Dynabeads and baricitinib (n = 6). All data are presented as the mean ± SD. One-way ANOVA with Tukey's post hoc test was used for C, E, G, I and K. $^*P < 0.05$, $^{**}P < 0.01$.

Supplemental tables

Supplemental Table 1. Demographic characteristics of patients with BP and healthy controls. Values are n (%) unless otherwise indicated.

Characteristics	Bullous pemphigoid<br>n = 36	Controls<br>n = 35
Female	17 (47%)	17 (49%)
Male	19 (53%)	18 (51%)
Age, yrs, mean(range)	68.9 (51-87)	58.6 (45-69)
Ethnicity	Chinese	Chinese
Anti-BP180-NC16A	+	−

Supplemental Table 2. Patient characteristics.

Number	Sex	Age (years)	Anti-BP180-NC16A (index value)
BP01	M	60	73
BP02	F	65	85
BP03	F	51	15
BP04	F	79	180
BP05	M	72	130
BP06	M	73	194
BP07	F	74	130
BP08	F	76	180
BP09	F	73	220
BP10	M	77	185
BP11	F	70	100
BP12	M	61	90
BP13	M	53	43
BP14	M	77	59
BP15	F	51	71
BP16	F	55	51
BP17	M	67	67
BP18	F	76	96
BP19	M	78	206
BP20	F	75	22
BP21	M	52	126
BP22	M	69	162
BP23	F	73	65
BP24	F	84	193
BP25	M	72	93
BP26	M	85	35
BP27	M	75	190

BP28	F	52	148
BP29	M	75	132
BP30	F	87	10
BP31	M	78	80
BP32	F	61	12
BP33	M	57	34
BP34	M	65	13
BP35	F	62	53
BP36	M	73	84

Supplemental Table 3. Sequences of primers for real-time PCR.

Primer	sequences
Human	Forward 5'-TGGCCTCCGCCATCTTAGTA-3'
CD3	Reverse 5'-CCCCCAAACGCCAACTGATA-3'
Human	Forward 5'-TCCTTCTCCAACGCTGAGTC-3'
CD19	Reverse 5'-CAGGGAGGTTGCTTCCCG-3'
Human	Forward 5'-CCTTGGAGACTCAGGAGTTTTG-3'
MS4A1-1	Reverse 5'-GGCCCACCAGTGAAGACAT-3'
Human	Forward 5'-CCGGCAGAGCCAATGAAAG-3'
MS4A1-2	Reverse 5'-CAGATGGGTGCATAGATCCCT-3'
Human	Forward 5'-AACCAGCTAATCCCTCTGAGAA-3'
MS4A1-3	Reverse 5'-AGGCAAAGATCAGCATCACTG-3'
Human	Forward 5'-GTCTCCTCTGACTTCAACAGCG-3'
GAPDH	Reverse 5'-ACCACCCTGTTGCTGTAGCCAA-3'
Human	Forward 5'-GCCGCCAGCTCACCAT-3'
β-actin	Reverse 5'-TCGTCGCCCACATAGGAATC-3'
Human	Forward 5'-GGACCAGCAGGACAGGTATT-3'
PAX5	Reverse 5'-ATGGAACTGACGCTAGGCAC-3'

Supplemental Table 4. Sequence of overlapping peptides spanning the entire extracellular NC16A region of BP180 (490 – 566); Numbering starts from the N terminus.

NC-16A peptide number	Peptide sequence
1 (aa490-503)	EEVRKLKARVDELE
2 (aa492-506)	VRKLKARVDELERIR
3 (aa495-509)	LKARVDELERIRRSI
4 (aa498-512)	RVDELERIRRSILPY
5 (aa501-515)	ELERIRRSILPYGDS
6 (aa504-518)	RIRRSILPYGDSMDR
7 (aa507-521)	RSILPYGDSMDRIEK
8 (aa510-524)	LPYGDSMDRIEKDRL
9 (aa513-527)	GDSMDRIEKDRLQGM
10 (aa516-530)	MDRIEKDRLQGMAPA
11 (aa519-533)	IEKDRLQGMAPAAGA
12 (aa522-536)	DRLQGMAPAAGADLD
13 (aa525-539)	QGMAPAAGADLDKIG
14 (aa528-542)	APAAGADLDKIGLHS
15 (aa531-545)	AGADLDKIGLHSDSQ
16 (aa534-548)	DLDKIGLHSDSQEEL
17 (aa537-551)	KIGLHSDSQEELWMF
18 (aa540-554)	LHSDSQEELWMFVRK
19 (aa543-557)	DSQEELWMFVRKKLM
20 (aa546-560)	EELWMFVRKKLMMEQ
21 (aa549-563)	WMFVRKKLMMEQENG
22 (aa552-566)	VRKKLMMEQENGNLR

Supplemental Table 5. Bisulfite sequencing PCR primer sequences for methylation analysis.

Primer	Sequences (5'-3')	Product size
Promoter-5UTR-1	Forward - TGAGGATAATATTTGGGGATTTTTA	218
	Reverse - AACCCCCACCCTATAAACAAATATA	
Promoter-5UTR-2	Forward - AAATTTGGTAAGGTAGAAATTTTTT	253
	Reverse - CTAAATCTCCAAAACCTCAAATCTC	
CDS1	Forward - AGAAATTTAGTAAATGGGATTTTTT	146
	Reverse - CCCAAAATCTTAAATTCCCTCATA	
CDS3	Forward - TTTTTTTTATTTTTTTTATTTTTTTT	171
	Reverse - TTTTTAAAAATCTCAATTTTTAACC	
CDS5	Forward - AGTGATGTTGATTTTTGTTTTTTTT	183
	Reverse - CAACAACTCATCAAATTACATTCTC	
CDS6-1	Forward - GAAGAGGAAGAAGAAGAAATAGAGA	223
	Reverse - TCACTCCATACAAAAACCAAATAAA	
CDS6-2	Forward - TGGGAGGTTTTTATAGTTTTGAGTT	198
	Reverse - TTTCCTTCTCTCATATATTTCAATC	
