# Supplementary material for: CD20+ T follicular helper–like cells drive antigen-specific autoimmunity in bullous pemphigoid
Source: J Clin Invest. 2026 Jul 7;136(17):e197665. doi: 10.1172/JCI197665 (PMC13528933; doi:10.1172/JCI197665)

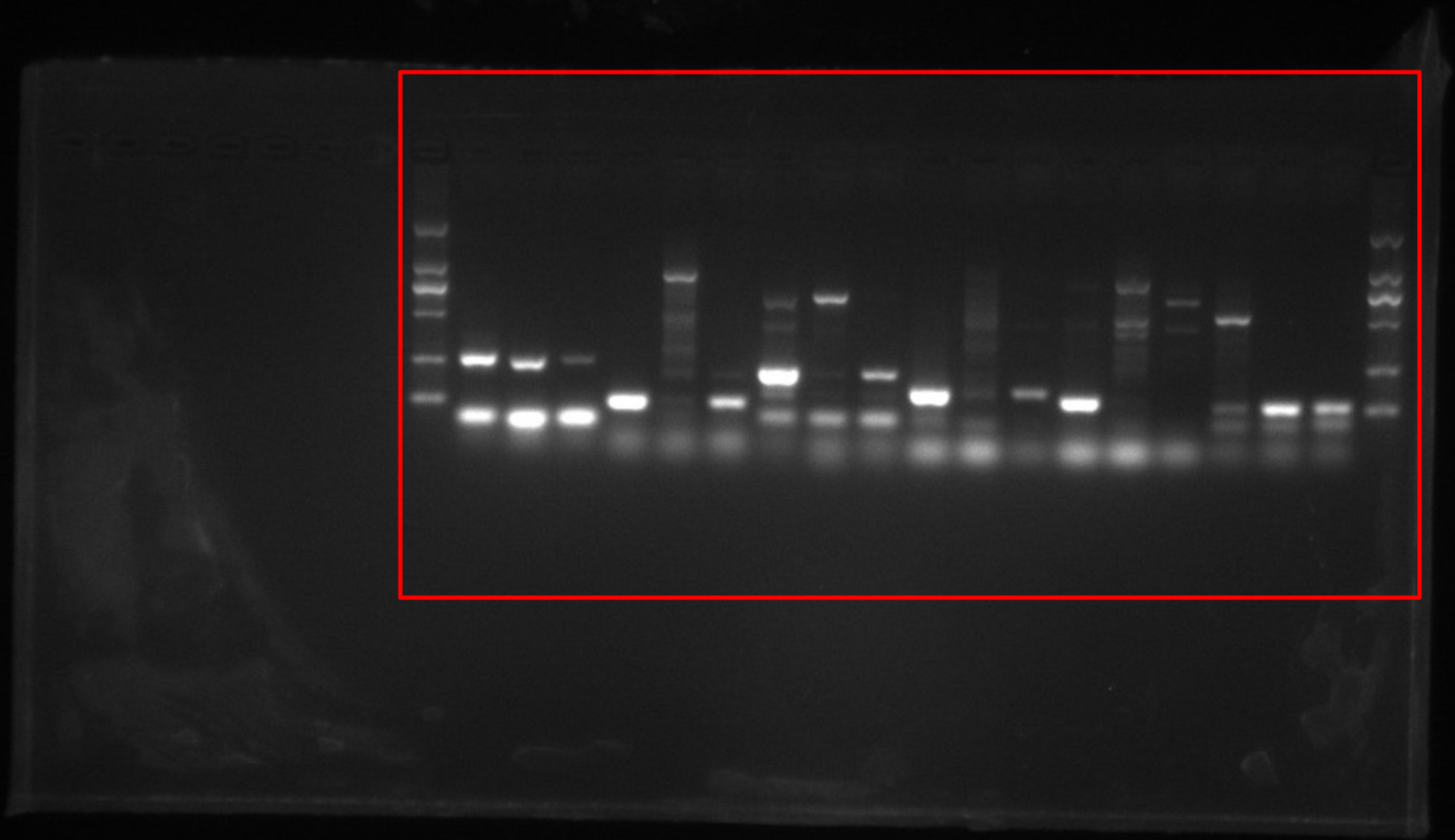

Supplement: Supplementary file 2 [file jci-136-197665-s230.pdf]
